# Global change in the trophic functioning of marine food webs

**DOI:** 10.1371/journal.pone.0182826

**Published:** 2017-08-11

**Authors:** Aurore Maureaud, Didier Gascuel, Mathieu Colléter, Maria L. D. Palomares, Hubert Du Pontavice, Daniel Pauly, William W. L. Cheung

**Affiliations:** 1 Université Bretagne Loire, Agrocampus Ouest, UMR 985 Ecology and ecosystem health, Rennes, France; 2 Nippon Foundation-Nereus Program, Institute for the Oceans and Fisheries, The University of British Columbia, Vancouver, British Columbia, Canada; 3 Sea Around Us, Institute for the Oceans and Fisheries, The University of British Columbia, Vancouver, British Columbia, Canada; Aristotle University of Thessaloniki, GREECE

## Abstract

The development of fisheries in the oceans, and other human drivers such as climate warming, have led to changes in species abundance, assemblages, trophic interactions, and ultimately in the functioning of marine food webs. Here, using a trophodynamic approach and global databases of catches and life history traits of marine species, we tested the hypothesis that anthropogenic ecological impacts may have led to changes in the global parameters defining the transfers of biomass within the food web. First, we developed two indicators to assess such changes: the Time Cumulated Indicator (TCI) measuring the residence time of biomass within the food web, and the Efficiency Cumulated Indicator (ECI) quantifying the fraction of secondary production reaching the top of the trophic chain. Then, we assessed, at the large marine ecosystem scale, the worldwide change of these two indicators over the 1950–2010 time-periods. Global trends were identified and cluster analyses were used to characterize the variability of trends between ecosystems. Results showed that the most common pattern over the study period is a global decrease in TCI, while the ECI indicator tends to increase. Thus, changes in species assemblages would induce faster and apparently more efficient biomass transfers in marine food webs. Results also suggested that the main driver of change over that period had been the large increase in fishing pressure. The largest changes occurred in ecosystems where ‘fishing down the marine food web’ are most intensive.

## Introduction

Anthropogenic stressors, such as fishing [1], degradation of essential habitats, pollution, intense activities on coastal areas, invasive species and climate change [2,3] disrupt marine species and ecosystems, and modify the structure and functioning of their food webs [4–6]. In particular, the rapid development of global fisheries since the 1950s [7–9] has led to a decline in predator biomass [10–12], overexploitation and collapse of fish stocks [8], and degradation of marine habitats [13,14]. Climate change is modifying ecosystems structure and functions [15] as ocean warming affects marine species’ size, growth, reproduction, distribution and interactions [16]. These stressors may act in synergy to modify the functioning of marine ecosystems [17,18]. Such changes deserve to be understood in the perspective of implementing the ecosystem approach to fisheries [19] and ecosystem-based management [20].

Biomass (or energy) flows from low to high trophic levels of a food web and their changes over time is a key aspect of ecosystem functioning, integrating the effects of natural and human disturbances [21]. The biomass flow in an ecosystem is dependent on species’ traits such as trophic levels, production or consumption rates and their interactions, as well as features of the physical environment such as temperature [22]. Ecosystem stressors, particularly fishing, can drive natural selection in exploited fish populations, as the sustained targeting of fish above a certain size has been shown to select for individuals with lower asymptotic size and earlier maturation [23]. Such selection events affect the structure and diversity of communities in the long term [17], and thus biomass flows in the food web.

In this paper, we investigate the past changes in the trophic functioning of marine ecosystems caused by the human-induced changes in species assemblages. We examine these changes at the scale of the World’s large marine ecosystems (LMEs) over the 1950 to 2010 period. For this, two parameters, defining the main characteristics of the biomass flow, were used: the trophic transfer efficiency [24,25] and the residence time of biomass in a food web [21,26]. Trophic transfer efficiency measures the fraction of biomass transferred by predation from one trophic level to the next [24], while the residence time of biomass refers to the time a unit of biomass spends at a given trophic level, before ascending to the next [26]. In other words, these parameters depend on species assemblages and measure ‘how much’ of the biomass flow is transferred from one trophic level to the next in the food web (transfer efficiency), and ‘how fast’ the biomass is transferred in the food web (residence time).

We proceeded in four steps: (i) we calculated the trophic transfer efficiency and the residence time of biomass within each large marine ecosystem using global fisheries catch data per taxon over the 1950–2010 period; (ii) from these parameters, we derived time-series of integrated indicators of the ecosystem trophic functioning, at the scale of the whole food web within each LME; (iii) we analyzed how these indicators changed over time, identifying global trends and their relationship with changes in fishing and climate; (iv) we used cluster analyses to explore the inter-LMEs variability in trends and to identify groups of ecosystems characterized by similar trends. The study of the ecosystem characteristics and changes within each cluster provides a new perspective for understanding the effects of natural and anthropogenic stressors on the marine food web functioning. The two new indicators and the methodology presented in this study provide a way to calculate food web functioning metrics and assess their temporal variability at the food web scale.

## Materials and methods

### Study area and data

The analysis is based on catch data for 1950 to 2010, assembled by the *Sea Around Us* fisheries catch reconstruction (see www.seaaroundus.org; [27]). Note that this database, while presenting estimates of fisheries catches by over 200 countries and their oversea territories, thousands of taxa and four sectors (industrial, artisanal, subsistence and recreational fisheries) and explicitly accounting for discards, builds on the Food and Agriculture Organization (FAO) records of global fisheries landings [27].

Parameters were calculated at the scale of the large marine ecosystems (LMEs). This geographic breakdown, initially defined by Sherman [28], now contains 66 ecosystems defined by their bathymetry, hydrography, productivity, species assemblages and coastal area limits (www.lme.noaa.gov; S1 Appendix). The 200 mile Exclusive Economic Zones of maritime countries, which yield about 90% of the global fisheries catch [29], are largely represented by the system of LMEs. However, due to mainly the unreliability of Chinese fisheries statistics [30] and the unavailability of detailed catch data from Siberia and other Arctic seas, the following LMEs were omitted from this study: East China Sea, Yellow Sea, Chukchi Sea, Beaufort Sea, East Siberian Sea, Laptev Sea, Kara Sea, Antarctic, Hudson Bay and Central Arctic Ocean [31–33].

We excluded rare taxa by including only the taxonomic groups that represented at least 0.1% of the total catches for at least one year between 1950 and 2010. Then, we collated biological information for the exploited taxa included in the catch database. Trophic level estimates for each species or taxon and the parameters required as input for empirical equations (such as growth parameters and ecological features) were taken from FishBase (www.fishbase.org; [34]) for fish and from SeaLifeBase (www.sealifebase.org; [35]) or the EcoBase repository [36,37] for invertebrates. For each LME and taxon, we used estimates of growth parameters originating from the same ecosystem when available, and average values from related taxa or larger geographical entities when not.

### Estimation of biomass flow parameters from growth parameters

The residence time of biomass is a key parameter of the trophic functioning of ecosystems, as implemented in the EcoTroph modelling approach [21], and is related to the life expectancy of organisms [38]. Here, we first considered the inverse of the residence time, i.e., the speed of the biomass flow, as a measure of the velocity of transfers within the food web from low to high trophic levels [39]. Gascuel et al. [39] showed that the speed of the biomass flow can be identified as the production to biomass ratio (P/B) and can be estimated for any fish species using the following empirical equation:

$${\left(\frac{P}{B}\right)}_{i,j}=1.06\times e^{0,018\times T_{j}}\times K^{0.75}{}_{i,j}$$ (1)

where B is the biomass, P the production, (P/B)_i,j_ is the speed of flow (expressed in TL.year^-1^) for taxonomic group *i* in LME *j*, T_j_ is the mean sea surface temperature (SST) in °C, and K_i,j_ is, in the von Bertalanffy growth model, the rate at which asymptotic size is approached.

**Fig 1:**
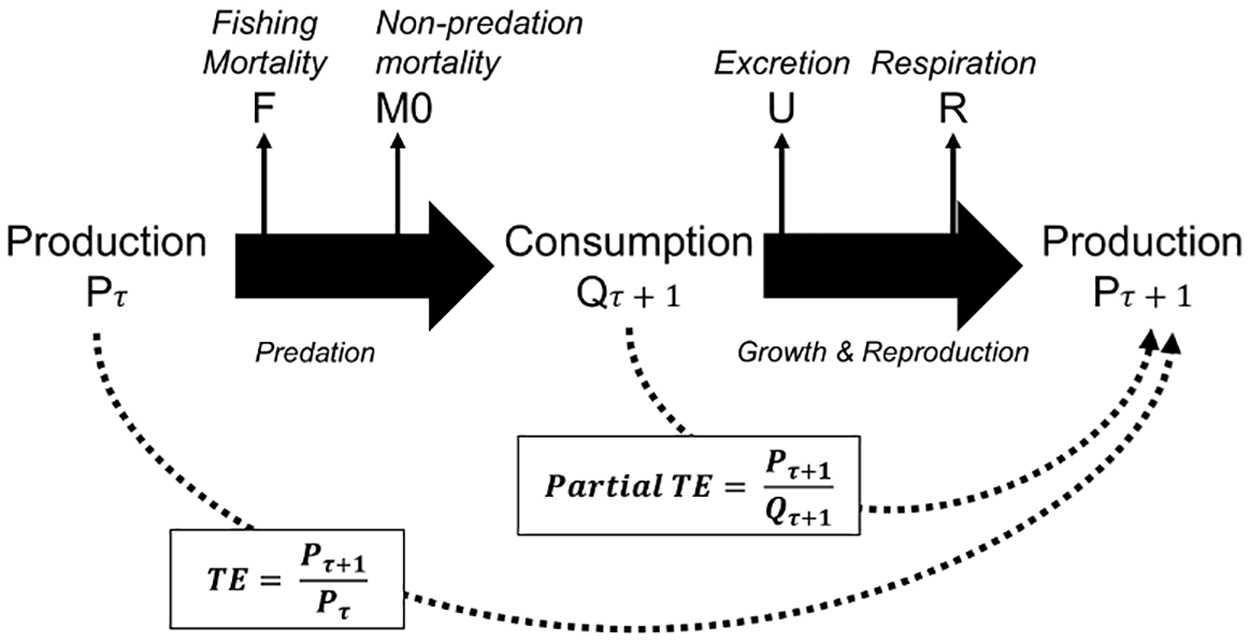
Biomass flow transfers between a prey and a predator. Black arrows represent energy transfers or losses. The prey has a trophic level τ and the predator has a trophic level (τ+1). Fishing mortality F and natural mortality M0 represent non-predation mortalities. Excretion U and respiration R are the predator metabolic losses. The partial transfer efficiency (P_τ+1_/Q_τ+1_) and total transfer efficiency (P_τ+1_/P_τ_) are indicated (derived from Gascuel et al.,[39]).

The second parameter of interest is trophic transfer efficiency, which is commonly defined as the ratio between the production of a predator and the production of its prey (Fig 1) and expresses the fraction of production that is transferred from one trophic level to the next in a food chain [24,40]. Here, we calculated a partial trophic transfer efficiency, the ratio P/Q (where Q is the consumption) also called “gross food conversion efficiency” [25], and whose value is largely determined by the respiration rate (R). We assumed that this P/Q ratio is related to total transfer efficiency, but this assumption will be further discussed. P/Q was estimated from the ratio between P/B (Eq 1) and Q/B [41]:

$${\left(\frac{Q}{B}\right)}_{i,j}=10^{7.964\;-\;0.204\;\times \;log_{10}\left(W_{i,j}\right)\;-\;1.965\;\times \;\frac{1000}{T_{j}}\;+\;0.083\;\times \;A_{i}+\;0.532\;\times \;h\;+\;0.398\;\times \;d}$$ (2)

where (Q/B)i,j is the biomass-specific consumption rate for taxonomic group *i* in LME *j*, Wi,j is the asymptotic weight of the von Bertalanffy growth curve, A is the aspect ratio of the fish caudal fin, h is 1 if the species is herbivorous and 0 otherwise, d is 1 if the species eats detritus and 0 otherwise.

Mean ecosystem SST (Tj), required for Eqs (1) and (2), was obtained from Eppley Laboratory (sbir.nasa.gov) as an average for the time-period 1980–2014. Because the study focused on community level changes, no variation of the temperature was included in the empirical equations; the von Bertalanffy growth parameters were also assumed to remain unchanged. These assumptions allowed us to focus on the changes in food web functioning in relation to changes in species composition, while we did not explicitly account for the effects of changes in diet, behavior or temperature on individuals or populations. For invertebrates, parameters P/B and P/Q have been directly estimated from a meta-analysis using the EcoBase repository [36,37].

### Trophodynamic transformation: From species to trophic spectra

**Fig 2:**
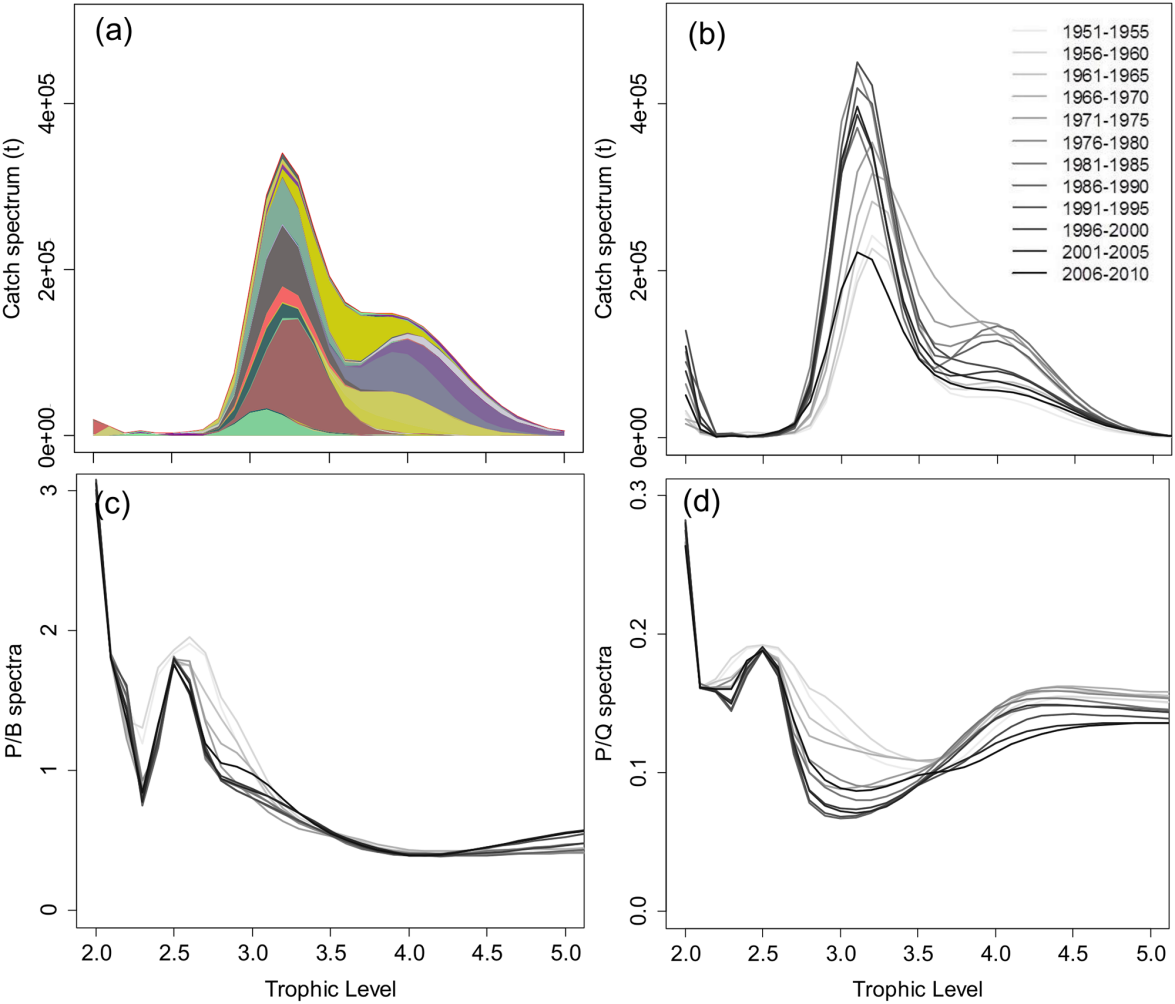
Examples of trophic spectra for the North Sea large marine ecosystem. (a) Catch Spectrum in 1970 where each colored area represents a smoothed species-specific catch; (b) Catch trophic spectrum, where each curve represent the mean spectrum over 5 years; light grey to black lines from 1951–1955 to 2006–2010 (c) Speed of flows P/B spectrum from 1951 to 2010 (d) Partial transfer efficiency P/Q trophic spectrum from 1951 to 2010.

To implement our trophodynamic approach, taxon-specific data were transformed into data per trophic class, building for each parameter a trophic spectrum according to an established methodology [38,42] implemented in the R package EcoTroph [43]. For a value of interest (usually biomass or catch; production P and consumption Q in our case), a smoothing function distributed the value for each species over a range of trophic classes, using classes with a width of 0.1 trophic level unit and a log-normal distribution assumed to mimic the within-species variability of trophic levels (Fig 2). This transformation from species-specific values into trophic classes considers explicitly the structure of the food web, and gives the distribution of the values of interest along the food web structure.

Trophic spectra were obtained from the sum of all species values (weighted by their catch) per trophic class (Fig 2). Weighted averages proportional to the catch for each trophic class were used for the ratios between P/B and P/Q:

$${\left(\frac{P}{B}\right)}_{\tau ,\;j,y}=\;\frac{\sum_{i}\left[\frac{P}{B}\;\times Y\right]i,\tau ,j,y}{\sum_{i}Y_{i,\tau ,j,y}}$$ (3)

$${\left(\frac{P}{Q}\right)}_{\tau ,\;j,y}=\frac{\sum_{i}\left[\frac{P}{B}\;\times Y\right]i,\tau ,j,y}{\sum_{i}\left[\frac{Q}{B}\;\times Y\right]i,\tau ,j,y}$$ (4)

where P/Q, is the partial transfer efficiency and Y, is the fisheries catch, referring to species *i*, at trophic level *τ*, in LME *j* and for year *y*.

### From trophic spectra to ecosystem indicators

The two parameters were raised from trophic classes to the entire food web, using two cumulated indicators. Based on preliminary testing, these indicators were calculated between trophic levels 2 and 4, as follows:

- The Time Cumulated Indicator (TCI) is the sum of all partial residence times within each trophic class:
  $$TCI_{j,y}=\;\Sigma_{\tau =2.0}^{\tau =4.0}\frac{\Delta \tau }{{\left(\frac{P}{B}\right)}_{\tau ,j,y}}$$ (5)
  where Δ*τ* is the width of the trophic class (i.e. 0.1). Thus, TCI_i,j_ expresses the residence time of biomass in the food web from TL = 2.0 to 4.0, in LME *j*, and for year *y*.
- The Efficiency Cumulated Indicator (ECI) quantifies the fraction of production passing from TL = 2.0 to TL = 4.0:
  $$ECI_{j,y}=\;\prod_{\tau =2.0}^{\tau =4.0}{\left(\frac{P}{Q}\right)}_{\tau ,j,y}^{\Delta \tau }$$ (6)
  Finally, indicators were expressed in relative values, relatively to 1950, in order to standardize indicators for all ecosystems and to provide an overview of temporal variations of trophic functioning within each LME:
  $$TCI_{R\;\;\;\left(j,y\backslash 1950\right)}=\;\frac{TCI_{j,\;y}}{TCI_{j,\;1950}}$$ (7)
  and:
  $$ECI_{R\;\;\;\left(j,\;y\backslash 1950\right)}=\;\frac{ECI_{j,y}}{ECI_{j,1950}}$$ (8)

### Data analysis

The expansion of fishing pressure for each ecosystem since 1950 was quantified using three indices: the primary production required for fisheries divided by the primary production (PPR/PP) of the ecosystem [44]; the index of the loss of production caused by fishing (L_index_) [45]; and the percentage of overexploited and collapsed stocks estimated from the stock status plots (SSPs) [46]. As complementary qualitative fishing indices, the mean trophic level of catch (MTL) [4], the fishing in balance index (FiB) [47], and the catch biodiversity index of Shannon (S) [48] were considered. These different indices were useful to distinguish potential bias due to fisheries catch data from specific processes due to ecosystem change [49] and also give an overview of the whole food web. For instance, an increase in the Shannon index can emphasize a diversification of targeted species by fisheries and an increase in the FiB index indicates an expansion of fisheries. Additionally, the correlation between the indicator and the Shannon biodiversity index has been tested (S1 Table).

Several indicators were also tested regarding climate effects: mean SST and primary production (PP) from the Eppley Laboratory (sbir.nasa.gov), and annual mean SST, PP and O_2_ from the Geophysical Fluid Dynamics Laboratory (GFDL CMIP5 MR, NOAA, www.gfdl.noaa.gov). The mean values were used to distinguish between different LMEs, while annual time-series data helped identify multi-decadal trends and long-term climatic signals across ecosystems.

Fifty-six LMEs were analyzed for which we had temporal series of ECI and TCI for 61 years (see data in S2 Table). We first conducted an analysis of worldwide trends of our two ecosystem indicators of trophic functioning (TCI and ECI), as well as fishing pressure and climate change indicators. Furthermore, we clustered the most strongly exploited ecosystems (10 ecosystems with the highest mean percentage of overexploited/collapsed stocks on the period 1950–2010 according to the SSPs; S1 Appendix) and the ecosystems with the most intense climate change effects (10 ecosystems with the highest SST increase between 1980–1985 and 2005–2010; S1 Appendix) to identify patterns characteristic of ecosystems with extreme fishing/climate change impacts, and compared to worldwide trends in efficiency/residence time of biomass.

In order to analyze the variability in trends between LMEs, and to better identify the effects of the various stressors, a Principal Component Analysis (PCA) and an Ascending Hierarchical Classification (AHC) of the 56 LMEs were performed independently on both TCI_R_ and ECI_R_. We used the R package FactomineR [50], considering indicators as the variables to be explained, LMEs as statistical levels and the years from 1951 to 2010 as explanatory variables. Quantitative and qualitative supplementary variables were added to each ecosystem, testing the effect of the ecosystem fishing regimes, climate change intensity, and their changes over time (supplementary variables and modalities are detailed in S1 Table).

### Sensitivity analysis

We conducted sensitivity analyses to assess the structural and parameter uncertainties of the results. Firstly, a comparison between a catch-based approach and a biomass-based approach was made using the North Sea as a case study. Catch and biomass values were extracted from a trophodynamic model (Ecopath model) of the North Sea [51]. The catch and biomass estimates were then used to calculate trophic spectra of P/B, P/Q and the related indicators (presented in S2 Appendix). Secondly, we used the established clusters and analyzed the indicators trends when including finfish species only in order to examine the effect of invertebrates’ fisheries development [52] on the indicators trends (presented in S3 Appendix). Finally, we developed a case by case analysis on a selection of contrasted LMEs, testing at the same time how indicators changed when calculated on various ranges of trophic levels (partial ECI, ECI_R_, TCI and TCI_R_ from TL = 2.0 to TL = b, where b varies between 2.5 to 4.5). This analysis (presented in S4 Appendix) also provides insights on the way trophic functioning is affected in different trophic levels.

## Results

### Worldwide trends in the trophic efficiency and residence time

**Fig 3:**
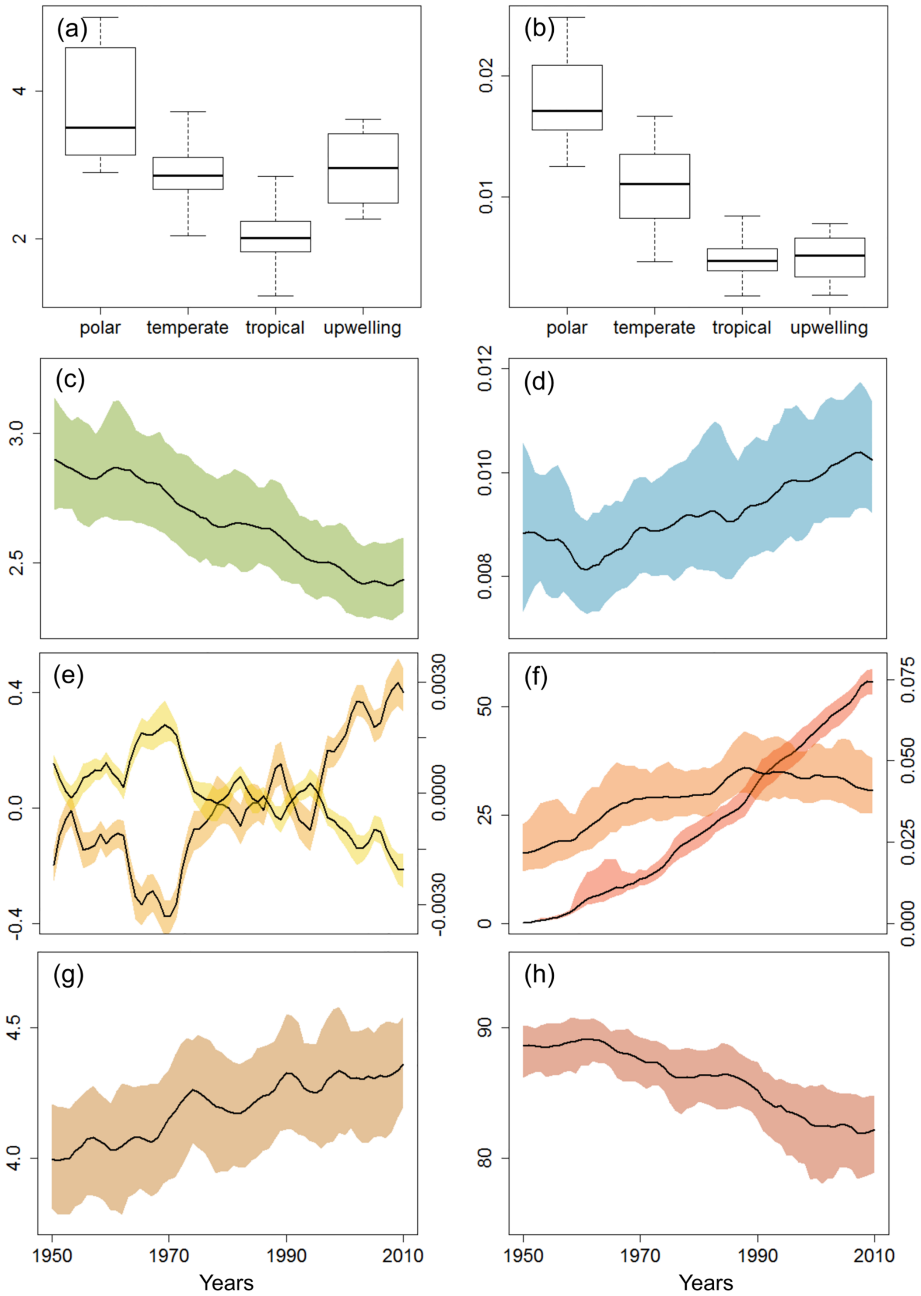
Worldwide values and trends of transfer efficiency, residence time, fishing and climate indicators. (a) Mean 1950–2010 values of the Time Cumulated Indicator TCI per type of ecosystem (in years); (b) Mean 1950–2010 values of the Efficiency Cumulated Indicator ECI per type of ecosystem; (c) Worldwide trend in TCI; (d) Worldwide trend in ECI; (e) Worldwide SST anomaly relative to the mean of the time-series (orange, in °C, left axis) and O_2_ anomaly trends (yellow, in mol O_2_ m^-3^, right axis) (f) Worldwide trends in the fishing pressures indices: SSP (red, in % on the left axis) and L_index_ (orange, right axis); (g) Shannon index (h) Percent of fish species. Colored sectors refer to bootstrap confidence intervals of the mean of LMEs, at 95%.

The TCI and ECI values among LMEs depend in part on the type of ecosystem (Fig 3). The slowest biomass transfers in the food web were observed for polar ecosystems (TCI>3.5 years on average, from TL = 2 to TL = 4) and the fastest for the tropical LMEs (TCI = 2 years). Polar ecosystems had the most efficient trophic transfers, while tropical ecosystems exhibited low efficiencies between TL = 2 and TL = 4. The mean partial transfer efficiency over one trophic level was estimated to be 13%, 10% and 7% in polar, temperate and tropical LMEs, respectively. Upwelling ecosystems (4 LMEs: California Current, Humboldt Current, Canary Current and Benguela Current) exhibited intermediate TCI values and low efficiencies.

The mean global trend among all LMEs showed a significant and continuous decrease in TCI from 2.9 years in 1950 to less than 2.5 years in 2010 (Fig 3). The worldwide average ECI increased from less than 0.009 in 1950 to more than 0.010 in 2010 (Fig 3), which corresponds to 9.5% and 10.15% per trophic level, respectively. Both global trends are significantly changing, as suggested by the statistical analysis (S5 Appendix).

The pressure and status indicators showed that the ocean was getting warmer, less oxygenated, with more fish stocks becoming overexploited/collapsed and catches more diverse. SSTs increased by 0.6°C from the 1950s to 2010, while oxygen content decreased by 0.003 mol O_2_.m^-3^ during the same period (Fig 3). The stocks that were classified as overexploited or collapsed increased from zero in the 1950s to 56% in 2010, whereas the L_index_ increased substantially from the 1950s to the 1990s and then stabilized (Fig 3). The Shannon index, expressing the diversity of species in the catches, increased since the 1950s, along with a decrease of the contribution of finfish in fisheries catches (Fig 3).

**Fig 4:**
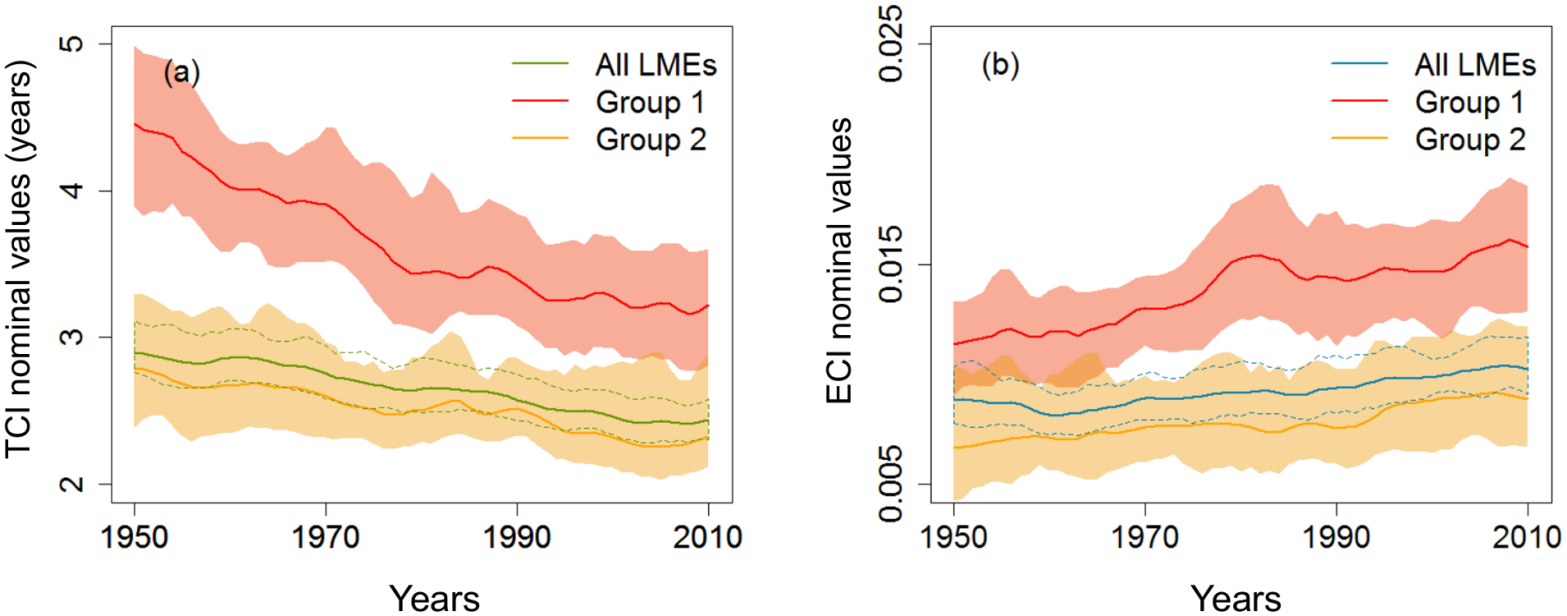
TCI and ECI trends on an a priori selection of LMEs. (a) Time indicator nominal values trends (b) Efficiency indicator nominal values trends–‘Group 1’ gathers the 10 most strongly exploited ecosystems (according to their SSPs); ‘Group 2’ gathers the 10 ecosystems with the strongest increase in SST since 1990; ‘All LMEs’ gathers the 56 LMEs. Colored sectors refer to bootstrap confidence intervals of the mean, at 95%.

The average residence time of biomass and trophic transfer efficiencies of the ten most strongly exploited ecosystems were higher than the global mean (Fig 4). These ecosystems were polar or temperate ecosystems, mainly in the North Atlantic (S1 Appendix). The indicators were not only higher in these overexploited ecosystems, but also showed stronger variations since the 1950s. The residence time of biomass in the food webs fell from 4.5 to 3.2 years over the study period, a much stronger decrease than the worlwide trend (-28% and -16%, respectively). The trophic efficiency indicator increased by 39% (from 10.7 to 12,5% when expressed over one TL), generally higher than the worldwide trend (+15%; from 9.4% to 10.1% over one TL). In contrast, the 10 LMEs selected for their important rise in SST, did not appear to exhibit any particular pattern in their functioning when compared to the worldwide trends.

### Faster biomass transfers in food webs

**Fig 5:**
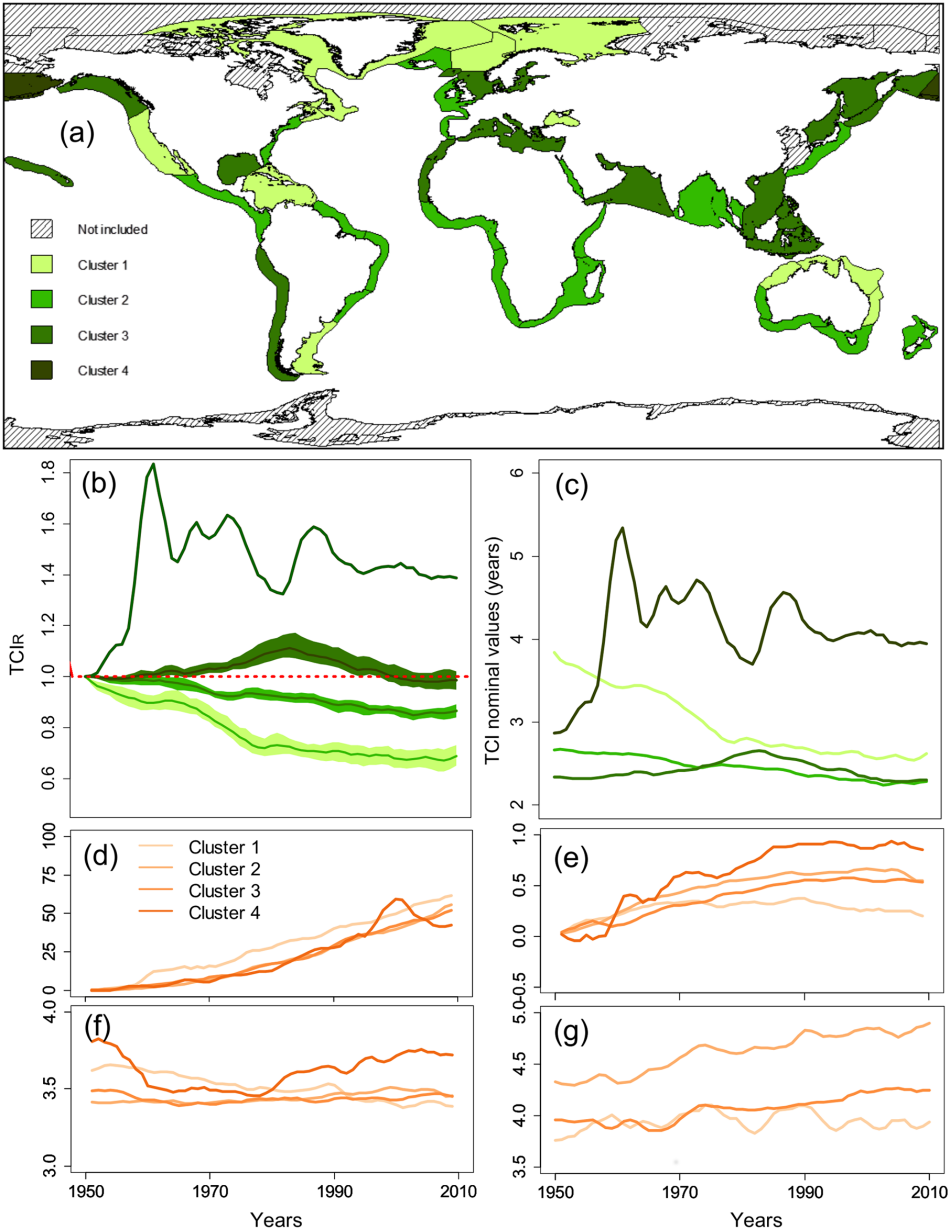
Results from the Principle Component Analysis and clustering on the Time Cumulated Indicator TCI_R_. (a) Worldwide map of the clusters; Mean trend per cluster from 1950 to 2010 in: (b) Relative to 1950 TCI_R_ (colored sectors refer to bootstrap 95% confidence intervals); (c) Nominal values of TCI; (d) Number of overexploited and collapsed stocks in SSPs (%); (e) FiB index; (f) the Mean Trophic Level; (g) the Shannon biodiversity index.

Four patterns of evolution in the time indicator were identified among the 56 LMEs from the Principle Component Analysis (Fig 5; Table 1). Clusters 1 and 3 were characterized by high fishing pressure, while cluster 2 was composed of more lightly exploited ecosystems. In cluster 4, the fisheries developed after the 1960s and very few catch occurred in the 1950s.

**Table 1:** Clustering based on trends in the Time Cumulated Indicator (TCI_R_) and the Efficiency Cumulated Indicator (ECI_R_): Selection of results regarding clusters description by supplementary qualitative variables.

	Indicator and Cluster	Qualitative modalities	Occurrence in all LMEs (%)	Selected in the cluster (%)	Occurrence in the cluster (%)	p-value
**TCI**_**R**_	**Cluster 1** (16 LMEs)	FiB decreasing	7.1	100.0	25.0	0.005**
		PPR/PP decreasing	8.9	80.0	25.0	0.021*
		MTL decreasing	44.6	44.0	68.8	0.028*
		Overexploitation>50% (1990–2010)	51.8	41.4	75.0	0.033*
		Prop. of fish species decreasing	58.9	39.4	81.3	0.036*
		FiB increasing	83.9	21.3	62.5	0.013*
	**Cluster 2** (21 LMEs)	MTL stable	51.8	51.7	71.4	0.027*
		Tropical ecosystems	48.2	51.9	66.7	0.038*
	**Cluster 3** (17 LMEs)	High Lindex >0.05 (1990–2010)	35.7	55.0	64.7	0.004**
		High catch >1t/km^2^ (1990–2010)	51.8	44.8	76.5	0.017*
		1t/km^2^ < Catch high <2t/km^2^	26.8	53.3	47.1	0.034*
		Lindex low (<0.01)	26.8	6.7	5.9	0.019*
	**Cluster 4** (2 LMEs)	High MTL (>3.60)	19.6	18.2	100.0	0.036*
		Polar ecosystems	21.4	16.7	100.0	0.043*
**ECI**_**R**_	**Cluster 1** (7 LMEs)	PPR/PP<10%	35.7	0.0	0.0	0.036*
		TCI decreasing	69.6	2.6	14.3	0.002**
	**Cluster 2** (29 LMEs)	+0.1°C< SST increase <+0.7°C	57.1	65.6	72.4	0.020*
		Temperate ecosystems	23.2	76.9	34.5	0.045*
		High Lindex >0.05 (1990–2010)	35.7	70.0	48.3	0.049*
		FiB decreasing	7.1	0.0	0.0	0.048*
		Prop. of cephalopods: 0–1%	46.4	34.6	31.0	0.020*
	**Cluster 3** (10 LMEs)	Catch increase	50.0	32.1	90.0	0.006**
		Tropical ecosystems	48.2	29.6	80.0	0.033*
	**Cluster 4** (9 LMEs)	Prop. of fishes decreasing	58.9	27.3	100.0	0.005**
		FiB decreasing	7.1	75.0	33.3	0.011*
		TCI decreasing	69.6	23.1	100.0	0.028*
		Correlation to Shannon >0.5	33.9	31.6	66.7	0.040*
		Lindex low <0.01 (1990–2010)	25.0	35.7	55.6	0.040*
		PPR/PP increasing	87.5	10.2	55.6	0.010*

Modalities are ranked from the over-represented to under-represented in each cluster. Detailed modalities tested can be found in S1 Table and detailed results can be found (de cumulativethe Tlated index (ECI)nce 1950e of biomass in S6 and S7 Appendices. P values measure if occurrence of the modality within the cluster differ from the one in the whole studied population;

‘*’ stands for p-value<0.05,

‘**’ for p-values<0.01.

Cluster 1 aggregated 16 ecosystems, including ecosystems that exhibited the highest fraction of overexploited or collapsed stocks and ecosystems that were already intensively exploited in the 1950s (mainly from polar regions and the North Atlantic), but where the fishing pressure decreased since the 1980s. In those ecosystems, the TCI sharply decreased from high values over the whole period, from 3.8 years in 1950 to 2.6 years in 2010 (Fig 5). At the same time, the amount of total catch and its diversity (Shannon index) remained almost constant at low levels while the mean trophic level of catch, the FiB index, and the fraction of fish species decreased over time. Such trends remained when only finfishes were considered in the analysis (Cluster1 in S3 Appendix), suggesting that the development of invertebrate fisheries was not the major driver of the indicator trends. However, when all species are included, the decrease is stronger, suggesting an influence from increasing invertebrates catch.

Fishing pressure was also important in cluster 3, as indicated by L_index_ and PPR/PP, and includes two upwelling, some temperate ecosystems such as the North Sea, and a majority of the East Asian LMEs. In this cluster, fast trophic transfers occurred in the 1950s, slowing in the mid-1980s (from 2.3 to 2.7 years), while the catch amount increased (from 1 to 2t·km^-2^). Since the late 1980s, the total catch and its diversity stabilized, while the TCI decreased from 2.7 to 2.3 years in the 2010s, revealing faster trophic transfers in the recent period.

Ecosystems in which fisheries developed later and targeting a larger diversity of species (e.g., tropical ecosystems; cluster 2), were associated with a lower fishing pressure. However, in those ecosystems, the residence time of biomass decreased from 2.7 years in 1950 to 2.3 years in 2010. In the East Bering Sea and the Aleutian Islands (cluster 4), the TCI strongly increased until the early 60s (from less than 3 years in 1950 to more than 5 years in 1962) due to the development of fisheries. Before 1960, very small catches were taken. The trend after 1960 was similar to those of clusters 1 or 2.

### Diversity of trends in trophic efficiencies

**Fig 6:**
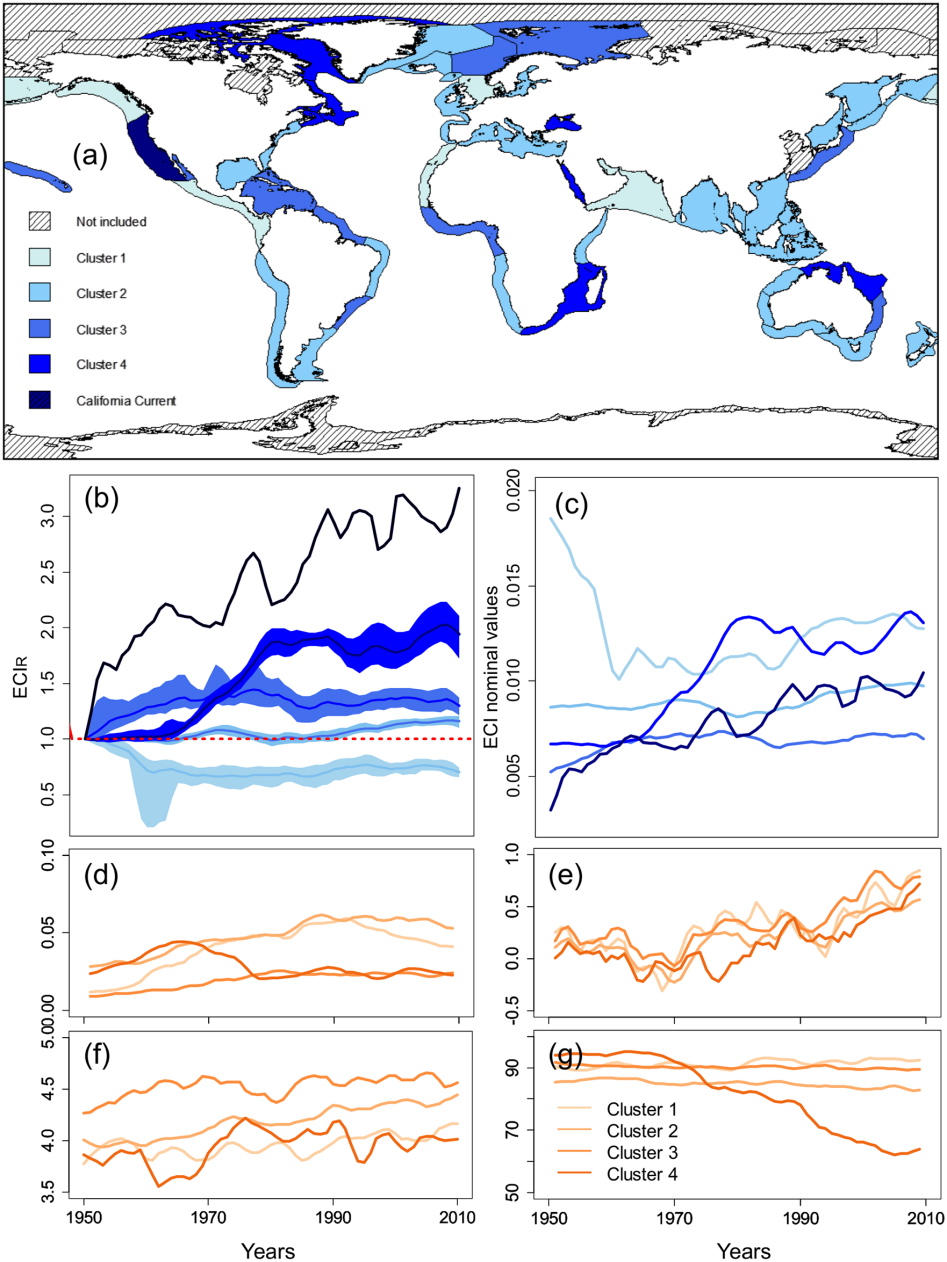
Results from the Principal Component Analysis and clustering on the ECI_R_. (a) Worldwide map of the clusters; Mean trend per cluster from 1950 to 2010 in: (b) Relative to 1950 ECI_R_ (colored sectors refer to bootstrap 95% confidence intervals); (c) ECI nominal values; (d) L_index_ of fishing pressure; (e) Difference SST_(y)_−SST_(1950)_; (f) the Shannon biodiversity index (g) Fraction of finfish species (%).

More disparity was found in the clustering of LMEs from the trophic transfer efficiency indicator, with greater increases than decreases since 1950 (Fig 6). The California Current forms a cluster by itself because of its increasing efficiency starting from very low efficiency values in the 1950s, when the catch of California sardine was highest.

Similar to cluster 1 in the TCI_R_ analysis, cluster 4, where fishing pressure was high in the early years and where the overexploitation or collapse was highly represented, exhibits large changes in functioning: trophic efficiency increased substantially (from 8.2% in the 1960s to 11.4% per TL in 2010). The increase mainly occurred over the 1970s, jointly to a large decrease in the L_index_, FiB index and MTL. Also, its correlation with the Shannon diversity index was positive for the majority of LMEs, while the fraction of fish in total catches decreased (Table 1). The trend in cluster 4 was not only due to the emergence of invertebrates in the catch but also to changes among trophic class of finfish species (S3 Appendix).

Two other groups of ecosystems are under intensive fishing pressure: clusters 1 and 2. In cluster 1 (including the North Sea), where the total catch and L_index_ increased and reached high values over the time-period, the mean efficiency decreased from 13.7% to 11.2% per TL. The diversity of catch was rather low, while the fraction of fish species remained large and constant since 1950 (Table 1). In cluster 2, even though the fishing pressure was large, the trophic efficiency indicator was almost stable (increased from 9.3% to 9.9% per TL over the last two decades). In contrast to cluster 1, the diversity of the catch was high and increasing.

As for the time indicator, cluster 3, which includes a majority of tropical ecosystems, was characterized by a low but increasing fishing pressure and a high diversity in catch, especially during the two earlier decades. Efficiency increased slightly, from 7.3% per TL in 1950 to 8.3% in 1980, before stagnating over the second half of the study period. However, the trend of this cluster was not consistent when including only finfish species (S3 Appendix).

## Discussion

### Building indicators to explore the food web functioning

#### The Time Cumulated Indicator

TCI, expressed as the mean B/P ratio per trophic class, quantifies the residence time of biomass in the food web [26], and indicates how much time is required to transfer energy from TL = 2.0 to TL = 4.0. This indicator depends on the mean life expectancy of organisms at each trophic level: the shorter the life-expectancy, the more the fish are eaten at young age, and the faster the biomass moves up from a given trophic level to the next. In other words, what the indicator measures is not the overall fraction of short-living species in the ecosystem, but rather within each trophic class. Therefore, faster biomass transfers indicate that the fraction of short-living species increased in at least one trophic class (the impacted trophic classes was specified using partial TCI; S4 Appendix).

Although fishing obviously decrease the life expectancy of individuals in exploited stocks, the TCI indicator does not consider changes which may occur at the population level. This demographic effect will tend to reduce residence times in the food webs. As a consequence, the observed decrease in the indicator is considered an underestimate of the real trend which may have occurred over the period.

#### The Efficiency Cumulated Indicator

The ECI indicator is derived from the transfer efficiency and is commonly used in the field of trophic ecology, for instance, for the calculation of fishing pressure indicators [31,49] or in ecosystem models [11,53]. Here, using the P/Q ratio to build the Efficiency Cumulated Indicator ECI, we mainly focused on losses from respiration and excretion (Fig 1). However, we did not account for non-predation natural mortalities, for example, the transfer of energy from organisms to detritus after their death. Thus, ECI may over-estimate trophic transfer efficiency, particularly for organisms with less predation pressure or which are ‘trophic dead end/energy roundabout’ species [54]. In addition, we did not account for fishing mortalities because they relate to changes occurring at population level, while our study aims to focus on community level and on changes in species assemblages. However, as fishing removes part of the production from exploited populations, ECI may also over-estimate trophic transfer efficiency particularly in ecosystems where fisheries catches account for the removal of production from specific trophic levels over the last decades.

The geographic pattern exhibited by both indicators appeared globally consistent and seemed to confirm their ability to capture the variability in trophic functioning among ecosystems. The efficiency indicator ECI showed that LMEs tended to be more efficient in high latitudes and less efficient in the tropics (Fig 3), which had already been demonstrated in other studies [55,56]. As well, the most efficient ecosystems were also characterized by long residence time of biomass in the food web through TCI (Fig 3). This pattern is due to temperature, explaining that polar ecosystems generate slow biomass transfers compared to tropical ecosystems [25,26,53].

### Data uncertainties

TCI and ECI are based on catch data and the use of catch data to infer changes in biomass in the food web is an issue that is contentious in the scientific literature [57,58]. The sensitivity analysis performed on the North Sea revealed a large difference in P/B and P/Q trophic spectra for low trophic levels and in the resulting indicators (S2 Appendix). Such difference was expected since catch data allowed only to study the exploited part of the food web, which thus does not consider low trophic levels and especially phytoplankton. In contrast, for intermediate and high trophic levels, the spectra weighted by catch or by biomass were very close, because a large part of the biomass is accessible to fishing. Thus, our trophic indicators constitute acceptable proxies to study the trophic functioning of the fishable part of food webs only. However, some species were not well represented in catch even if important for the functioning of food webs. This was for instance the case of jellyfish, which influence the functioning of many ecosystems through competition with forage fish [54], and can drive ecosystem shifts [18,59].

Using catch data instead of biomass for indicators computations may have also led to bias due to changes occurring in fishing strategies or fishing regulations. Such type of bias can be detected in our study. For example, some ecosystems such as the Insular Pacific-Hawaiian demonstrated large shifts in the level and composition of catches, caused by changes in fisheries regulations introduced in the 1980s [60]. More generally, the decrease in the fraction of finfish species observed in some ecosystems (or clusters) suggests either a potential bias due to the development of fisheries targeting invertebrates since the 1950s [52], or a real increase in abundance of invertebrate species in some ecosystems due to fisheries effects or climate change [61,62]. However, the two effects can be disentangled using different indicators such as the FiB index [49]. Furthermore, the complementary analysis on finfish we performed demonstrates, at least partially, that the main trends observed for the various clusters (e.g. clusters 1, 2, 3 for TCI and clusters 1 and 4 for ECI) were not only due to a development of invertebrate fisheries (S3 Appendix). Additionally, conducting experiments could help to understand better the indicators [63].

### Towards faster and more efficient trophic transfers in marine food webs?

#### Trophic transfers are becoming faster

Results demonstrated a significant worldwide decreasing trend in TCI, which appeared to be a result, at least in part, of the global increase in fishing pressure. The decline was two times larger in the ten ecosystems where the fraction of overexploited/collapsed stocks is the highest (-28%), and -32% on average for the ecosystems in cluster 1. In this cluster, characterized by intense fishing, the large decrease in the MTL, FiB index, and fraction of finfish in the catch suggests a clear “fishing down the food web” syndrome [4,49]. Over-exploitation of top-predators may have progressively altered the structure of the ecosystem, leading to the dominance of small, fast-growing organisms. As a result, biomass transfers would drastically be faster at the scale of the whole food web and within trophic classes.

Ecosystems where fisheries developed more recently (cluster 2), for example in the South Hemisphere [7], also demonstrated a transition to faster biomass transfers since the 1950s. However, the increase in catch and in catch diversity suggest that our trophic indicators estimated from catches might be biased by “fishing through the food web” [64] and/or “fisheries expansion” [7,65,66]. Such processes, related to changes in the fishing strategies and targeted species, may have partially occulted the real changes occurring in ecosystems. Nevertheless, the sensitivity analysis on the development of invertebrate fisheries (S3 Appendix) indicates that those fisheries do not fully influence the trend in cluster 2, suggesting potential changes in food web functioning of the finfish community since 1950. As well, the fisheries development observed in cluster 4 since the 1970s is characterized by a clear increase in the mean trophic level of the catch, suggesting a “fishing up marine food webs” effects [67]. For clusters 2 and 4, even if the functioning changes are not directly detected, they might still occur and those clusters allow identifying real changes in the other clusters.

Results also highlighted that environmental forcing is a driver of some changes observed in the speed of trophic transfers. This is the case for upwelling ecosystems, such as the Humboldt Current and some Asian LMEs, where the large changes in the abundance of *Sardinops sagax* influenced the residence time of biomass in ecosystems. Those ecosystems explained the trend of cluster 3, where TCI increased when the sardine was abundant in ecosystems and in the catch (this species has a high residence time in its trophic class). This species abundance was closely linked to specific temperature regimes [68], eventually resulting in some change in the food web functioning.

#### Increasing indicator of the transfer efficiency?

Our results largely suggest that transfer efficiency is highly variable among ecosystems (from 4.2% per trophic level to 15.8%) and since 1950, which is concordant with other studies [44,69]. Some authors consider that stress could generate a decrease [70–72] or an increase [25,73] in trophic transfer efficiency. We observed a worldwide slight increase in the ECI, over the study period from 9.5% in 1950 to 10.2% in 2010. This result needs to be taken carefully, considering the influence of invertebrate fisheries (S3 Appendix). Here again, the increase in fishing pressure appeared to be the main driver of change. The cluster analysis showed that the largest rises in the mean ECI indicator are observed for ecosystems of cluster 4, where the fishing indicators suggested a very high fishing pressure and a clear case of “fishing down the marine food web” [4]. In this cluster, the fraction of secondary production reaching trophic level 4 (ECI) doubled since 1950.

The general rule of an increasing trophic efficiency over time has a few exceptions, particularly the 7 LMEs included in cluster 1, whose trophic efficiency was decreasing, especially during the first decades of the study period. These ecosystems were characterized by high fishing pressures, but also by a rather low and stable diversity in catch and a large fraction of finfish. These ecosystems were also exceptions to the general rule of a decreasing TCI_R_. The North Sea case study (S4 Appendix) exhibited a transition within several trophic compartments to less efficient species in term of trophic transfers and with identical/higher life expectancies.

#### Consequences of faster transfers and higher trophic efficiencies

The worldwide decrease in the residence time of biomass in the food web may have resulted in a decrease in the overall biomass of marine ecosystems, especially at high trophic levels. The shorter life expectancy of organisms results in faster trophic transfer through trophic level classes, and smaller biomass. Such a quasi-physical relationship between the speed of the trophic flow and the abundance of living organisms is predicted from numerical modeling of trophic dynamics [21,39,42]. At the community level, the fishing-induced selection of short-living species induces higher mean natural mortalities, thus adding to the direct impact of fishing, an indirect impact through changes of the life history traits of species assemblages.

Fishing-induced modification of the trophic structure may increase the resilience to anthropogenic pressures [39]. Fishing takes away the long-lived slow growing species, and leaves the fast growing short-lived species, which are less vulnerable to fishing [74]. Nevertheless, short-lived species are also more sensitive to environmental-driven fluctuations [68], leading to more variations in ecosystem structure and functions. This is consistent with E. Odum’s theory of ecosystem maturation [22,75], which predicts that perturbed ecosystems have more chaotic dynamics.

The detailed analysis of indicators related to fishing strategies showed that the general rule of an increasing transfer efficiency inferred from fisheries catch reflects realistic changes occurring in the food webs, at least in part. Such changes towards more efficient species can be interpreted as an ecosystem’s adaptation to stress, and more specifically to increasing fishing pressures. This change did not mean that the functioning of the food web was improving, especially in terms of biomass flow transferred to top-predators. First, we underlined above that our ECI indicator only relies on partial trophic efficiencies, not taking into account the increase in fishing mortality and the variability in the mean non-predation natural mortality. Such increases of non-predation mortality would contribute to reduce the size of the biomass flow available to the higher part of the food web. Second, even if the fraction of biomass flow reaching high trophic levels was increasing, the biomass flow may be reduced, eventually leading to a smaller total biomass transfer towards top-predators. This is what occurred, for instance, in the Newfoundland-Labrador Shelf ecosystem which exhibited a strong decline of the biomass [70], a higher ECI and reduced TCI. There is little doubt that such type of change affected the trophic functioning of marine ecosystems worldwide, leading to a global decrease in the biomass flow available for top-predators.

Our results also suggested that indicators are interdependent and that we see simultaneously transitions to faster/more efficient or longer/less efficient transfers. This assumption is not valid for all trophic class and ecosystems (as suggested in S4 Appendix), but it could be interpreted as an adaptive response of the food web to allow faster and more efficient transfers under ecosystem perturbation. The theoretical basis of such ecological responses to perturbation should be explored in future studies.

### Towards an increasing impact of climate change

Even if some climate change effects on marine communities have already been shown at the global scale [76], our study did not demonstrate any climate change impact. The selection of the 10 LMEs demonstrating the highest increase in SST since the 1980s did not exhibit any particular pattern. Another classification was tested based on observed temperature data [77], but did not show any particular pattern either. One reason may be that climate change effects on trophic functioning are harder to detect because of synergistic interactions with fisheries. Several studies showed that changes in community structure and ecosystem productivity in the recent decades have been driven by both climate and fisheries [17,18]. Another hypothesis is that fishing has been the dominant factor shaping trophic transfer in LMEs, and might be a predominant factor on ecosystems even in the recent decades. However, the impact of climate change should increase in the coming years, especially as large scale changes in species distribution and biogeography are predicted for the world oceans under climate change [78–80]. The consequences should be a higher abundance of short-living species in temperate and polar LMEs. Some scenarios also predict an increase of pelagic and demersal invertebrates [81]. Such changes could alter the functioning of marine food webs, in which faster transfers could appear. Furthermore, food webs exploited by fisheries might be more sensitive to climate change [17], to trophic cascades [5] and to shifts in the ecosystem species assemblages, and the resulting structure and functions. By considering another spatial resolution for the two indicators, climate change effects need to be further explored.

Overall, this study improves our understanding on the effects of fishing and environmental changes on the trophic dynamics of the world’s marine ecosystems. The results challenge the widely used assumption that trophic transfer efficiency is relatively constant across different ecosystems, time and trophic entities. Instead, we showed that trophic transfer varies between ecosystems, which is partly driven by different levels of disturbance. Our findings improve theoretical understanding of trophodynamics and the accuracy of ecosystem modelling, as well as their applications for understanding the effects of climate change or assessing fisheries management options.

## Supporting information

S1 Table Quantitative and qualitative supplementary variables tested in the clustering. (DOCX)

S2 Table Indicators TCI and ECI time-series per large marine ecosystem. TCI values are given in years in the green cells and ECI values in the blue cells. LME numbers are the official numbers and correspond to the ecosystems indicated in S1 Appendix.
(DOCX)

S1 Appendix Large marine ecosystems (LMEs) map and characteristics. The 66 LMEs are represented with the corresponding official names (from www.lme.noaa.gov). The 10 ecosystems that include the highest fraction of overexploited and collapsed stocks are represented in red (Group 1). The 10 ecosystems that demonstrate the highest increase in sea surface temperature between 1990 and 2010 are represented in orange (Group 2). The 10 LMEs excluded from the database are listed as ‘not included’.
(DOCX)

S2 Appendix Sensitivity analysis based on an Ecopath model for the North Sea. (DOCX)

S3 Appendix Sensitivity of the results to non-fish species’ influence on the clusters trends for the functioning indicators TCI and ECI. The trends for each cluster and indicator were plotted taking into account finfish species only, cumulating indicators from TL = 2.5 to TL = 4.0.
(DOCX)

S4 Appendix Case studies of species assemblages. (DOCX)

S5 Appendix Complementary statistical analysis on worldwide indicator trends. (DOCX)

S6 Appendix Complementary results of the clustering performed on TCI. (DOCX)

S7 Appendix Complementary results of the clustering performed on ECI. (DOCX)
