# Supplementary material for: Global change in the trophic functioning of marine food webs
Source: PLoS One. 2017 Aug 11;12(8):e0182826. doi: 10.1371/journal.pone.0182826 (PMC5553640; doi:10.1371/journal.pone.0182826)
Supplement: S1 Table — (DOCX) [file pone.0182826.s001.docx]

S1 Table. Quantitative and qualitative supplementary variables tested in the clustering.

| Variable | Type | Modalities/Calculation | | | | | | | | |
| --- | --- | --- | --- | --- | --- | --- | --- | --- | --- | --- |
| Habitat | Qualitative | Polar/Temperate/Tropical/Upwelling | | | | | | | | |
| Mean sea surface temperature SST | Quantitative | Mean SST from Eppley for each LME (1980-2014) | | | | | | | | |
|  | Qualitative | <8°C | | 8-16°C | | | >16°C | | | |
| Mean primary production PP | Quantitative | Mean PP from Eppley for each LME (1998-2014) | | | | | | | | |
|  | Qualitative | <500mgC.m^-2^ | | 500-1000 | | | | | >1000mgC.m^-2^ | |
| Difference in SST 1950-2000s | Quantitative | Mean of SST difference between year y and 1950 over 2000-2010 from GFDL model | | | | | | | | |
|  | Qualitative | < -0.1°C | -0.1 to 0.1 | | | 0.1 to 0.7 | | | | >0.7°C |
| SST relative to 1950 | Quantitative | SST(y)/SST(1950) from GFDL model | | | | | | | | |
|  | Qualitative | Decrease if <0.99 | | | Stable if  0.99 – 1.01 | | | Increase if  >1.01 | | |
| Oxygen relative to 1950 | Quantitative | O2(y)/O2(1950) from GFDL model | | | | | | | | |
|  | Qualitative | Decrease if <0.99 | | | Stable if  0.99 – 1.01 | | | Increase if  >1.01 | | |
| Overexploitation and collapse of stocks SSP (%) | Quantitative | Mean value per LME over 1950-2010 | | | | | | | | |
|  | Qualitative | <25% | | | 25-50% | | | >50% | | |
| Recent overexploitation and collapse of stocks SSP (%) | Quantitative | Mean value per LME over 1990-2010 | | | | | | | | |
|  | Qualitative | <25% | 25-50% | | | 50-75% | | | | >75% |
| Primary production required for fisheries PPR/PP (%) | Quantitative | Mean PPR/PP per LME over 1950-2010 | | | | | | | | |
|  | Qualitative | <10% | | | 10-25% | | | >25% | | |
| Recent primary production required for fisheries PPR/PP (%) | Quantitative | Mean PPR/PP per LME over 1990-2010 | | | | | | | | |
|  | Qualitative | <10% | | | 10-25% | | | >25% | | |
| PPR/PP relative to 1950 | Quantitative | Mean PPR/PP relative to 1950 over 1950-2010 | | | | | | | | |
|  | Qualitative | Decrease if <0.99 | | | Stable if  0.99 – 1.01 | | | Increase if  >1.01 | | |
| Indicator of loss of production L_index_ | Quantitative | Mean value per LME over 1950-2010 | | | | | | | | |
|  | Qualitative | <0.01 | | | 0.01-0.05 | | | >0.05 | | |
| Recent L_index_ | Quantitative | Mean value per LME over 1990-2010 | | | | | | | | |
|  | Qualitative | <0.01 | | | 0.01-0.05 | | | >0.05 | | |
| L_index_ relative to 1950 | Quantitative | Mean L_index_ relative to 1950 over 1950-2010 | | | | | | | | |
|  | Qualitative | Decrease if <0.99 | | | Stable if  0.99 – 1.01 | | | Increase if  >1.01 | | |
| Value of indicator ECI/TCI | Quantitative | Mean value ECI/TCI over 1950-2010 | | | | | | | | |
|  | Qualitative | ECI: <0.005  TCI: <2 | | | 0.005-0.01  2-3 | | | >0.01  >3 years | | |
| Values of indicator ECI_R_/TCI_R_ relative to 1950 | Quantitative | Mean ECI/TCI relative to 1950 over 1950-2010 | | | | | | | | |
|  | Qualitative | Decrease if <0.99 | | | Stable if  0.99 – 1.01 | | | Increase if  >1.01 | | |
| Fraction of fish species (%) | Quantitative | Mean Fraction per LME over 1950-2010 | | | | | | | | |
|  | Qualitative | <80% | | | 80-90% | | | >90% | | |
| Fraction of fish/shrimp/cephalopod species relative to 1950 | Quantitative | Mean value relative to 1950 over 1950-2010 | | | | | | | | |
|  | Qualitative | Decrease if <0.99 | | | Stable if  0.99 – 1.01 | | | Increase if  >1.01 | | |
| Fraction of shrimp/cephalopod species (%) | Quantitative | Mean Fraction per LME over 1950-2010 | | | | | | | | |
|  | Qualitative | <1% | | | 1-5% | | | >5% | | |
| Mean trophic level of the catch MTL | Quantitative | Mean value per LME over 1950-2010 | | | | | | | | |
|  | Qualitative | <3.40 | | | 3.40-3.60 | | | >3.60 | | |
| Mean trophic level of the catch relative to 1950 MTL | Quantitative | Mean MTL relative to 1950 value over 1950-2010 | | | | | | | | |
|  | Qualitative | Decrease if <0.99 | | | Stable if  0.99 – 1.01 | | | Increase if  >1.01 | | |
| Fishing in balance FiB | Quantitative | Mean value per LME over 1950-2010 | | | | | | | | |
|  | Qualitative | Decrease if  <-0.1 | | | Stable if  -0.1 to 0.1 | | | Increase if  >0.1 | | |
| Amount of catch (t/km²) | Quantitative | Mean value per LME over 1950-2010 | | | | | | | | |
|  | Qualitative | <0.1t/km² | 0.1-1.0 | | | 1.0-2.0 | | | | >2.0t/km² |
| Amount of catch relative to 1950 | Quantitative | Mean relative value to 1950 per LME over 1950-2010 | | | | | | | | |
|  | Qualitative | Decrease  <0.9 | Stable if  0.9 to 1.1 | | | Increase  1.1 to 4.0 | | | | Increase++  >4.0 |
| Recent amount of catch (t/km²) | Quantitative | Mean value per LME over 1990-2010 | | | | | | | | |
|  | Qualitative | <0.1t/km² | | | 0.1-1.0 | | | >1.0t/km² | | |
| Shannon diversity indicator | Quantitative | Mean value per LME over 1950-2010 | | | | | | | | |
|  | Qualitative | <2.0 | 2.0-3.0 | | | 3.0-3.5 | | | | >3.5 |
| Shannon diversity indicator relative to 1950 | Quantitative | Mean relative value to 1950 per LME over 1950-2010 | | | | | | | | |
|  | Qualitative | Decrease if  <0.9 | | | Stable if  0.9 – 1.1 | | | Increase if  >1.1 | | |
| Correlation between ECI/TCI and Shannon biodiversity indicator | Quantitative | Correlation coefficient over 1950-2010 | | | | | | | | |
|  | Qualitative | < -0.5 | -0.5 to 0 | | | 0-0.5 | | | | >0.5 |
