# Supplementary material for: Global change in the trophic functioning of marine food webs
Source: PLoS One. 2017 Aug 11;12(8):e0182826. doi: 10.1371/journal.pone.0182826 (PMC5553640; doi:10.1371/journal.pone.0182826)
Supplement: S2 Table — TCI values are given in years in the green cells and ECI values in the blue cells. LME numbers are the official numbers and correspond to the ecosystems indicated in S1 Appendix. (DOCX) [file pone.0182826.s002.docx]

S2 Table. Indicators TCI and ECI time-series per Large Marine Ecosystem. TCI values are given in years in the green cells and ECI values in the blue cells. LME numbers are the official numbers and correspond to the ecosystems indicated in S1 Appendix.

| Year  LME | 1950 | 1951 | 1952 | 1953 | 1954 | 1955 | 1956 | 1957 | 1958 | 1959 | 1960 | 1961 | 1962 | 1963 | 1964 | 1965 | 1966 | 1967 | 1968 | 1969 | 1970 |
| --- | --- | --- | --- | --- | --- | --- | --- | --- | --- | --- | --- | --- | --- | --- | --- | --- | --- | --- | --- | --- | --- |
| 1 | 2.37 | 2.35 | 2.37 | 2.37 | 2.53 | 2.56 | 2.55 | 2.54 | 2.80 | 3.52 | 4.03 | 3.82 | 3.74 | 3.37 | 3.33 | 3.44 | 3.59 | 3.72 | 3.55 | 3.58 | 3.54 |
| 2 | 2.63 | 2.61 | 2.65 | 2.58 | 2.59 | 2.67 | 2.66 | 2.65 | 2.55 | 2.69 | 2.85 | 2.77 | 2.72 | 2.71 | 2.73 | 2.96 | 2.98 | 3.01 | 2.91 | 3.10 | 2.83 |
| 3 | 3.26 | 3.21 | 3.01 | 2.73 | 2.75 | 2.72 | 2.73 | 2.68 | 2.45 | 2.39 | 2.56 | 2.51 | 2.48 | 2.45 | 2.48 | 2.54 | 2.52 | 2.45 | 2.46 | 2.56 | 2.47 |
| 4 | 2.34 | 2.33 | 1.95 | 1.90 | 1.80 | 1.81 | 1.81 | 1.80 | 1.83 | 1.82 | 1.82 | 1.82 | 1.80 | 1.80 | 1.83 | 1.84 | 1.84 | 1.86 | 1.91 | 1.91 | 1.87 |
| 5 | 1.96 | 1.99 | 1.97 | 1.96 | 1.97 | 1.95 | 1.94 | 1.94 | 1.94 | 1.93 | 1.92 | 1.89 | 2.00 | 2.13 | 2.19 | 2.10 | 2.10 | 2.03 | 2.05 | 2.10 | 2.08 |
| 6 | 1.82 | 1.88 | 1.90 | 1.83 | 1.83 | 1.84 | 1.86 | 1.84 | 1.85 | 1.84 | 1.82 | 1.86 | 1.83 | 1.85 | 1.82 | 1.85 | 1.86 | 1.84 | 1.85 | 1.80 | 1.82 |
| 7 | 2.82 | 2.90 | 2.92 | 2.90 | 2.99 | 3.00 | 2.99 | 2.95 | 3.01 | 3.05 | 2.94 | 2.94 | 2.95 | 3.02 | 3.02 | 3.04 | 3.02 | 2.96 | 2.96 | 2.88 | 2.75 |
| 8 | 4.06 | 3.97 | 3.93 | 3.92 | 3.94 | 3.94 | 3.92 | 3.98 | 4.09 | 4.08 | 4.07 | 4.05 | 4.00 | 4.06 | 4.05 | 4.03 | 4.00 | 3.90 | 3.81 | 3.68 | 3.53 |
| 9 | 4.30 | 4.28 | 4.23 | 4.23 | 4.24 | 4.23 | 4.21 | 4.25 | 4.35 | 4.37 | 4.32 | 4.27 | 4.20 | 4.26 | 4.24 | 4.23 | 4.16 | 4.01 | 3.90 | 3.76 | 3.64 |
| 10 | 2.20 | 2.25 | 2.22 | 2.25 | 2.23 | 2.20 | 2.22 | 2.19 | 2.24 | 2.27 | 2.25 | 2.20 | 2.18 | 2.21 | 2.19 | 2.16 | 2.15 | 2.15 | 2.31 | 2.28 | 2.24 |
| 11 | 2.24 | 2.26 | 2.06 | 2.01 | 1.96 | 1.95 | 1.92 | 1.94 | 1.95 | 1.93 | 1.87 | 1.88 | 1.86 | 1.88 | 1.88 | 1.90 | 1.91 | 1.90 | 1.94 | 1.98 | 1.95 |
| 12 | 2.56 | 2.55 | 2.55 | 2.31 | 2.30 | 2.27 | 2.25 | 2.24 | 2.28 | 2.33 | 2.30 | 2.21 | 2.36 | 2.31 | 2.15 | 2.23 | 2.22 | 2.25 | 2.29 | 2.15 | 2.14 |
| 13 | 3.17 | 3.13 | 3.14 | 3.13 | 3.07 | 3.16 | 3.08 | 2.97 | 2.82 | 2.89 | 2.84 | 2.89 | 2.82 | 3.68 | 3.84 | 3.06 | 3.03 | 2.97 | 2.99 | 2.94 | 3.12 |
| 14 | 3.52 | 3.38 | 3.42 | 3.31 | 3.47 | 3.59 | 3.48 | 3.66 | 3.70 | 3.74 | 3.59 | 3.60 | 3.62 | 3.69 | 4.06 | 4.01 | 4.08 | 3.84 | 3.58 | 4.06 | 3.80 |
| 15 | 3.14 | 3.14 | 3.19 | 3.13 | 3.21 | 3.14 | 3.19 | 2.99 | 3.07 | 3.24 | 3.07 | 3.13 | 3.07 | 3.19 | 3.11 | 3.06 | 3.04 | 3.12 | 2.87 | 2.80 | 2.95 |
| 16 | 2.70 | 2.70 | 2.76 | 2.69 | 2.75 | 2.72 | 2.68 | 2.68 | 2.64 | 2.69 | 2.67 | 2.69 | 2.66 | 2.68 | 2.59 | 2.61 | 2.58 | 2.54 | 2.49 | 2.46 | 2.57 |
| 17 | 2.60 | 2.60 | 2.64 | 2.45 | 2.46 | 2.36 | 2.40 | 2.43 | 2.38 | 2.48 | 2.46 | 2.30 | 2.49 | 2.47 | 2.28 | 2.42 | 2.34 | 2.36 | 2.38 | 2.31 | 2.36 |
| 18 | 4.22 | 4.23 | 4.26 | 4.16 | 4.42 | 4.40 | 4.37 | 4.34 | 4.48 | 4.43 | 4.16 | 4.09 | 4.03 | 4.13 | 4.15 | 4.16 | 4.03 | 3.94 | 3.79 | 3.70 | 3.47 |
| 19 | 6.12 | 5.72 | 5.65 | 5.33 | 5.74 | 4.81 | 4.58 | 5.15 | 5.13 | 4.84 | 5.21 | 5.15 | 4.98 | 5.12 | 4.94 | 4.88 | 5.35 | 5.41 | 5.40 | 5.45 | 5.51 |
| 20 | 6.25 | 5.72 | 6.18 | 6.86 | 6.14 | 6.72 | 6.69 | 6.41 | 5.10 | 4.99 | 4.84 | 4.78 | 4.89 | 4.77 | 5.09 | 4.60 | 4.53 | 4.49 | 4.66 | 5.12 | 4.94 |
| 21 | 5.75 | 5.38 | 5.39 | 5.86 | 5.19 | 5.35 | 4.59 | 4.58 | 4.55 | 4.50 | 4.61 | 4.39 | 4.80 | 4.74 | 4.76 | 4.45 | 4.31 | 4.38 | 4.69 | 4.55 | 4.39 |
| 22 | 2.58 | 2.60 | 2.59 | 2.52 | 2.54 | 2.47 | 2.52 | 2.58 | 2.65 | 2.70 | 2.64 | 2.65 | 2.67 | 2.63 | 2.73 | 2.72 | 2.62 | 2.66 | 2.67 | 2.71 | 2.75 |
| 23 | 3.38 | 3.40 | 3.31 | 3.17 | 3.26 | 3.27 | 3.27 | 3.24 | 3.18 | 3.18 | 3.21 | 3.23 | 3.39 | 3.38 | 3.32 | 3.16 | 3.35 | 3.42 | 3.37 | 3.32 | 3.31 |
| 24 | 2.87 | 2.87 | 2.85 | 2.83 | 2.76 | 2.77 | 2.78 | 2.77 | 2.74 | 2.81 | 2.90 | 2.87 | 2.93 | 2.98 | 2.91 | 2.92 | 2.89 | 2.88 | 2.89 | 2.83 | 2.79 |
| 25 | 2.90 | 2.94 | 2.84 | 2.85 | 2.74 | 2.81 | 2.85 | 2.90 | 2.79 | 2.77 | 2.75 | 2.89 | 2.78 | 2.81 | 2.60 | 2.39 | 2.57 | 2.55 | 2.56 | 2.70 | 2.74 |
| 26 | 2.05 | 2.04 | 2.07 | 2.08 | 2.07 | 2.04 | 2.05 | 2.02 | 2.00 | 2.01 | 2.09 | 2.08 | 2.11 | 2.12 | 2.10 | 2.08 | 2.07 | 2.10 | 2.12 | 2.13 | 2.06 |
| 27 | 2.58 | 2.60 | 2.54 | 2.50 | 2.53 | 2.61 | 2.56 | 2.54 | 2.57 | 2.68 | 2.68 | 2.69 | 2.69 | 2.68 | 2.84 | 2.67 | 2.76 | 2.77 | 2.86 | 2.86 | 2.99 |
| 28 | 2.18 | 2.17 | 2.19 | 2.17 | 2.18 | 2.17 | 2.16 | 2.16 | 2.12 | 2.10 | 2.05 | 2.05 | 2.09 | 2.03 | 2.04 | 1.99 | 1.97 | 1.97 | 1.95 | 1.92 | 1.94 |
| 29 | 3.87 | 3.92 | 3.95 | 3.96 | 4.00 | 3.74 | 3.78 | 3.91 | 3.77 | 3.60 | 3.49 | 3.50 | 3.51 | 3.53 | 3.59 | 3.63 | 3.58 | 3.54 | 3.56 | 3.51 | 3.42 |
| 30 | 2.49 | 2.52 | 2.56 | 2.57 | 2.55 | 2.58 | 2.55 | 2.56 | 2.56 | 2.57 | 2.58 | 2.58 | 2.57 | 2.58 | 2.57 | 2.70 | 2.65 | 2.49 | 2.52 | 2.53 | 2.34 |
| 31 | 2.23 | 2.23 | 2.23 | 2.19 | 2.18 | 2.17 | 2.18 | 2.19 | 2.19 | 2.20 | 2.19 | 2.16 | 2.20 | 2.15 | 2.18 | 2.17 | 2.19 | 2.19 | 2.18 | 2.16 | 2.14 |
| 32 | 1.57 | 1.57 | 1.58 | 1.57 | 1.50 | 1.54 | 1.48 | 1.52 | 1.59 | 1.61 | 1.61 | 1.62 | 1.60 | 1.60 | 1.60 | 1.62 | 1.61 | 1.60 | 1.60 | 1.59 | 1.59 |
| 33 | 2.74 | 2.74 | 2.74 | 2.73 | 2.73 | 2.72 | 2.77 | 2.72 | 2.74 | 2.78 | 2.82 | 2.74 | 2.71 | 2.69 | 2.64 | 2.67 | 2.72 | 2.73 | 2.79 | 2.78 | 2.73 |
| 34 | 1.53 | 1.54 | 1.53 | 1.52 | 1.53 | 1.56 | 1.51 | 1.52 | 1.53 | 1.55 | 1.55 | 1.54 | 1.54 | 1.53 | 1.48 | 1.49 | 1.47 | 1.44 | 1.45 | 1.46 | 1.44 |
| 35 | 1.37 | 1.38 | 1.39 | 1.39 | 1.36 | 1.35 | 1.36 | 1.37 | 1.37 | 1.38 | 1.37 | 1.39 | 1.41 | 1.43 | 1.45 | 1.48 | 1.48 | 1.48 | 1.48 | 1.48 | 1.28 |
| 36 | 1.37 | 1.39 | 1.42 | 1.43 | 1.32 | 1.33 | 1.35 | 1.36 | 1.35 | 1.35 | 1.36 | 1.36 | 1.37 | 1.37 | 1.38 | 1.40 | 1.40 | 1.40 | 1.39 | 1.40 | 1.35 |
| 37 | 1.27 | 1.27 | 1.27 | 1.27 | 1.27 | 1.27 | 1.27 | 1.27 | 1.27 | 1.27 | 1.27 | 1.27 | 1.27 | 1.28 | 1.28 | 1.28 | 1.28 | 1.28 | 1.29 | 1.28 | 1.30 |
| 38 | 1.21 | 1.21 | 1.21 | 1.21 | 1.21 | 1.21 | 1.21 | 1.21 | 1.21 | 1.20 | 1.21 | 1.21 | 1.21 | 1.21 | 1.21 | 1.21 | 1.21 | 1.21 | 1.22 | 1.22 | 1.25 |
| 39 | 2.63 | 2.58 | 2.49 | 2.42 | 2.38 | 2.28 | 2.28 | 2.25 | 2.23 | 2.21 | 2.16 | 2.53 | 2.52 | 2.52 | 2.41 | 2.42 | 2.41 | 2.30 | 2.20 | 2.16 | 2.02 |
| 40 | 2.33 | 2.30 | 2.25 | 2.22 | 2.21 | 2.17 | 2.15 | 2.13 | 2.13 | 2.10 | 2.09 | 2.18 | 2.18 | 2.17 | 2.14 | 2.15 | 2.11 | 2.06 | 2.03 | 2.00 | 1.93 |
| 41 | 2.72 | 2.66 | 2.65 | 2.62 | 2.56 | 2.52 | 2.50 | 2.51 | 2.50 | 2.51 | 2.47 | 2.43 | 2.43 | 2.45 | 2.39 | 2.39 | 2.42 | 2.38 | 2.36 | 2.34 | 2.28 |
| 42 | 3.14 | 3.12 | 3.12 | 3.14 | 3.11 | 3.10 | 3.14 | 3.20 | 3.14 | 3.33 | 3.32 | 3.30 | 3.18 | 3.24 | 3.19 | 3.28 | 3.30 | 3.27 | 3.25 | 3.07 | 3.08 |
| 43 | 2.97 | 2.97 | 2.99 | 3.01 | 3.01 | 3.00 | 3.05 | 3.09 | 3.08 | 3.19 | 3.21 | 3.25 | 3.26 | 3.30 | 3.34 | 3.20 | 3.17 | 2.90 | 2.93 | 2.94 | 2.67 |
| 44 | 2.83 | 2.82 | 2.82 | 2.81 | 2.81 | 2.81 | 2.81 | 2.81 | 2.81 | 2.83 | 2.83 | 2.84 | 2.85 | 2.87 | 2.86 | 2.88 | 2.83 | 2.82 | 2.68 | 2.58 | 2.52 |
| 45 | 2.56 | 2.53 | 2.53 | 2.53 | 2.51 | 2.51 | 2.51 | 2.51 | 2.50 | 2.52 | 2.51 | 2.49 | 2.49 | 2.50 | 2.49 | 2.52 | 2.46 | 2.42 | 2.33 | 2.22 | 2.15 |
| 46 | 3.47 | 3.48 | 3.44 | 3.48 | 3.48 | 3.46 | 3.43 | 3.45 | 3.46 | 3.46 | 3.47 | 3.46 | 3.48 | 3.48 | 3.41 | 3.42 | 3.40 | 3.41 | 3.41 | 3.44 | 3.42 |
| 49 | 1.96 | 1.97 | 1.97 | 1.96 | 1.87 | 1.93 | 1.89 | 1.88 | 1.87 | 1.88 | 1.95 | 1.99 | 1.92 | 1.86 | 1.87 | 1.79 | 1.79 | 1.80 | 1.78 | 1.81 | 1.84 |
| 50 | 2.02 | 2.02 | 2.02 | 2.03 | 2.00 | 2.03 | 1.98 | 1.97 | 1.96 | 1.98 | 2.01 | 2.05 | 2.00 | 1.99 | 1.97 | 1.92 | 1.90 | 1.92 | 1.90 | 1.92 | 1.95 |
| 51 | 2.63 | 2.62 | 2.63 | 2.64 | 2.60 | 2.60 | 2.59 | 2.61 | 2.57 | 2.58 | 2.61 | 2.65 | 2.56 | 2.56 | 2.56 | 2.47 | 2.45 | 2.46 | 2.46 | 2.50 | 2.54 |
| 52 | 3.08 | 3.02 | 3.05 | 3.13 | 3.13 | 3.15 | 3.13 | 3.18 | 3.29 | 3.25 | 3.28 | 3.28 | 3.22 | 3.26 | 3.35 | 3.38 | 3.44 | 3.47 | 3.51 | 3.47 | 3.57 |
| 53 | 2.91 | 2.87 | 2.89 | 2.95 | 2.99 | 3.06 | 3.05 | 3.01 | 3.37 | 3.32 | 3.26 | 3.18 | 2.93 | 2.83 | 3.12 | 2.95 | 2.94 | 2.88 | 3.00 | 2.96 | 3.09 |
| 59 | 4.18 | 4.14 | 4.15 | 3.99 | 3.93 | 3.79 | 3.85 | 3.94 | 3.90 | 3.77 | 3.68 | 3.59 | 3.60 | 3.63 | 3.57 | 3.54 | 3.59 | 3.64 | 3.74 | 3.78 | 3.76 |
| 60 | 3.20 | 3.20 | 3.01 | 3.21 | 2.97 | 2.99 | 3.17 | 3.22 | 3.27 | 3.21 | 3.23 | 3.20 | 3.25 | 3.14 | 3.05 | 2.96 | 2.98 | 3.01 | 3.06 | 3.10 | 3.06 |
| 62 | 4.61 | 4.61 | 4.61 | 4.70 | 4.49 | 4.32 | 4.17 | 4.11 | 4.08 | 4.07 | 4.06 | 4.07 | 3.96 | 4.13 | 4.05 | 4.06 | 4.12 | 4.13 | 4.09 | 3.99 | 4.00 |
| 65 | 3.35 | 3.44 | 3.39 | 3.58 | 4.04 | 3.88 | 3.87 | 4.07 | 4.82 | 6.05 | 6.99 | 6.73 | 6.77 | 5.54 | 5.37 | 4.20 | 4.95 | 5.85 | 5.47 | 5.64 | 5.15 |
| 66 | 4.16 | 4.07 | 4.22 | 4.08 | 4.41 | 4.32 | 4.39 | 4.31 | 4.37 | 4.19 | 3.87 | 3.89 | 3.93 | 4.07 | 4.11 | 4.12 | 3.85 | 3.99 | 3.65 | 3.58 | 3.15 |
| Year  LME | 1971 | 1972 | 1973 | 1974 | 1975 | 1976 | 1977 | 1978 | 1979 | 1980 | 1981 | 1982 | 1983 | 1984 | 1985 | 1986 | 1987 | 1988 | 1989 | 1990 |  |
| 1 | 3.53 | 3.72 | 3.68 | 3.69 | 3.69 | 3.60 | 3.51 | 3.52 | 3.48 | 3.48 | 3.51 | 3.53 | 3.60 | 3.74 | 3.78 | 3.71 | 3.61 | 3.67 | 3.65 | 3.59 |  |
| 2 | 2.84 | 2.76 | 2.60 | 2.66 | 2.88 | 2.70 | 2.50 | 2.44 | 2.58 | 2.62 | 2.62 | 2.61 | 2.81 | 2.94 | 2.78 | 2.93 | 3.17 | 2.76 | 2.73 | 2.78 |  |
| 3 | 2.44 | 2.45 | 2.54 | 2.21 | 2.30 | 2.33 | 2.19 | 2.19 | 2.42 | 2.36 | 2.38 | 2.35 | 2.52 | 2.59 | 2.30 | 2.11 | 2.19 | 1.93 | 1.89 | 2.06 |  |
| 4 | 1.92 | 1.83 | 1.87 | 1.80 | 1.80 | 1.79 | 1.72 | 1.77 | 1.80 | 1.76 | 1.81 | 1.77 | 1.90 | 1.89 | 1.87 | 1.92 | 1.84 | 1.88 | 1.88 | 2.05 |  |
| 5 | 2.07 | 1.99 | 1.99 | 1.89 | 1.97 | 1.98 | 1.77 | 2.19 | 2.10 | 2.16 | 2.13 | 2.18 | 2.15 | 2.20 | 2.12 | 2.02 | 2.06 | 2.08 | 2.08 | 2.17 |  |
| 6 | 1.79 | 1.83 | 1.85 | 1.87 | 1.90 | 1.92 | 1.93 | 1.85 | 1.74 | 1.81 | 1.72 | 1.73 | 1.71 | 1.73 | 1.77 | 1.79 | 1.75 | 1.77 | 1.73 | 1.70 |  |
| 7 | 2.78 | 2.75 | 2.73 | 2.62 | 2.60 | 2.56 | 2.58 | 2.64 | 2.53 | 2.58 | 2.56 | 2.64 | 2.58 | 2.72 | 2.61 | 2.58 | 2.62 | 2.57 | 2.45 | 2.46 |  |
| 8 | 3.47 | 3.40 | 3.28 | 3.18 | 3.02 | 2.72 | 2.53 | 2.43 | 2.26 | 2.42 | 2.41 | 2.53 | 2.74 | 2.84 | 2.80 | 2.88 | 2.94 | 2.88 | 2.73 | 2.74 |  |
| 9 | 3.61 | 3.55 | 3.44 | 3.34 | 3.17 | 2.88 | 2.69 | 2.65 | 2.43 | 2.61 | 2.73 | 2.82 | 2.94 | 3.02 | 3.00 | 3.04 | 3.05 | 3.00 | 2.89 | 2.89 |  |
| 10 | 2.21 | 2.21 | 2.43 | 2.23 | 2.28 | 2.19 | 2.21 | 2.32 | 2.34 | 2.32 | 2.37 | 2.30 | 1.96 | 1.93 | 2.11 | 2.10 | 2.10 | 2.08 | 1.92 | 1.93 |  |
| 11 | 1.95 | 1.98 | 2.01 | 2.01 | 2.04 | 2.08 | 2.17 | 2.15 | 2.16 | 2.15 | 2.11 | 2.16 | 2.13 | 2.17 | 2.17 | 2.10 | 2.08 | 2.17 | 2.09 | 2.09 |  |
| 12 | 2.06 | 2.11 | 2.06 | 2.06 | 2.02 | 2.06 | 2.03 | 2.11 | 2.14 | 2.14 | 2.18 | 2.10 | 2.13 | 2.13 | 2.14 | 2.05 | 1.98 | 1.95 | 2.03 | 2.14 |  |
| 13 | 3.16 | 3.15 | 3.34 | 3.38 | 3.44 | 3.29 | 3.82 | 3.80 | 3.69 | 3.76 | 3.83 | 3.91 | 4.29 | 4.17 | 4.12 | 3.80 | 3.89 | 4.16 | 4.07 | 3.87 |  |
| 14 | 3.71 | 3.93 | 3.58 | 3.54 | 3.42 | 3.11 | 3.98 | 2.08 | 2.13 | 3.44 | 3.26 | 2.40 | 2.43 | 2.30 | 2.18 | 2.03 | 1.83 | 1.84 | 1.70 | 1.74 |  |
| 15 | 2.92 | 2.94 | 3.18 | 3.04 | 3.04 | 3.09 | 3.09 | 3.01 | 2.93 | 2.90 | 2.91 | 2.73 | 2.90 | 2.78 | 2.49 | 2.50 | 2.39 | 2.24 | 2.25 | 2.22 |  |
| 16 | 2.53 | 2.55 | 2.61 | 2.62 | 2.58 | 2.59 | 2.59 | 2.67 | 2.75 | 2.58 | 2.55 | 2.46 | 2.49 | 2.45 | 2.44 | 2.46 | 2.44 | 2.34 | 2.30 | 2.27 |  |
| 17 | 2.35 | 2.33 | 2.35 | 2.37 | 2.35 | 2.42 | 2.40 | 2.44 | 2.45 | 2.43 | 2.41 | 2.38 | 2.41 | 2.39 | 2.40 | 2.40 | 2.38 | 2.33 | 2.35 | 2.28 |  |
| 18 | 3.42 | 3.34 | 3.21 | 3.02 | 2.77 | 2.54 | 2.62 | 2.59 | 2.60 | 2.47 | 2.42 | 2.43 | 2.47 | 2.52 | 2.54 | 2.61 | 2.71 | 2.62 | 2.53 | 2.68 |  |
| 19 | 5.60 | 5.72 | 5.11 | 5.73 | 5.47 | 5.56 | 5.68 | 5.46 | 5.44 | 5.58 | 5.97 | 5.73 | 5.35 | 5.18 | 4.56 | 4.67 | 4.59 | 4.59 | 4.56 | 4.51 |  |
| 20 | 5.23 | 5.05 | 4.81 | 4.43 | 4.79 | 4.60 | 4.57 | 4.02 | 4.35 | 4.41 | 4.40 | 4.31 | 4.10 | 3.90 | 4.13 | 4.55 | 4.75 | 4.41 | 4.37 | 4.21 |  |
| 21 | 4.19 | 4.17 | 4.63 | 4.55 | 4.64 | 4.39 | 4.31 | 4.47 | 4.40 | 4.36 | 4.21 | 4.35 | 4.08 | 4.08 | 4.16 | 4.52 | 4.65 | 4.44 | 4.36 | 4.35 |  |
| 22 | 2.85 | 2.85 | 2.79 | 2.76 | 2.80 | 2.79 | 2.81 | 2.79 | 2.78 | 2.83 | 2.84 | 2.86 | 2.89 | 2.90 | 2.85 | 2.88 | 2.83 | 2.89 | 2.89 | 2.94 |  |
| 23 | 3.24 | 3.21 | 3.21 | 3.30 | 3.27 | 3.36 | 3.31 | 3.49 | 3.58 | 3.74 | 3.87 | 3.98 | 4.11 | 4.05 | 3.86 | 3.35 | 3.28 | 3.28 | 3.65 | 3.50 |  |
| 24 | 2.63 | 2.55 | 2.61 | 2.58 | 2.51 | 2.58 | 2.41 | 2.37 | 2.35 | 2.42 | 2.40 | 2.37 | 2.38 | 2.36 | 2.39 | 2.44 | 2.45 | 2.43 | 2.46 | 2.36 |  |
| 25 | 2.97 | 2.67 | 2.65 | 2.71 | 2.73 | 3.12 | 3.21 | 2.94 | 3.08 | 2.83 | 2.53 | 2.72 | 2.83 | 2.86 | 2.81 | 2.90 | 2.74 | 2.78 | 2.73 | 2.75 |  |
| 26 | 2.06 | 2.00 | 2.01 | 2.03 | 2.02 | 2.03 | 2.02 | 2.04 | 1.99 | 1.91 | 1.96 | 1.96 | 1.97 | 2.02 | 2.01 | 2.01 | 2.02 | 1.98 | 2.00 | 2.04 |  |
| 27 | 3.03 | 2.97 | 2.93 | 2.86 | 2.94 | 2.89 | 2.99 | 3.02 | 3.06 | 3.04 | 2.80 | 2.85 | 2.76 | 2.86 | 2.89 | 2.79 | 2.71 | 2.70 | 2.65 | 2.57 |  |
| 28 | 1.95 | 1.95 | 1.93 | 1.89 | 1.91 | 1.89 | 1.88 | 1.84 | 1.85 | 1.87 | 1.88 | 1.93 | 1.93 | 1.96 | 2.03 | 2.07 | 2.04 | 2.06 | 2.11 | 2.03 |  |
| 29 | 3.54 | 3.57 | 3.40 | 3.43 | 3.64 | 3.76 | 3.62 | 3.42 | 3.48 | 3.62 | 3.73 | 3.83 | 3.72 | 3.90 | 3.73 | 3.82 | 3.59 | 3.69 | 3.59 | 3.72 |  |
| 30 | 2.25 | 2.25 | 2.23 | 2.20 | 2.22 | 2.21 | 2.11 | 2.05 | 2.05 | 1.97 | 1.99 | 2.00 | 2.01 | 2.03 | 2.02 | 1.97 | 1.95 | 1.96 | 1.96 | 1.92 |  |
| 31 | 2.14 | 2.14 | 2.15 | 2.22 | 2.23 | 2.13 | 2.18 | 2.18 | 2.16 | 2.19 | 2.14 | 2.02 | 2.11 | 2.10 | 2.05 | 2.04 | 1.97 | 2.01 | 2.01 | 2.01 |  |
| 32 | 1.58 | 1.57 | 1.54 | 1.59 | 1.56 | 1.57 | 1.60 | 1.59 | 1.60 | 1.59 | 1.61 | 1.61 | 1.59 | 1.57 | 1.57 | 1.56 | 1.59 | 1.60 | 1.57 | 1.57 |  |
| 33 | 2.72 | 2.68 | 2.81 | 2.79 | 2.68 | 2.67 | 2.71 | 2.77 | 2.86 | 2.79 | 2.70 | 2.79 | 2.68 | 2.54 | 2.44 | 2.47 | 2.53 | 2.50 | 2.54 | 2.47 |  |
| 34 | 1.39 | 1.39 | 1.35 | 1.37 | 1.35 | 1.33 | 1.37 | 1.35 | 1.35 | 1.38 | 1.39 | 1.37 | 1.36 | 1.36 | 1.41 | 1.42 | 1.40 | 1.39 | 1.37 | 1.35 |  |
| 35 | 1.28 | 1.26 | 1.32 | 1.25 | 1.25 | 1.30 | 1.36 | 1.33 | 1.33 | 1.29 | 1.31 | 1.28 | 1.31 | 1.24 | 1.25 | 1.31 | 1.30 | 1.30 | 1.28 | 1.27 |  |
| 36 | 1.34 | 1.32 | 1.37 | 1.33 | 1.33 | 1.34 | 1.38 | 1.38 | 1.37 | 1.36 | 1.37 | 1.35 | 1.35 | 1.34 | 1.34 | 1.37 | 1.38 | 1.35 | 1.35 | 1.36 |  |
| 37 | 1.29 | 1.29 | 1.31 | 1.30 | 1.29 | 1.28 | 1.28 | 1.28 | 1.28 | 1.28 | 1.28 | 1.30 | 1.30 | 1.30 | 1.30 | 1.29 | 1.29 | 1.29 | 1.29 | 1.27 |  |
| 38 | 1.25 | 1.25 | 1.26 | 1.26 | 1.26 | 1.25 | 1.26 | 1.25 | 1.25 | 1.25 | 1.26 | 1.28 | 1.28 | 1.27 | 1.28 | 1.29 | 1.28 | 1.27 | 1.27 | 1.24 |  |
| 39 | 1.87 | 1.89 | 1.87 | 1.79 | 1.76 | 1.67 | 1.65 | 1.71 | 1.61 | 1.57 | 1.65 | 1.68 | 1.66 | 1.64 | 1.59 | 1.64 | 1.62 | 1.61 | 1.62 | 1.62 |  |
| 40 | 1.83 | 1.83 | 1.82 | 1.75 | 1.81 | 1.77 | 1.74 | 1.77 | 1.70 | 1.74 | 1.71 | 1.74 | 1.74 | 1.73 | 1.48 | 1.55 | 1.49 | 1.44 | 1.48 | 1.46 |  |
| 41 | 2.27 | 2.26 | 2.25 | 2.23 | 2.27 | 2.25 | 2.26 | 2.25 | 2.19 | 2.20 | 2.19 | 2.18 | 2.17 | 2.19 | 2.08 | 2.15 | 2.11 | 2.11 | 2.20 | 2.23 |  |
| 42 | 2.93 | 2.95 | 2.82 | 2.73 | 2.84 | 2.76 | 2.86 | 2.92 | 2.78 | 2.83 | 2.91 | 2.78 | 2.67 | 2.66 | 2.72 | 2.88 | 2.93 | 2.93 | 2.90 | 2.89 |  |
| 43 | 2.56 | 2.49 | 2.50 | 2.40 | 2.46 | 2.70 | 2.71 | 2.68 | 2.67 | 2.73 | 2.74 | 2.92 | 2.90 | 2.89 | 2.89 | 2.97 | 2.95 | 2.97 | 2.84 | 2.87 |  |
| 44 | 2.44 | 2.34 | 2.32 | 2.25 | 2.30 | 2.31 | 2.31 | 2.34 | 2.38 | 2.41 | 2.44 | 2.47 | 2.47 | 2.49 | 2.48 | 2.62 | 2.64 | 2.62 | 2.52 | 2.54 |  |
| 45 | 2.05 | 2.03 | 2.02 | 2.22 | 1.96 | 1.94 | 1.94 | 1.97 | 2.00 | 2.03 | 2.06 | 2.09 | 2.08 | 2.09 | 2.01 | 2.13 | 2.13 | 2.18 | 2.15 | 2.18 |  |
| 46 | 3.46 | 3.47 | 3.33 | 3.38 | 3.45 | 3.43 | 3.31 | 3.18 | 3.40 | 3.53 | 3.45 | 3.44 | 3.18 | 2.98 | 3.23 | 3.34 | 3.15 | 3.18 | 3.02 | 2.77 |  |
| 49 | 1.89 | 1.86 | 1.94 | 1.95 | 1.97 | 2.01 | 1.99 | 2.02 | 2.04 | 2.02 | 2.06 | 2.01 | 2.01 | 2.03 | 2.01 | 2.01 | 1.98 | 2.00 | 2.00 | 2.03 |  |
| 50 | 2.03 | 2.00 | 2.09 | 2.10 | 2.14 | 2.18 | 2.15 | 2.14 | 2.17 | 2.16 | 2.20 | 2.20 | 2.22 | 2.26 | 2.27 | 2.27 | 2.23 | 2.26 | 2.24 | 2.27 |  |
| 51 | 2.63 | 2.61 | 2.72 | 2.76 | 2.79 | 2.87 | 2.88 | 2.94 | 3.00 | 2.96 | 3.05 | 3.05 | 3.04 | 3.07 | 3.05 | 3.05 | 2.97 | 3.02 | 2.95 | 3.01 |  |
| 52 | 3.66 | 3.56 | 3.77 | 3.85 | 3.94 | 4.07 | 4.19 | 4.32 | 4.38 | 4.39 | 4.51 | 4.55 | 4.64 | 4.57 | 4.44 | 4.49 | 4.47 | 4.51 | 4.39 | 4.45 |  |
| 53 | 3.01 | 3.00 | 3.11 | 3.05 | 3.02 | 3.10 | 3.14 | 3.33 | 3.34 | 3.41 | 3.54 | 3.52 | 3.69 | 3.56 | 3.34 | 3.37 | 3.38 | 3.37 | 3.16 | 3.14 |  |
| 59 | 3.74 | 3.80 | 3.60 | 3.71 | 3.62 | 3.64 | 3.64 | 3.59 | 3.61 | 3.65 | 3.67 | 3.54 | 3.54 | 3.54 | 3.60 | 3.44 | 3.47 | 3.54 | 3.57 | 3.52 |  |
| 60 | 3.10 | 3.13 | 3.22 | 3.49 | 3.35 | 3.32 | 3.44 | 3.45 | 3.48 | 3.55 | 3.51 | 3.44 | 3.37 | 3.20 | 3.13 | 3.12 | 3.09 | 3.08 | 3.08 | 3.08 |  |
| 62 | 3.90 | 3.73 | 3.54 | 3.63 | 3.76 | 3.45 | 3.49 | 3.39 | 3.36 | 3.28 | 3.19 | 3.37 | 3.42 | 3.27 | 3.27 | 3.14 | 3.37 | 3.55 | 3.66 | 3.60 |  |
| 65 | 5.15 | 5.78 | 5.75 | 5.65 | 5.56 | 5.15 | 4.58 | 4.52 | 4.16 | 4.27 | 3.76 | 3.85 | 3.97 | 4.44 | 5.22 | 5.39 | 5.21 | 5.83 | 5.24 | 4.77 |  |
| 66 | 3.12 | 2.99 | 2.79 | 2.71 | 2.43 | 2.24 | 2.70 | 2.70 | 2.78 | 2.69 | 2.60 | 2.57 | 2.67 | 2.60 | 2.57 | 2.61 | 2.74 | 2.77 | 2.82 | 2.77 |  |
| Year  LME | 1991 | 1992 | 1993 | 1994 | 1995 | 1996 | 1997 | 1998 | 1999 | 2000 | 2001 | 2002 | 2003 | 2004 | 2005 | 2006 | 2007 | 2008 | 2009 | 2010 |  |
| 1 | 3.62 | 3.66 | 3.61 | 3.60 | 3.55 | 3.50 | 3.61 | 3.53 | 3.49 | 3.57 | 3.53 | 3.60 | 3.55 | 3.54 | 3.47 | 3.50 | 3.48 | 3.47 | 3.45 | 3.42 |  |
| 2 | 2.91 | 2.90 | 2.83 | 2.92 | 3.03 | 3.12 | 3.12 | 3.28 | 3.30 | 3.31 | 3.22 | 3.12 | 3.23 | 3.30 | 3.34 | 3.32 | 3.32 | 3.32 | 3.56 | 3.42 |  |
| 3 | 2.12 | 2.12 | 1.90 | 1.89 | 1.89 | 1.81 | 1.84 | 2.55 | 1.76 | 1.73 | 1.71 | 1.66 | 1.78 | 1.84 | 1.79 | 1.84 | 1.79 | 1.80 | 1.69 | 1.60 |  |
| 4 | 2.01 | 1.97 | 1.95 | 2.01 | 1.91 | 1.84 | 1.89 | 2.05 | 1.91 | 1.94 | 1.92 | 1.87 | 1.84 | 1.84 | 1.94 | 1.91 | 1.93 | 1.93 | 1.95 | 2.00 |  |
| 5 | 2.17 | 2.10 | 2.08 | 2.12 | 2.03 | 1.96 | 2.11 | 1.89 | 1.88 | 1.85 | 1.85 | 1.86 | 1.79 | 1.79 | 1.87 | 1.76 | 1.80 | 1.87 | 1.75 | 1.80 |  |
| 6 | 1.73 | 1.71 | 1.70 | 1.65 | 1.66 | 1.59 | 1.68 | 1.69 | 1.67 | 1.67 | 1.70 | 1.73 | 1.72 | 1.68 | 1.74 | 1.70 | 1.77 | 1.74 | 1.70 | 1.73 |  |
| 7 | 2.48 | 2.43 | 2.36 | 2.29 | 2.28 | 2.25 | 2.22 | 2.34 | 2.35 | 2.22 | 2.38 | 2.26 | 2.34 | 2.16 | 2.22 | 2.21 | 2.23 | 2.23 | 2.22 | 2.12 |  |
| 8 | 2.68 | 2.61 | 2.44 | 2.22 | 2.16 | 2.09 | 2.17 | 2.25 | 2.20 | 2.17 | 2.13 | 2.13 | 2.12 | 2.01 | 2.05 | 1.88 | 1.98 | 1.92 | 2.10 | 2.09 |  |
| 9 | 2.81 | 2.73 | 2.55 | 2.24 | 2.27 | 2.23 | 2.23 | 2.23 | 2.33 | 2.31 | 2.33 | 2.32 | 2.33 | 2.09 | 2.18 | 2.07 | 2.16 | 2.10 | 2.06 | 2.12 |  |
| 10 | 2.10 | 2.07 | 2.08 | 2.03 | 2.03 | 2.07 | 2.09 | 2.00 | 2.07 | 2.14 | 2.13 | 2.21 | 2.43 | 2.49 | 2.35 | 2.27 | 2.25 | 2.22 | 2.13 | 2.00 |  |
| 11 | 2.12 | 2.05 | 2.05 | 2.06 | 2.14 | 1.97 | 1.95 | 2.24 | 2.00 | 2.02 | 2.17 | 1.90 | 1.89 | 1.88 | 1.99 | 1.92 | 1.91 | 1.80 | 1.83 | 1.90 |  |
| 12 | 2.16 | 2.13 | 2.11 | 2.08 | 2.08 | 2.07 | 2.00 | 2.00 | 2.01 | 2.05 | 2.01 | 1.98 | 1.94 | 1.99 | 2.03 | 1.92 | 1.95 | 2.01 | 1.96 | 1.99 |  |
| 13 | 3.56 | 3.36 | 3.12 | 3.13 | 3.24 | 3.72 | 3.58 | 2.95 | 2.94 | 2.87 | 2.90 | 2.59 | 2.52 | 2.38 | 2.38 | 2.40 | 2.41 | 2.27 | 2.22 | 2.14 |  |
| 14 | 1.76 | 1.57 | 1.80 | 1.79 | 2.07 | 2.01 | 2.20 | 2.16 | 1.68 | 1.98 | 1.89 | 1.95 | 2.08 | 2.75 | 2.59 | 2.42 | 2.10 | 2.30 | 2.84 | 2.83 |  |
| 15 | 2.19 | 2.13 | 2.72 | 2.65 | 2.55 | 2.62 | 2.45 | 2.64 | 2.66 | 2.53 | 2.83 | 2.68 | 2.65 | 2.71 | 2.73 | 2.68 | 2.59 | 2.60 | 2.70 | 2.84 |  |
| 16 | 2.22 | 2.29 | 2.59 | 2.55 | 2.53 | 2.56 | 2.53 | 2.53 | 2.56 | 2.63 | 2.66 | 2.66 | 2.62 | 2.56 | 2.54 | 2.57 | 2.54 | 2.53 | 2.56 | 2.57 |  |
| 17 | 2.29 | 2.31 | 2.43 | 2.35 | 2.32 | 2.31 | 2.27 | 2.22 | 2.32 | 2.28 | 2.28 | 2.26 | 2.21 | 2.20 | 2.23 | 2.27 | 2.25 | 2.28 | 2.26 | 2.30 |  |
| 18 | 2.79 | 2.79 | 3.02 | 3.13 | 2.91 | 2.83 | 2.85 | 2.88 | 2.95 | 3.00 | 2.99 | 2.98 | 3.09 | 2.98 | 2.99 | 2.89 | 2.69 | 2.59 | 2.74 | 2.82 |  |
| 19 | 4.68 | 4.46 | 4.37 | 4.30 | 4.41 | 4.43 | 4.38 | 4.61 | 5.06 | 4.91 | 4.45 | 4.46 | 4.31 | 4.43 | 4.49 | 4.47 | 4.31 | 4.28 | 4.02 | 4.68 |  |
| 20 | 3.96 | 4.15 | 4.03 | 4.22 | 4.51 | 4.28 | 4.47 | 4.26 | 4.08 | 4.00 | 4.20 | 4.12 | 4.28 | 4.35 | 4.43 | 4.26 | 4.17 | 4.02 | 4.00 | 4.03 |  |
| 21 | 4.02 | 3.98 | 3.95 | 4.26 | 4.36 | 4.40 | 4.38 | 4.34 | 4.34 | 4.15 | 3.97 | 4.12 | 3.86 | 4.07 | 4.23 | 4.30 | 4.33 | 4.42 | 4.29 | 4.09 |  |
| 22 | 2.84 | 2.87 | 2.90 | 2.80 | 2.81 | 2.78 | 2.79 | 2.78 | 2.80 | 2.75 | 2.75 | 2.76 | 2.75 | 2.73 | 2.74 | 2.72 | 2.64 | 2.60 | 2.69 | 2.61 |  |
| 23 | 3.36 | 3.28 | 3.17 | 3.13 | 3.18 | 3.20 | 3.17 | 3.15 | 3.18 | 3.18 | 3.21 | 3.20 | 3.21 | 3.21 | 3.18 | 3.22 | 3.19 | 3.18 | 3.18 | 3.20 |  |
| 24 | 2.42 | 2.41 | 2.37 | 2.39 | 2.48 | 2.49 | 2.47 | 2.43 | 2.40 | 2.37 | 2.42 | 2.35 | 2.33 | 2.31 | 2.49 | 2.48 | 2.45 | 2.49 | 2.63 | 2.63 |  |
| 25 | 2.75 | 2.61 | 2.75 | 2.70 | 2.72 | 2.61 | 2.52 | 2.59 | 2.52 | 2.51 | 2.52 | 2.53 | 2.49 | 2.70 | 2.58 | 2.70 | 2.75 | 2.58 | 2.66 | 2.98 |  |
| 26 | 2.05 | 2.05 | 2.07 | 2.06 | 2.04 | 2.06 | 2.04 | 2.04 | 2.06 | 2.00 | 2.03 | 2.04 | 2.05 | 2.04 | 2.04 | 2.04 | 2.06 | 2.07 | 2.05 | 2.06 |  |
| 27 | 2.48 | 2.61 | 2.57 | 2.52 | 2.55 | 2.63 | 2.67 | 2.59 | 2.54 | 2.51 | 2.46 | 2.58 | 2.54 | 2.59 | 2.58 | 2.62 | 2.64 | 2.65 | 2.66 | 2.68 |  |
| 28 | 2.01 | 2.00 | 1.99 | 2.04 | 1.99 | 1.99 | 1.98 | 1.92 | 1.99 | 2.01 | 2.00 | 1.98 | 1.94 | 1.99 | 1.97 | 1.96 | 1.99 | 2.00 | 2.00 | 2.02 |  |
| 29 | 3.66 | 3.53 | 3.54 | 3.61 | 3.58 | 3.77 | 3.78 | 3.67 | 3.59 | 3.58 | 3.60 | 3.28 | 3.48 | 3.47 | 3.48 | 3.61 | 3.37 | 3.33 | 3.42 | 3.35 |  |
| 30 | 1.92 | 1.94 | 1.93 | 1.95 | 2.02 | 1.93 | 1.91 | 1.93 | 1.96 | 1.92 | 1.90 | 1.86 | 1.94 | 1.96 | 1.91 | 1.92 | 1.90 | 1.88 | 1.90 | 1.89 |  |
| 31 | 2.01 | 2.00 | 1.94 | 1.89 | 1.88 | 1.87 | 1.86 | 1.82 | 1.83 | 1.84 | 1.86 | 1.83 | 1.84 | 1.88 | 1.87 | 1.79 | 1.86 | 1.89 | 1.88 | 1.90 |  |
| 32 | 1.54 | 1.54 | 1.55 | 1.52 | 1.51 | 1.51 | 1.54 | 1.56 | 1.57 | 1.56 | 1.57 | 1.56 | 1.55 | 1.56 | 1.54 | 1.57 | 1.57 | 1.55 | 1.57 | 1.60 |  |
| 33 | 2.43 | 2.75 | 2.81 | 2.67 | 2.43 | 2.46 | 2.46 | 2.51 | 2.43 | 2.32 | 2.30 | 2.32 | 2.22 | 2.24 | 2.27 | 2.26 | 2.44 | 2.45 | 2.40 | 2.42 |  |
| 34 | 1.37 | 1.37 | 1.37 | 1.37 | 1.37 | 1.37 | 1.36 | 1.36 | 1.37 | 1.37 | 1.38 | 1.39 | 1.39 | 1.38 | 1.41 | 1.43 | 1.40 | 1.42 | 1.42 | 1.42 |  |
| 35 | 1.28 | 1.31 | 1.25 | 1.29 | 1.26 | 1.25 | 1.26 | 1.27 | 1.30 | 1.29 | 1.28 | 1.27 | 1.24 | 1.23 | 1.24 | 1.27 | 1.22 | 1.26 | 1.26 | 1.25 |  |
| 36 | 1.34 | 1.35 | 1.31 | 1.31 | 1.32 | 1.33 | 1.31 | 1.31 | 1.33 | 1.32 | 1.32 | 1.31 | 1.30 | 1.31 | 1.30 | 1.31 | 1.30 | 1.33 | 1.33 | 1.33 |  |
| 37 | 1.27 | 1.26 | 1.29 | 1.28 | 1.26 | 1.30 | 1.28 | 1.29 | 1.29 | 1.28 | 1.26 | 1.27 | 1.27 | 1.27 | 1.27 | 1.27 | 1.27 | 1.27 | 1.27 | 1.27 |  |
| 38 | 1.23 | 1.23 | 1.21 | 1.21 | 1.21 | 1.21 | 1.20 | 1.21 | 1.22 | 1.22 | 1.21 | 1.21 | 1.22 | 1.20 | 1.21 | 1.22 | 1.20 | 1.21 | 1.21 | 1.21 |  |
| 39 | 1.62 | 1.66 | 1.84 | 1.88 | 1.88 | 1.71 | 1.54 | 1.61 | 1.56 | 1.59 | 1.73 | 1.68 | 1.51 | 1.45 | 1.44 | 1.50 | 1.47 | 1.52 | 1.48 | 1.51 |  |
| 40 | 1.71 | 1.76 | 1.79 | 1.83 | 1.80 | 1.78 | 1.70 | 1.72 | 1.72 | 1.74 | 1.80 | 1.82 | 1.77 | 1.72 | 1.73 | 1.78 | 1.76 | 1.72 | 1.70 | 1.72 |  |
| 41 | 2.45 | 2.17 | 2.20 | 2.23 | 2.17 | 2.14 | 2.14 | 2.14 | 2.07 | 2.04 | 2.00 | 2.10 | 2.10 | 2.12 | 2.23 | 2.23 | 2.21 | 2.24 | 2.23 | 2.33 |  |
| 42 | 2.90 | 2.91 | 2.86 | 2.75 | 2.72 | 2.63 | 2.67 | 2.67 | 2.52 | 2.46 | 2.44 | 2.47 | 2.41 | 2.36 | 2.43 | 2.32 | 2.30 | 2.33 | 2.30 | 2.32 |  |
| 43 | 2.79 | 2.92 | 2.77 | 2.78 | 2.74 | 2.86 | 2.77 | 2.80 | 2.75 | 2.70 | 2.63 | 2.70 | 2.59 | 2.52 | 2.58 | 2.69 | 2.57 | 2.80 | 2.72 | 2.51 |  |
| 44 | 2.53 | 2.60 | 2.50 | 2.52 | 2.54 | 2.54 | 2.42 | 2.46 | 2.34 | 2.49 | 2.20 | 2.25 | 2.50 | 2.32 | 2.51 | 2.63 | 2.14 | 2.14 | 2.14 | 2.14 |  |
| 45 | 2.13 | 2.12 | 2.05 | 2.07 | 2.01 | 2.00 | 1.99 | 1.94 | 1.91 | 1.90 | 1.87 | 1.96 | 2.01 | 1.97 | 1.99 | 2.00 | 1.91 | 1.92 | 1.85 | 1.86 |  |
| 46 | 2.73 | 2.52 | 2.76 | 2.48 | 2.31 | 2.78 | 2.63 | 2.60 | 2.80 | 2.85 | 2.65 | 2.48 | 2.50 | 2.29 | 2.35 | 2.30 | 2.40 | 2.47 | 2.45 | 2.58 |  |
| 49 | 2.03 | 1.97 | 2.02 | 1.97 | 1.87 | 1.78 | 1.76 | 1.75 | 1.77 | 1.73 | 1.77 | 1.73 | 1.74 | 1.76 | 1.76 | 1.77 | 1.79 | 1.80 | 1.82 | 1.82 |  |
| 50 | 2.24 | 2.19 | 2.15 | 2.11 | 2.06 | 1.97 | 1.99 | 1.97 | 1.97 | 1.91 | 1.94 | 1.86 | 1.85 | 1.85 | 1.85 | 1.85 | 1.88 | 1.88 | 1.93 | 1.94 |  |
| 51 | 2.94 | 2.84 | 2.75 | 2.67 | 2.56 | 2.53 | 2.53 | 2.54 | 2.52 | 2.41 | 2.46 | 2.35 | 2.42 | 2.39 | 2.40 | 2.42 | 2.41 | 2.44 | 2.50 | 2.52 |  |
| 52 | 4.39 | 4.34 | 4.20 | 4.11 | 4.03 | 4.09 | 3.96 | 3.85 | 3.72 | 3.53 | 3.66 | 3.35 | 3.44 | 3.21 | 3.25 | 3.26 | 3.32 | 3.38 | 3.42 | 3.49 |  |
| 53 | 3.14 | 3.09 | 3.11 | 3.08 | 3.01 | 3.05 | 2.91 | 2.87 | 2.81 | 2.72 | 2.79 | 2.61 | 2.64 | 2.51 | 2.53 | 2.51 | 2.53 | 2.56 | 2.65 | 2.66 |  |
| 59 | 3.42 | 3.45 | 3.35 | 3.25 | 3.20 | 3.30 | 3.30 | 3.29 | 3.37 | 3.43 | 3.22 | 3.26 | 3.30 | 3.29 | 3.53 | 3.64 | 3.47 | 3.42 | 3.52 | 3.33 |  |
| 60 | 3.16 | 3.17 | 3.14 | 3.03 | 3.18 | 3.21 | 3.17 | 3.22 | 3.12 | 3.10 | 3.10 | 3.14 | 3.12 | 3.09 | 3.09 | 3.09 | 3.12 | 3.19 | 3.17 | 3.15 |  |
| 62 | 3.61 | 3.27 | 3.15 | 3.23 | 3.34 | 3.50 | 3.58 | 3.71 | 3.80 | 3.60 | 3.45 | 3.46 | 3.38 | 3.45 | 3.43 | 3.40 | 3.42 | 3.39 | 3.47 | 3.81 |  |
| 65 | 4.58 | 4.66 | 4.43 | 4.50 | 4.33 | 4.39 | 4.67 | 4.58 | 4.52 | 4.79 | 4.48 | 4.67 | 4.50 | 4.45 | 4.30 | 4.47 | 4.31 | 4.48 | 4.47 | 4.46 |  |
| 66 | 2.85 | 2.35 | 2.63 | 2.71 | 2.61 | 2.45 | 2.57 | 2.61 | 2.57 | 2.57 | 2.51 | 2.43 | 2.47 | 2.54 | 2.34 | 2.52 | 2.44 | 2.44 | 2.42 | 2.44 |  |
| Year  LME | 1950 | 1951 | 1952 | 1953 | 1954 | 1955 | 1956 | 1957 | 1958 | 1959 | 1960 | 1961 | 1962 | 1963 | 1964 | 1965 | 1966 | 1967 | 1968 | 1969 | 1970 |
| 1 | 0.039 | 0.038 | 0.038 | 0.034 | 0.028 | 0.028 | 0.028 | 0.028 | 0.023 | 0.015 | 0.011 | 0.012 | 0.013 | 0.018 | 0.019 | 0.019 | 0.018 | 0.017 | 0.019 | 0.019 | 0.020 |
| 2 | 0.021 | 0.021 | 0.020 | 0.022 | 0.022 | 0.020 | 0.020 | 0.020 | 0.022 | 0.020 | 0.017 | 0.018 | 0.018 | 0.019 | 0.020 | 0.016 | 0.016 | 0.015 | 0.017 | 0.015 | 0.017 |
| 3 | 0.003 | 0.003 | 0.005 | 0.006 | 0.005 | 0.005 | 0.006 | 0.006 | 0.006 | 0.007 | 0.006 | 0.006 | 0.007 | 0.007 | 0.007 | 0.007 | 0.007 | 0.007 | 0.007 | 0.006 | 0.006 |
| 4 | 0.003 | 0.003 | 0.005 | 0.006 | 0.006 | 0.006 | 0.006 | 0.006 | 0.006 | 0.005 | 0.006 | 0.006 | 0.006 | 0.006 | 0.005 | 0.005 | 0.005 | 0.005 | 0.004 | 0.005 | 0.005 |
| 5 | 0.007 | 0.006 | 0.007 | 0.007 | 0.007 | 0.007 | 0.007 | 0.007 | 0.007 | 0.007 | 0.007 | 0.007 | 0.006 | 0.006 | 0.006 | 0.006 | 0.006 | 0.006 | 0.006 | 0.006 | 0.006 |
| 6 | 0.008 | 0.006 | 0.006 | 0.007 | 0.007 | 0.007 | 0.006 | 0.007 | 0.006 | 0.006 | 0.007 | 0.006 | 0.007 | 0.006 | 0.007 | 0.007 | 0.007 | 0.007 | 0.007 | 0.007 | 0.007 |
| 7 | 0.009 | 0.008 | 0.009 | 0.008 | 0.008 | 0.008 | 0.008 | 0.008 | 0.008 | 0.008 | 0.008 | 0.008 | 0.008 | 0.008 | 0.009 | 0.009 | 0.009 | 0.010 | 0.010 | 0.010 | 0.010 |
| 8 | 0.008 | 0.008 | 0.008 | 0.008 | 0.008 | 0.008 | 0.008 | 0.008 | 0.007 | 0.007 | 0.007 | 0.008 | 0.008 | 0.008 | 0.008 | 0.009 | 0.009 | 0.010 | 0.010 | 0.011 | 0.011 |
| 9 | 0.009 | 0.009 | 0.009 | 0.009 | 0.009 | 0.009 | 0.009 | 0.009 | 0.009 | 0.008 | 0.009 | 0.009 | 0.010 | 0.010 | 0.010 | 0.010 | 0.011 | 0.012 | 0.013 | 0.013 | 0.014 |
| 10 | 0.003 | 0.003 | 0.003 | 0.003 | 0.003 | 0.003 | 0.003 | 0.004 | 0.004 | 0.004 | 0.004 | 0.005 | 0.005 | 0.005 | 0.005 | 0.006 | 0.006 | 0.006 | 0.004 | 0.005 | 0.005 |
| 11 | 0.003 | 0.003 | 0.004 | 0.004 | 0.004 | 0.004 | 0.004 | 0.003 | 0.003 | 0.003 | 0.003 | 0.003 | 0.003 | 0.003 | 0.003 | 0.003 | 0.003 | 0.003 | 0.002 | 0.002 | 0.002 |
| 12 | 0.003 | 0.003 | 0.003 | 0.004 | 0.004 | 0.004 | 0.004 | 0.003 | 0.003 | 0.003 | 0.003 | 0.003 | 0.003 | 0.003 | 0.004 | 0.003 | 0.003 | 0.003 | 0.003 | 0.004 | 0.004 |
| 13 | 0.006 | 0.006 | 0.006 | 0.006 | 0.006 | 0.005 | 0.005 | 0.005 | 0.006 | 0.005 | 0.006 | 0.005 | 0.006 | 0.003 | 0.003 | 0.005 | 0.005 | 0.006 | 0.006 | 0.005 | 0.006 |
| 14 | 0.010 | 0.012 | 0.012 | 0.013 | 0.012 | 0.010 | 0.010 | 0.009 | 0.009 | 0.009 | 0.009 | 0.009 | 0.008 | 0.009 | 0.007 | 0.007 | 0.008 | 0.008 | 0.008 | 0.007 | 0.008 |
| 15 | 0.004 | 0.004 | 0.004 | 0.004 | 0.004 | 0.005 | 0.004 | 0.005 | 0.005 | 0.004 | 0.005 | 0.005 | 0.005 | 0.005 | 0.005 | 0.005 | 0.005 | 0.006 | 0.005 | 0.005 | 0.005 |
| 16 | 0.004 | 0.004 | 0.004 | 0.004 | 0.004 | 0.004 | 0.004 | 0.004 | 0.004 | 0.004 | 0.004 | 0.004 | 0.004 | 0.004 | 0.004 | 0.004 | 0.004 | 0.004 | 0.004 | 0.004 | 0.004 |
| 17 | 0.003 | 0.003 | 0.003 | 0.004 | 0.004 | 0.005 | 0.004 | 0.004 | 0.004 | 0.004 | 0.004 | 0.005 | 0.004 | 0.004 | 0.005 | 0.004 | 0.004 | 0.004 | 0.004 | 0.004 | 0.004 |
| 18 | 0.012 | 0.012 | 0.012 | 0.012 | 0.011 | 0.011 | 0.012 | 0.012 | 0.011 | 0.011 | 0.012 | 0.012 | 0.013 | 0.012 | 0.013 | 0.013 | 0.014 | 0.015 | 0.016 | 0.016 | 0.017 |
| 19 | 0.014 | 0.016 | 0.016 | 0.018 | 0.016 | 0.021 | 0.022 | 0.019 | 0.013 | 0.015 | 0.013 | 0.016 | 0.013 | 0.012 | 0.014 | 0.014 | 0.013 | 0.011 | 0.012 | 0.013 | 0.013 |
| 20 | 0.013 | 0.014 | 0.013 | 0.012 | 0.014 | 0.011 | 0.012 | 0.012 | 0.014 | 0.017 | 0.019 | 0.019 | 0.018 | 0.019 | 0.015 | 0.019 | 0.019 | 0.019 | 0.019 | 0.020 | 0.020 |
| 21 | 0.011 | 0.012 | 0.012 | 0.010 | 0.012 | 0.012 | 0.015 | 0.015 | 0.014 | 0.015 | 0.014 | 0.014 | 0.012 | 0.013 | 0.013 | 0.014 | 0.014 | 0.013 | 0.012 | 0.014 | 0.014 |
| 22 | 0.018 | 0.016 | 0.018 | 0.018 | 0.018 | 0.018 | 0.018 | 0.018 | 0.017 | 0.017 | 0.017 | 0.016 | 0.016 | 0.017 | 0.016 | 0.015 | 0.016 | 0.015 | 0.015 | 0.016 | 0.015 |
| 23 | 0.017 | 0.017 | 0.018 | 0.018 | 0.017 | 0.017 | 0.017 | 0.018 | 0.018 | 0.018 | 0.018 | 0.018 | 0.017 | 0.016 | 0.017 | 0.018 | 0.017 | 0.016 | 0.016 | 0.017 | 0.017 |
| 24 | 0.010 | 0.010 | 0.010 | 0.010 | 0.011 | 0.011 | 0.010 | 0.011 | 0.011 | 0.010 | 0.009 | 0.010 | 0.009 | 0.009 | 0.010 | 0.010 | 0.010 | 0.010 | 0.010 | 0.010 | 0.010 |
| 25 | 0.008 | 0.008 | 0.008 | 0.008 | 0.008 | 0.008 | 0.008 | 0.008 | 0.009 | 0.009 | 0.009 | 0.008 | 0.008 | 0.008 | 0.009 | 0.010 | 0.010 | 0.009 | 0.010 | 0.009 | 0.009 |
| 26 | 0.008 | 0.008 | 0.008 | 0.008 | 0.008 | 0.008 | 0.008 | 0.008 | 0.009 | 0.008 | 0.008 | 0.008 | 0.008 | 0.007 | 0.008 | 0.008 | 0.008 | 0.008 | 0.008 | 0.008 | 0.008 |
| 27 | 0.006 | 0.006 | 0.006 | 0.007 | 0.006 | 0.006 | 0.006 | 0.006 | 0.006 | 0.006 | 0.006 | 0.005 | 0.005 | 0.005 | 0.005 | 0.005 | 0.005 | 0.005 | 0.004 | 0.004 | 0.004 |
| 28 | 0.003 | 0.003 | 0.003 | 0.003 | 0.003 | 0.003 | 0.003 | 0.003 | 0.003 | 0.003 | 0.003 | 0.003 | 0.003 | 0.003 | 0.003 | 0.004 | 0.004 | 0.004 | 0.004 | 0.004 | 0.004 |
| 29 | 0.002 | 0.002 | 0.002 | 0.002 | 0.002 | 0.002 | 0.002 | 0.002 | 0.002 | 0.002 | 0.002 | 0.002 | 0.002 | 0.002 | 0.002 | 0.002 | 0.002 | 0.002 | 0.002 | 0.002 | 0.002 |
| 30 | 0.002 | 0.002 | 0.002 | 0.002 | 0.002 | 0.002 | 0.002 | 0.002 | 0.002 | 0.002 | 0.002 | 0.002 | 0.002 | 0.002 | 0.002 | 0.002 | 0.002 | 0.002 | 0.002 | 0.002 | 0.002 |
| 31 | 0.003 | 0.003 | 0.003 | 0.003 | 0.003 | 0.003 | 0.003 | 0.003 | 0.003 | 0.003 | 0.003 | 0.003 | 0.003 | 0.003 | 0.003 | 0.003 | 0.003 | 0.003 | 0.003 | 0.003 | 0.003 |
| 32 | 0.004 | 0.004 | 0.004 | 0.004 | 0.004 | 0.004 | 0.004 | 0.004 | 0.003 | 0.003 | 0.003 | 0.003 | 0.003 | 0.004 | 0.003 | 0.003 | 0.003 | 0.003 | 0.003 | 0.003 | 0.003 |
| 33 | 0.001 | 0.001 | 0.001 | 0.001 | 0.001 | 0.001 | 0.001 | 0.001 | 0.001 | 0.001 | 0.001 | 0.001 | 0.002 | 0.002 | 0.002 | 0.002 | 0.001 | 0.001 | 0.001 | 0.001 | 0.001 |
| 34 | 0.004 | 0.004 | 0.004 | 0.004 | 0.004 | 0.004 | 0.004 | 0.004 | 0.004 | 0.004 | 0.004 | 0.004 | 0.004 | 0.004 | 0.004 | 0.004 | 0.004 | 0.005 | 0.005 | 0.004 | 0.005 |
| 35 | 0.006 | 0.006 | 0.006 | 0.006 | 0.006 | 0.006 | 0.006 | 0.006 | 0.006 | 0.005 | 0.005 | 0.005 | 0.005 | 0.005 | 0.005 | 0.005 | 0.005 | 0.005 | 0.005 | 0.006 | 0.008 |
| 36 | 0.008 | 0.008 | 0.008 | 0.008 | 0.009 | 0.009 | 0.009 | 0.009 | 0.009 | 0.009 | 0.008 | 0.008 | 0.008 | 0.008 | 0.007 | 0.007 | 0.007 | 0.007 | 0.008 | 0.008 | 0.009 |
| 37 | 0.006 | 0.006 | 0.006 | 0.006 | 0.006 | 0.006 | 0.006 | 0.006 | 0.006 | 0.006 | 0.006 | 0.006 | 0.006 | 0.006 | 0.005 | 0.006 | 0.006 | 0.006 | 0.006 | 0.006 | 0.006 |
| 38 | 0.005 | 0.005 | 0.005 | 0.005 | 0.005 | 0.005 | 0.005 | 0.005 | 0.005 | 0.005 | 0.005 | 0.005 | 0.005 | 0.005 | 0.005 | 0.005 | 0.005 | 0.005 | 0.005 | 0.005 | 0.005 |
| 39 | 0.003 | 0.003 | 0.003 | 0.003 | 0.003 | 0.004 | 0.004 | 0.004 | 0.004 | 0.004 | 0.004 | 0.003 | 0.003 | 0.003 | 0.003 | 0.003 | 0.003 | 0.004 | 0.004 | 0.004 | 0.004 |
| 40 | 0.004 | 0.004 | 0.004 | 0.004 | 0.004 | 0.004 | 0.004 | 0.004 | 0.004 | 0.004 | 0.004 | 0.004 | 0.004 | 0.004 | 0.004 | 0.004 | 0.004 | 0.004 | 0.004 | 0.004 | 0.005 |
| 41 | 0.004 | 0.004 | 0.004 | 0.004 | 0.004 | 0.004 | 0.004 | 0.004 | 0.004 | 0.004 | 0.004 | 0.004 | 0.004 | 0.004 | 0.004 | 0.004 | 0.004 | 0.004 | 0.004 | 0.004 | 0.005 |
| 42 | 0.006 | 0.006 | 0.006 | 0.006 | 0.006 | 0.006 | 0.006 | 0.006 | 0.006 | 0.006 | 0.006 | 0.006 | 0.006 | 0.006 | 0.007 | 0.006 | 0.006 | 0.007 | 0.007 | 0.007 | 0.007 |
| 43 | 0.006 | 0.006 | 0.006 | 0.005 | 0.005 | 0.005 | 0.005 | 0.005 | 0.005 | 0.005 | 0.005 | 0.005 | 0.005 | 0.005 | 0.005 | 0.005 | 0.005 | 0.006 | 0.006 | 0.006 | 0.007 |
| 44 | 0.005 | 0.005 | 0.005 | 0.005 | 0.005 | 0.005 | 0.005 | 0.005 | 0.004 | 0.004 | 0.004 | 0.005 | 0.005 | 0.005 | 0.005 | 0.005 | 0.005 | 0.005 | 0.005 | 0.006 | 0.006 |
| 45 | 0.003 | 0.003 | 0.003 | 0.003 | 0.003 | 0.003 | 0.003 | 0.003 | 0.003 | 0.003 | 0.003 | 0.003 | 0.003 | 0.003 | 0.003 | 0.003 | 0.003 | 0.004 | 0.004 | 0.004 | 0.004 |
| 46 | 0.007 | 0.007 | 0.007 | 0.007 | 0.007 | 0.007 | 0.007 | 0.007 | 0.007 | 0.007 | 0.007 | 0.007 | 0.007 | 0.007 | 0.008 | 0.008 | 0.008 | 0.007 | 0.007 | 0.008 | 0.007 |
| 49 | 0.005 | 0.005 | 0.005 | 0.005 | 0.006 | 0.006 | 0.006 | 0.006 | 0.006 | 0.007 | 0.006 | 0.006 | 0.007 | 0.008 | 0.007 | 0.008 | 0.007 | 0.007 | 0.007 | 0.007 | 0.007 |
| 50 | 0.012 | 0.012 | 0.012 | 0.011 | 0.012 | 0.012 | 0.012 | 0.012 | 0.013 | 0.013 | 0.013 | 0.012 | 0.013 | 0.014 | 0.014 | 0.014 | 0.014 | 0.014 | 0.014 | 0.013 | 0.013 |
| 51 | 0.015 | 0.015 | 0.015 | 0.015 | 0.015 | 0.016 | 0.015 | 0.016 | 0.016 | 0.016 | 0.016 | 0.016 | 0.017 | 0.018 | 0.017 | 0.017 | 0.017 | 0.017 | 0.017 | 0.017 | 0.017 |
| 52 | 0.016 | 0.017 | 0.017 | 0.015 | 0.015 | 0.015 | 0.015 | 0.015 | 0.015 | 0.016 | 0.016 | 0.016 | 0.017 | 0.017 | 0.017 | 0.018 | 0.018 | 0.018 | 0.018 | 0.019 | 0.018 |
| 53 | 0.017 | 0.017 | 0.016 | 0.015 | 0.014 | 0.014 | 0.015 | 0.016 | 0.014 | 0.014 | 0.015 | 0.016 | 0.018 | 0.019 | 0.017 | 0.017 | 0.019 | 0.020 | 0.020 | 0.019 | 0.019 |
| 59 | 0.016 | 0.015 | 0.016 | 0.016 | 0.017 | 0.018 | 0.016 | 0.016 | 0.016 | 0.016 | 0.017 | 0.017 | 0.016 | 0.016 | 0.016 | 0.016 | 0.015 | 0.015 | 0.015 | 0.016 | 0.016 |
| 60 | 0.014 | 0.015 | 0.014 | 0.014 | 0.014 | 0.014 | 0.014 | 0.014 | 0.015 | 0.014 | 0.015 | 0.016 | 0.015 | 0.014 | 0.014 | 0.014 | 0.014 | 0.014 | 0.014 | 0.014 | 0.014 |
| 62 | 0.003 | 0.003 | 0.003 | 0.003 | 0.003 | 0.003 | 0.003 | 0.003 | 0.003 | 0.003 | 0.003 | 0.003 | 0.003 | 0.003 | 0.003 | 0.003 | 0.003 | 0.003 | 0.003 | 0.004 | 0.004 |
| 65 | 0.038 | 0.032 | 0.036 | 0.031 | 0.027 | 0.028 | 0.029 | 0.026 | 0.023 | 0.018 | 0.015 | 0.012 | 0.013 | 0.016 | 0.015 | 0.023 | 0.016 | 0.014 | 0.017 | 0.016 | 0.018 |
| 66 | 0.018 | 0.017 | 0.018 | 0.018 | 0.017 | 0.017 | 0.018 | 0.018 | 0.017 | 0.018 | 0.019 | 0.019 | 0.018 | 0.018 | 0.018 | 0.017 | 0.019 | 0.020 | 0.022 | 0.021 | 0.025 |
| Year  LME | 1971 | 1972 | 1973 | 1974 | 1975 | 1976 | 1977 | 1978 | 1979 | 1980 | 1981 | 1982 | 1983 | 1984 | 1985 | 1986 | 1987 | 1988 | 1989 | 1990 |  |
| 1 | 0.021 | 0.017 | 0.018 | 0.018 | 0.017 | 0.018 | 0.019 | 0.019 | 0.020 | 0.020 | 0.019 | 0.020 | 0.020 | 0.018 | 0.018 | 0.019 | 0.022 | 0.022 | 0.024 | 0.024 |  |
| 2 | 0.018 | 0.016 | 0.018 | 0.017 | 0.014 | 0.017 | 0.019 | 0.020 | 0.019 | 0.017 | 0.018 | 0.019 | 0.017 | 0.016 | 0.018 | 0.015 | 0.012 | 0.018 | 0.019 | 0.019 |  |
| 3 | 0.007 | 0.007 | 0.006 | 0.008 | 0.008 | 0.008 | 0.009 | 0.008 | 0.007 | 0.007 | 0.007 | 0.007 | 0.007 | 0.007 | 0.008 | 0.009 | 0.008 | 0.010 | 0.010 | 0.009 |  |
| 4 | 0.004 | 0.005 | 0.005 | 0.005 | 0.005 | 0.005 | 0.006 | 0.005 | 0.005 | 0.005 | 0.004 | 0.005 | 0.004 | 0.004 | 0.004 | 0.004 | 0.004 | 0.004 | 0.004 | 0.004 |  |
| 5 | 0.006 | 0.006 | 0.006 | 0.007 | 0.006 | 0.006 | 0.008 | 0.005 | 0.005 | 0.005 | 0.005 | 0.005 | 0.005 | 0.005 | 0.005 | 0.006 | 0.005 | 0.005 | 0.005 | 0.005 |  |
| 6 | 0.008 | 0.008 | 0.008 | 0.007 | 0.007 | 0.007 | 0.007 | 0.007 | 0.008 | 0.008 | 0.008 | 0.009 | 0.009 | 0.008 | 0.009 | 0.009 | 0.010 | 0.010 | 0.010 | 0.010 |  |
| 7 | 0.010 | 0.010 | 0.010 | 0.011 | 0.011 | 0.011 | 0.011 | 0.011 | 0.012 | 0.011 | 0.012 | 0.011 | 0.011 | 0.010 | 0.011 | 0.011 | 0.011 | 0.011 | 0.012 | 0.011 |  |
| 8 | 0.012 | 0.011 | 0.012 | 0.012 | 0.013 | 0.014 | 0.015 | 0.017 | 0.019 | 0.018 | 0.018 | 0.018 | 0.017 | 0.016 | 0.017 | 0.016 | 0.015 | 0.016 | 0.016 | 0.015 |  |
| 9 | 0.014 | 0.014 | 0.014 | 0.015 | 0.015 | 0.016 | 0.018 | 0.018 | 0.019 | 0.019 | 0.018 | 0.018 | 0.018 | 0.017 | 0.018 | 0.017 | 0.016 | 0.018 | 0.018 | 0.017 |  |
| 10 | 0.006 | 0.006 | 0.004 | 0.006 | 0.006 | 0.007 | 0.008 | 0.006 | 0.006 | 0.006 | 0.006 | 0.006 | 0.004 | 0.004 | 0.003 | 0.003 | 0.003 | 0.003 | 0.004 | 0.005 |  |
| 11 | 0.002 | 0.003 | 0.003 | 0.003 | 0.003 | 0.003 | 0.003 | 0.003 | 0.003 | 0.003 | 0.003 | 0.003 | 0.003 | 0.003 | 0.002 | 0.003 | 0.003 | 0.003 | 0.003 | 0.003 |  |
| 12 | 0.004 | 0.004 | 0.004 | 0.004 | 0.004 | 0.004 | 0.005 | 0.004 | 0.004 | 0.004 | 0.004 | 0.004 | 0.004 | 0.004 | 0.004 | 0.004 | 0.005 | 0.005 | 0.004 | 0.004 |  |
| 13 | 0.006 | 0.005 | 0.005 | 0.005 | 0.006 | 0.005 | 0.005 | 0.005 | 0.004 | 0.004 | 0.004 | 0.004 | 0.004 | 0.004 | 0.004 | 0.004 | 0.004 | 0.004 | 0.004 | 0.004 |  |
| 14 | 0.008 | 0.006 | 0.008 | 0.007 | 0.009 | 0.009 | 0.008 | 0.013 | 0.012 | 0.009 | 0.009 | 0.013 | 0.013 | 0.012 | 0.013 | 0.013 | 0.014 | 0.014 | 0.014 | 0.015 |  |
| 15 | 0.005 | 0.005 | 0.004 | 0.005 | 0.005 | 0.005 | 0.005 | 0.005 | 0.005 | 0.005 | 0.005 | 0.006 | 0.004 | 0.004 | 0.006 | 0.006 | 0.006 | 0.007 | 0.007 | 0.007 |  |
| 16 | 0.004 | 0.004 | 0.004 | 0.004 | 0.004 | 0.004 | 0.004 | 0.004 | 0.004 | 0.004 | 0.004 | 0.004 | 0.004 | 0.004 | 0.004 | 0.004 | 0.004 | 0.005 | 0.005 | 0.005 |  |
| 17 | 0.004 | 0.004 | 0.004 | 0.004 | 0.004 | 0.004 | 0.004 | 0.004 | 0.004 | 0.004 | 0.004 | 0.004 | 0.004 | 0.004 | 0.004 | 0.004 | 0.004 | 0.004 | 0.004 | 0.004 |  |
| 18 | 0.017 | 0.017 | 0.018 | 0.019 | 0.021 | 0.022 | 0.023 | 0.025 | 0.025 | 0.026 | 0.027 | 0.028 | 0.028 | 0.027 | 0.026 | 0.025 | 0.023 | 0.025 | 0.027 | 0.024 |  |
| 19 | 0.012 | 0.011 | 0.014 | 0.010 | 0.011 | 0.011 | 0.012 | 0.013 | 0.014 | 0.014 | 0.013 | 0.013 | 0.014 | 0.015 | 0.015 | 0.014 | 0.017 | 0.017 | 0.017 | 0.017 |  |
| 20 | 0.019 | 0.019 | 0.019 | 0.021 | 0.018 | 0.020 | 0.020 | 0.022 | 0.021 | 0.020 | 0.020 | 0.020 | 0.020 | 0.021 | 0.020 | 0.018 | 0.018 | 0.019 | 0.019 | 0.018 |  |
| 21 | 0.014 | 0.014 | 0.013 | 0.012 | 0.013 | 0.013 | 0.013 | 0.013 | 0.013 | 0.013 | 0.014 | 0.013 | 0.014 | 0.013 | 0.012 | 0.010 | 0.010 | 0.010 | 0.010 | 0.010 |  |
| 22 | 0.013 | 0.014 | 0.014 | 0.014 | 0.014 | 0.014 | 0.012 | 0.012 | 0.013 | 0.013 | 0.012 | 0.012 | 0.012 | 0.011 | 0.012 | 0.011 | 0.012 | 0.011 | 0.010 | 0.010 |  |
| 23 | 0.017 | 0.017 | 0.017 | 0.016 | 0.017 | 0.017 | 0.017 | 0.015 | 0.015 | 0.015 | 0.014 | 0.014 | 0.013 | 0.014 | 0.014 | 0.017 | 0.017 | 0.017 | 0.015 | 0.016 |  |
| 24 | 0.011 | 0.010 | 0.011 | 0.011 | 0.011 | 0.011 | 0.011 | 0.012 | 0.012 | 0.012 | 0.012 | 0.012 | 0.012 | 0.012 | 0.011 | 0.011 | 0.012 | 0.011 | 0.011 | 0.012 |  |
| 25 | 0.008 | 0.009 | 0.009 | 0.008 | 0.008 | 0.007 | 0.007 | 0.008 | 0.007 | 0.009 | 0.009 | 0.008 | 0.008 | 0.008 | 0.009 | 0.008 | 0.008 | 0.008 | 0.008 | 0.008 |  |
| 26 | 0.008 | 0.009 | 0.009 | 0.009 | 0.009 | 0.009 | 0.009 | 0.009 | 0.009 | 0.009 | 0.009 | 0.009 | 0.009 | 0.008 | 0.008 | 0.008 | 0.008 | 0.009 | 0.009 | 0.008 |  |
| 27 | 0.004 | 0.003 | 0.004 | 0.004 | 0.004 | 0.004 | 0.004 | 0.003 | 0.004 | 0.003 | 0.004 | 0.004 | 0.004 | 0.004 | 0.004 | 0.005 | 0.005 | 0.005 | 0.005 | 0.005 |  |
| 28 | 0.004 | 0.004 | 0.004 | 0.004 | 0.004 | 0.004 | 0.004 | 0.004 | 0.004 | 0.004 | 0.004 | 0.004 | 0.004 | 0.004 | 0.004 | 0.004 | 0.004 | 0.004 | 0.004 | 0.004 |  |
| 29 | 0.002 | 0.002 | 0.002 | 0.002 | 0.002 | 0.002 | 0.002 | 0.002 | 0.002 | 0.002 | 0.002 | 0.002 | 0.002 | 0.002 | 0.002 | 0.002 | 0.002 | 0.002 | 0.002 | 0.002 |  |
| 30 | 0.003 | 0.003 | 0.003 | 0.003 | 0.003 | 0.003 | 0.003 | 0.004 | 0.004 | 0.004 | 0.004 | 0.004 | 0.004 | 0.004 | 0.004 | 0.004 | 0.004 | 0.004 | 0.004 | 0.005 |  |
| 31 | 0.003 | 0.003 | 0.003 | 0.003 | 0.003 | 0.003 | 0.003 | 0.003 | 0.003 | 0.003 | 0.003 | 0.003 | 0.003 | 0.003 | 0.003 | 0.003 | 0.003 | 0.003 | 0.003 | 0.003 |  |
| 32 | 0.003 | 0.004 | 0.004 | 0.003 | 0.004 | 0.003 | 0.003 | 0.003 | 0.003 | 0.003 | 0.003 | 0.003 | 0.003 | 0.003 | 0.003 | 0.004 | 0.003 | 0.003 | 0.003 | 0.003 |  |
| 33 | 0.001 | 0.002 | 0.002 | 0.002 | 0.002 | 0.002 | 0.002 | 0.002 | 0.002 | 0.002 | 0.002 | 0.002 | 0.002 | 0.002 | 0.002 | 0.002 | 0.002 | 0.002 | 0.002 | 0.002 |  |
| 34 | 0.005 | 0.005 | 0.006 | 0.005 | 0.005 | 0.005 | 0.005 | 0.005 | 0.005 | 0.004 | 0.004 | 0.005 | 0.005 | 0.004 | 0.004 | 0.004 | 0.004 | 0.004 | 0.005 | 0.005 |  |
| 35 | 0.008 | 0.009 | 0.009 | 0.008 | 0.008 | 0.008 | 0.007 | 0.008 | 0.007 | 0.007 | 0.007 | 0.009 | 0.009 | 0.007 | 0.007 | 0.007 | 0.007 | 0.007 | 0.007 | 0.007 |  |
| 36 | 0.009 | 0.009 | 0.009 | 0.008 | 0.009 | 0.009 | 0.008 | 0.007 | 0.007 | 0.007 | 0.007 | 0.008 | 0.008 | 0.008 | 0.008 | 0.008 | 0.008 | 0.008 | 0.007 | 0.007 |  |
| 37 | 0.006 | 0.006 | 0.005 | 0.006 | 0.006 | 0.006 | 0.006 | 0.006 | 0.006 | 0.006 | 0.006 | 0.005 | 0.005 | 0.005 | 0.005 | 0.006 | 0.006 | 0.006 | 0.005 | 0.006 |  |
| 38 | 0.005 | 0.005 | 0.005 | 0.005 | 0.005 | 0.006 | 0.006 | 0.006 | 0.005 | 0.005 | 0.005 | 0.006 | 0.006 | 0.005 | 0.005 | 0.005 | 0.005 | 0.005 | 0.005 | 0.005 |  |
| 39 | 0.005 | 0.005 | 0.005 | 0.005 | 0.005 | 0.005 | 0.005 | 0.005 | 0.005 | 0.006 | 0.005 | 0.005 | 0.005 | 0.005 | 0.005 | 0.005 | 0.006 | 0.006 | 0.005 | 0.005 |  |
| 40 | 0.005 | 0.005 | 0.005 | 0.005 | 0.005 | 0.005 | 0.005 | 0.005 | 0.006 | 0.006 | 0.006 | 0.006 | 0.006 | 0.006 | 0.007 | 0.007 | 0.008 | 0.008 | 0.008 | 0.008 |  |
| 41 | 0.005 | 0.005 | 0.005 | 0.005 | 0.005 | 0.005 | 0.005 | 0.005 | 0.005 | 0.005 | 0.005 | 0.005 | 0.005 | 0.005 | 0.006 | 0.005 | 0.005 | 0.005 | 0.005 | 0.005 |  |
| 42 | 0.007 | 0.007 | 0.008 | 0.008 | 0.007 | 0.008 | 0.007 | 0.007 | 0.008 | 0.007 | 0.007 | 0.008 | 0.008 | 0.007 | 0.007 | 0.007 | 0.007 | 0.007 | 0.007 | 0.007 |  |
| 43 | 0.007 | 0.007 | 0.007 | 0.007 | 0.007 | 0.006 | 0.006 | 0.006 | 0.006 | 0.006 | 0.006 | 0.006 | 0.006 | 0.005 | 0.005 | 0.005 | 0.005 | 0.005 | 0.005 | 0.005 |  |
| 44 | 0.006 | 0.006 | 0.006 | 0.006 | 0.006 | 0.006 | 0.006 | 0.006 | 0.006 | 0.005 | 0.006 | 0.006 | 0.005 | 0.005 | 0.005 | 0.005 | 0.005 | 0.005 | 0.005 | 0.005 |  |
| 45 | 0.004 | 0.005 | 0.005 | 0.004 | 0.005 | 0.004 | 0.004 | 0.004 | 0.004 | 0.004 | 0.004 | 0.004 | 0.004 | 0.003 | 0.003 | 0.003 | 0.003 | 0.003 | 0.003 | 0.003 |  |
| 46 | 0.007 | 0.007 | 0.007 | 0.007 | 0.007 | 0.007 | 0.007 | 0.008 | 0.007 | 0.007 | 0.007 | 0.007 | 0.007 | 0.008 | 0.007 | 0.007 | 0.007 | 0.007 | 0.008 | 0.009 |  |
| 49 | 0.007 | 0.007 | 0.006 | 0.006 | 0.006 | 0.005 | 0.005 | 0.005 | 0.005 | 0.005 | 0.005 | 0.005 | 0.005 | 0.005 | 0.005 | 0.005 | 0.005 | 0.005 | 0.005 | 0.005 |  |
| 50 | 0.013 | 0.013 | 0.012 | 0.012 | 0.011 | 0.011 | 0.010 | 0.010 | 0.010 | 0.010 | 0.010 | 0.010 | 0.009 | 0.009 | 0.010 | 0.009 | 0.010 | 0.010 | 0.010 | 0.010 |  |
| 51 | 0.016 | 0.017 | 0.016 | 0.016 | 0.015 | 0.014 | 0.013 | 0.012 | 0.011 | 0.011 | 0.011 | 0.011 | 0.011 | 0.011 | 0.011 | 0.012 | 0.012 | 0.012 | 0.012 | 0.012 |  |
| 52 | 0.017 | 0.017 | 0.017 | 0.017 | 0.016 | 0.015 | 0.013 | 0.012 | 0.011 | 0.010 | 0.010 | 0.010 | 0.010 | 0.010 | 0.011 | 0.011 | 0.011 | 0.011 | 0.011 | 0.011 |  |
| 53 | 0.017 | 0.017 | 0.018 | 0.018 | 0.019 | 0.017 | 0.018 | 0.015 | 0.013 | 0.013 | 0.012 | 0.012 | 0.012 | 0.013 | 0.014 | 0.014 | 0.015 | 0.014 | 0.015 | 0.015 |  |
| 59 | 0.015 | 0.015 | 0.016 | 0.015 | 0.015 | 0.016 | 0.016 | 0.016 | 0.016 | 0.016 | 0.016 | 0.018 | 0.016 | 0.015 | 0.016 | 0.016 | 0.017 | 0.016 | 0.016 | 0.016 |  |
| 60 | 0.013 | 0.012 | 0.012 | 0.011 | 0.011 | 0.012 | 0.011 | 0.011 | 0.012 | 0.011 | 0.012 | 0.013 | 0.013 | 0.014 | 0.014 | 0.013 | 0.014 | 0.014 | 0.014 | 0.014 |  |
| 62 | 0.004 | 0.004 | 0.005 | 0.004 | 0.004 | 0.005 | 0.005 | 0.005 | 0.006 | 0.006 | 0.006 | 0.006 | 0.006 | 0.006 | 0.006 | 0.007 | 0.006 | 0.005 | 0.005 | 0.005 |  |
| 65 | 0.016 | 0.013 | 0.014 | 0.015 | 0.014 | 0.016 | 0.017 | 0.018 | 0.019 | 0.019 | 0.020 | 0.020 | 0.021 | 0.020 | 0.017 | 0.017 | 0.018 | 0.017 | 0.019 | 0.021 |  |
| 66 | 0.024 | 0.024 | 0.024 | 0.026 | 0.027 | 0.028 | 0.029 | 0.032 | 0.033 | 0.033 | 0.034 | 0.035 | 0.035 | 0.035 | 0.031 | 0.030 | 0.030 | 0.032 | 0.033 | 0.030 |  |
| Year  LME | 1991 | 1992 | 1993 | 1994 | 1995 | 1996 | 1997 | 1998 | 1999 | 2000 | 2001 | 2002 | 2003 | 2004 | 2005 | 2006 | 2007 | 2008 | 2009 | 2010 |  |
| 1 | 0.025 | 0.024 | 0.024 | 0.025 | 0.025 | 0.025 | 0.025 | 0.025 | 0.025 | 0.024 | 0.025 | 0.025 | 0.025 | 0.026 | 0.026 | 0.026 | 0.026 | 0.025 | 0.025 | 0.026 |  |
| 2 | 0.018 | 0.019 | 0.020 | 0.019 | 0.018 | 0.016 | 0.017 | 0.016 | 0.015 | 0.015 | 0.015 | 0.016 | 0.015 | 0.015 | 0.014 | 0.015 | 0.014 | 0.014 | 0.011 | 0.012 |  |
| 3 | 0.009 | 0.009 | 0.010 | 0.010 | 0.009 | 0.010 | 0.010 | 0.006 | 0.010 | 0.010 | 0.010 | 0.010 | 0.010 | 0.010 | 0.010 | 0.009 | 0.009 | 0.009 | 0.010 | 0.010 |  |
| 4 | 0.004 | 0.004 | 0.004 | 0.004 | 0.004 | 0.004 | 0.004 | 0.003 | 0.004 | 0.004 | 0.003 | 0.004 | 0.004 | 0.004 | 0.004 | 0.004 | 0.004 | 0.004 | 0.004 | 0.003 |  |
| 5 | 0.004 | 0.005 | 0.005 | 0.005 | 0.005 | 0.006 | 0.005 | 0.006 | 0.006 | 0.006 | 0.006 | 0.006 | 0.007 | 0.007 | 0.007 | 0.007 | 0.007 | 0.007 | 0.008 | 0.007 |  |
| 6 | 0.010 | 0.010 | 0.010 | 0.010 | 0.011 | 0.011 | 0.010 | 0.010 | 0.011 | 0.010 | 0.010 | 0.010 | 0.010 | 0.010 | 0.010 | 0.011 | 0.010 | 0.011 | 0.011 | 0.011 |  |
| 7 | 0.011 | 0.011 | 0.011 | 0.012 | 0.012 | 0.012 | 0.012 | 0.011 | 0.012 | 0.013 | 0.012 | 0.013 | 0.013 | 0.013 | 0.013 | 0.013 | 0.013 | 0.013 | 0.013 | 0.013 |  |
| 8 | 0.016 | 0.016 | 0.016 | 0.018 | 0.019 | 0.019 | 0.019 | 0.019 | 0.019 | 0.019 | 0.019 | 0.019 | 0.019 | 0.021 | 0.021 | 0.021 | 0.021 | 0.022 | 0.019 | 0.019 |  |
| 9 | 0.018 | 0.017 | 0.017 | 0.019 | 0.019 | 0.020 | 0.020 | 0.020 | 0.020 | 0.019 | 0.019 | 0.019 | 0.019 | 0.021 | 0.021 | 0.021 | 0.021 | 0.021 | 0.021 | 0.021 |  |
| 10 | 0.003 | 0.003 | 0.003 | 0.004 | 0.004 | 0.003 | 0.003 | 0.004 | 0.004 | 0.003 | 0.003 | 0.003 | 0.002 | 0.003 | 0.004 | 0.003 | 0.003 | 0.004 | 0.004 | 0.004 |  |
| 11 | 0.003 | 0.002 | 0.002 | 0.002 | 0.002 | 0.003 | 0.003 | 0.002 | 0.003 | 0.002 | 0.002 | 0.003 | 0.003 | 0.003 | 0.003 | 0.003 | 0.003 | 0.003 | 0.003 | 0.003 |  |
| 12 | 0.004 | 0.004 | 0.004 | 0.004 | 0.004 | 0.004 | 0.005 | 0.005 | 0.005 | 0.004 | 0.004 | 0.005 | 0.005 | 0.004 | 0.004 | 0.005 | 0.005 | 0.004 | 0.005 | 0.004 |  |
| 13 | 0.005 | 0.005 | 0.006 | 0.006 | 0.005 | 0.005 | 0.005 | 0.004 | 0.005 | 0.006 | 0.005 | 0.007 | 0.007 | 0.008 | 0.008 | 0.008 | 0.008 | 0.008 | 0.008 | 0.008 |  |
| 14 | 0.014 | 0.016 | 0.015 | 0.016 | 0.012 | 0.013 | 0.011 | 0.012 | 0.015 | 0.013 | 0.014 | 0.015 | 0.013 | 0.009 | 0.009 | 0.011 | 0.013 | 0.011 | 0.009 | 0.009 |  |
| 15 | 0.007 | 0.008 | 0.006 | 0.006 | 0.007 | 0.006 | 0.007 | 0.006 | 0.005 | 0.006 | 0.005 | 0.005 | 0.006 | 0.005 | 0.004 | 0.004 | 0.006 | 0.006 | 0.006 | 0.005 |  |
| 16 | 0.005 | 0.005 | 0.004 | 0.004 | 0.004 | 0.004 | 0.004 | 0.004 | 0.004 | 0.004 | 0.004 | 0.004 | 0.004 | 0.004 | 0.004 | 0.004 | 0.004 | 0.004 | 0.004 | 0.004 |  |
| 17 | 0.004 | 0.004 | 0.004 | 0.004 | 0.004 | 0.004 | 0.005 | 0.005 | 0.004 | 0.005 | 0.005 | 0.005 | 0.005 | 0.005 | 0.005 | 0.005 | 0.005 | 0.005 | 0.005 | 0.005 |  |
| 18 | 0.022 | 0.022 | 0.020 | 0.020 | 0.021 | 0.023 | 0.023 | 0.022 | 0.022 | 0.020 | 0.020 | 0.019 | 0.019 | 0.021 | 0.021 | 0.022 | 0.024 | 0.025 | 0.023 | 0.023 |  |
| 19 | 0.016 | 0.015 | 0.015 | 0.016 | 0.015 | 0.016 | 0.016 | 0.015 | 0.014 | 0.015 | 0.016 | 0.015 | 0.016 | 0.017 | 0.018 | 0.016 | 0.017 | 0.016 | 0.019 | 0.015 |  |
| 20 | 0.020 | 0.019 | 0.020 | 0.019 | 0.018 | 0.019 | 0.016 | 0.019 | 0.020 | 0.020 | 0.019 | 0.021 | 0.021 | 0.020 | 0.021 | 0.022 | 0.021 | 0.021 | 0.023 | 0.021 |  |
| 21 | 0.012 | 0.012 | 0.013 | 0.011 | 0.011 | 0.011 | 0.011 | 0.012 | 0.012 | 0.013 | 0.013 | 0.012 | 0.014 | 0.013 | 0.014 | 0.012 | 0.013 | 0.012 | 0.013 | 0.013 |  |
| 22 | 0.010 | 0.010 | 0.011 | 0.011 | 0.011 | 0.011 | 0.010 | 0.011 | 0.011 | 0.011 | 0.011 | 0.010 | 0.012 | 0.012 | 0.014 | 0.012 | 0.013 | 0.012 | 0.012 | 0.012 |  |
| 23 | 0.016 | 0.016 | 0.016 | 0.017 | 0.017 | 0.017 | 0.017 | 0.017 | 0.017 | 0.017 | 0.017 | 0.017 | 0.017 | 0.017 | 0.018 | 0.017 | 0.017 | 0.017 | 0.017 | 0.017 |  |
| 24 | 0.011 | 0.011 | 0.012 | 0.012 | 0.011 | 0.011 | 0.011 | 0.012 | 0.012 | 0.012 | 0.012 | 0.012 | 0.012 | 0.013 | 0.011 | 0.012 | 0.012 | 0.012 | 0.011 | 0.012 |  |
| 25 | 0.008 | 0.008 | 0.007 | 0.007 | 0.008 | 0.008 | 0.008 | 0.007 | 0.008 | 0.009 | 0.008 | 0.008 | 0.009 | 0.008 | 0.008 | 0.008 | 0.008 | 0.008 | 0.007 | 0.007 |  |
| 26 | 0.008 | 0.008 | 0.008 | 0.008 | 0.008 | 0.008 | 0.008 | 0.008 | 0.008 | 0.009 | 0.008 | 0.008 | 0.008 | 0.008 | 0.008 | 0.008 | 0.008 | 0.008 | 0.008 | 0.008 |  |
| 27 | 0.006 | 0.006 | 0.006 | 0.006 | 0.006 | 0.005 | 0.005 | 0.005 | 0.005 | 0.005 | 0.005 | 0.005 | 0.005 | 0.005 | 0.005 | 0.005 | 0.005 | 0.005 | 0.005 | 0.004 |  |
| 28 | 0.004 | 0.004 | 0.004 | 0.004 | 0.004 | 0.004 | 0.004 | 0.005 | 0.004 | 0.004 | 0.004 | 0.004 | 0.004 | 0.004 | 0.004 | 0.004 | 0.004 | 0.004 | 0.004 | 0.004 |  |
| 29 | 0.002 | 0.002 | 0.002 | 0.002 | 0.002 | 0.002 | 0.002 | 0.002 | 0.002 | 0.002 | 0.002 | 0.002 | 0.002 | 0.002 | 0.002 | 0.002 | 0.002 | 0.002 | 0.002 | 0.002 |  |
| 30 | 0.005 | 0.005 | 0.005 | 0.005 | 0.004 | 0.005 | 0.005 | 0.004 | 0.004 | 0.004 | 0.004 | 0.005 | 0.004 | 0.004 | 0.004 | 0.004 | 0.004 | 0.005 | 0.005 | 0.005 |  |
| 31 | 0.004 | 0.004 | 0.004 | 0.004 | 0.004 | 0.004 | 0.004 | 0.004 | 0.004 | 0.004 | 0.004 | 0.004 | 0.004 | 0.004 | 0.004 | 0.004 | 0.004 | 0.004 | 0.004 | 0.004 |  |
| 32 | 0.003 | 0.003 | 0.003 | 0.004 | 0.004 | 0.004 | 0.004 | 0.004 | 0.004 | 0.004 | 0.004 | 0.004 | 0.003 | 0.004 | 0.004 | 0.004 | 0.004 | 0.004 | 0.004 | 0.003 |  |
| 33 | 0.002 | 0.002 | 0.002 | 0.002 | 0.002 | 0.002 | 0.002 | 0.002 | 0.002 | 0.002 | 0.002 | 0.002 | 0.002 | 0.002 | 0.002 | 0.002 | 0.002 | 0.002 | 0.002 | 0.002 |  |
| 34 | 0.005 | 0.005 | 0.005 | 0.005 | 0.005 | 0.005 | 0.005 | 0.005 | 0.005 | 0.005 | 0.005 | 0.005 | 0.005 | 0.005 | 0.005 | 0.005 | 0.005 | 0.005 | 0.005 | 0.005 |  |
| 35 | 0.008 | 0.008 | 0.007 | 0.008 | 0.008 | 0.007 | 0.008 | 0.008 | 0.008 | 0.009 | 0.008 | 0.008 | 0.007 | 0.007 | 0.008 | 0.008 | 0.008 | 0.008 | 0.008 | 0.008 |  |
| 36 | 0.008 | 0.008 | 0.008 | 0.008 | 0.008 | 0.008 | 0.007 | 0.007 | 0.007 | 0.008 | 0.007 | 0.007 | 0.007 | 0.007 | 0.007 | 0.007 | 0.007 | 0.008 | 0.007 | 0.007 |  |
| 37 | 0.006 | 0.006 | 0.006 | 0.006 | 0.006 | 0.006 | 0.005 | 0.005 | 0.005 | 0.005 | 0.006 | 0.005 | 0.006 | 0.006 | 0.005 | 0.006 | 0.005 | 0.005 | 0.005 | 0.005 |  |
| 38 | 0.005 | 0.005 | 0.005 | 0.005 | 0.005 | 0.005 | 0.005 | 0.005 | 0.005 | 0.006 | 0.005 | 0.005 | 0.005 | 0.005 | 0.005 | 0.005 | 0.005 | 0.005 | 0.005 | 0.005 |  |
| 39 | 0.005 | 0.005 | 0.004 | 0.004 | 0.004 | 0.005 | 0.006 | 0.005 | 0.005 | 0.006 | 0.005 | 0.005 | 0.006 | 0.007 | 0.007 | 0.006 | 0.007 | 0.007 | 0.007 | 0.007 |  |
| 40 | 0.006 | 0.006 | 0.005 | 0.005 | 0.005 | 0.005 | 0.006 | 0.006 | 0.006 | 0.006 | 0.005 | 0.006 | 0.006 | 0.007 | 0.007 | 0.007 | 0.007 | 0.007 | 0.007 | 0.007 |  |
| 41 | 0.005 | 0.005 | 0.005 | 0.005 | 0.005 | 0.005 | 0.005 | 0.005 | 0.006 | 0.006 | 0.006 | 0.005 | 0.005 | 0.005 | 0.004 | 0.004 | 0.004 | 0.004 | 0.004 | 0.004 |  |
| 42 | 0.007 | 0.007 | 0.007 | 0.007 | 0.007 | 0.007 | 0.007 | 0.007 | 0.008 | 0.009 | 0.009 | 0.009 | 0.009 | 0.009 | 0.009 | 0.009 | 0.009 | 0.009 | 0.009 | 0.009 |  |
| 43 | 0.005 | 0.005 | 0.005 | 0.005 | 0.006 | 0.005 | 0.005 | 0.006 | 0.006 | 0.007 | 0.007 | 0.006 | 0.007 | 0.007 | 0.007 | 0.006 | 0.007 | 0.006 | 0.006 | 0.007 |  |
| 44 | 0.005 | 0.005 | 0.005 | 0.005 | 0.005 | 0.005 | 0.005 | 0.005 | 0.006 | 0.005 | 0.006 | 0.006 | 0.005 | 0.006 | 0.005 | 0.005 | 0.007 | 0.007 | 0.007 | 0.007 |  |
| 45 | 0.003 | 0.003 | 0.003 | 0.003 | 0.003 | 0.003 | 0.004 | 0.004 | 0.004 | 0.004 | 0.004 | 0.004 | 0.004 | 0.004 | 0.004 | 0.004 | 0.004 | 0.005 | 0.005 | 0.005 |  |
| 46 | 0.009 | 0.010 | 0.008 | 0.010 | 0.011 | 0.008 | 0.010 | 0.010 | 0.008 | 0.008 | 0.009 | 0.011 | 0.010 | 0.010 | 0.009 | 0.010 | 0.009 | 0.009 | 0.009 | 0.008 |  |
| 49 | 0.005 | 0.006 | 0.006 | 0.006 | 0.007 | 0.008 | 0.008 | 0.008 | 0.008 | 0.008 | 0.008 | 0.008 | 0.007 | 0.007 | 0.007 | 0.007 | 0.007 | 0.007 | 0.007 | 0.007 |  |
| 50 | 0.010 | 0.011 | 0.011 | 0.011 | 0.012 | 0.013 | 0.013 | 0.013 | 0.013 | 0.014 | 0.013 | 0.014 | 0.013 | 0.013 | 0.013 | 0.013 | 0.013 | 0.013 | 0.013 | 0.013 |  |
| 51 | 0.012 | 0.014 | 0.015 | 0.015 | 0.017 | 0.017 | 0.017 | 0.018 | 0.017 | 0.018 | 0.017 | 0.017 | 0.017 | 0.017 | 0.017 | 0.017 | 0.017 | 0.017 | 0.017 | 0.017 |  |
| 52 | 0.012 | 0.013 | 0.015 | 0.016 | 0.017 | 0.017 | 0.018 | 0.018 | 0.018 | 0.018 | 0.018 | 0.018 | 0.019 | 0.019 | 0.019 | 0.019 | 0.019 | 0.019 | 0.019 | 0.019 |  |
| 53 | 0.016 | 0.017 | 0.017 | 0.017 | 0.018 | 0.018 | 0.020 | 0.019 | 0.018 | 0.018 | 0.018 | 0.018 | 0.020 | 0.020 | 0.020 | 0.020 | 0.021 | 0.020 | 0.019 | 0.020 |  |
| 59 | 0.017 | 0.016 | 0.020 | 0.021 | 0.020 | 0.018 | 0.017 | 0.017 | 0.017 | 0.016 | 0.017 | 0.016 | 0.017 | 0.018 | 0.017 | 0.017 | 0.016 | 0.017 | 0.020 | 0.020 |  |
| 60 | 0.013 | 0.013 | 0.012 | 0.014 | 0.013 | 0.013 | 0.013 | 0.012 | 0.013 | 0.013 | 0.012 | 0.013 | 0.013 | 0.013 | 0.013 | 0.013 | 0.013 | 0.012 | 0.012 | 0.012 |  |
| 62 | 0.005 | 0.006 | 0.006 | 0.006 | 0.006 | 0.005 | 0.005 | 0.005 | 0.005 | 0.005 | 0.005 | 0.005 | 0.005 | 0.005 | 0.005 | 0.005 | 0.005 | 0.005 | 0.005 | 0.005 |  |
| 65 | 0.024 | 0.024 | 0.025 | 0.024 | 0.025 | 0.030 | 0.030 | 0.029 | 0.030 | 0.028 | 0.030 | 0.029 | 0.028 | 0.031 | 0.030 | 0.030 | 0.029 | 0.028 | 0.029 | 0.029 |  |
| 66 | 0.024 | 0.028 | 0.026 | 0.023 | 0.025 | 0.026 | 0.024 | 0.022 | 0.022 | 0.021 | 0.022 | 0.025 | 0.024 | 0.025 | 0.029 | 0.029 | 0.031 | 0.033 | 0.031 | 0.031 |  |
