# Supplementary material for: Global change in the trophic functioning of marine food webs
Source: PLoS One. 2017 Aug 11;12(8):e0182826. doi: 10.1371/journal.pone.0182826 (PMC5553640; doi:10.1371/journal.pone.0182826)
Supplement: S1 Appendix — The 66 LMEs are represented with the corresponding official names (from www.lme.noaa.gov). The 10 ecosystems that include the highest fraction of overexploited and collapsed stocks are represented in red (Group 1). The 10 ecosystems that demonstrate the highest increase in sea surface temperature between 1990 and 2010 are represented in orange (Group 2). The 10 LMEs excluded from the database are listed as ‘not included’. (DOCX) [file pone.0182826.s003.docx]

S1 Appendix. Large Marine Ecosystems (LMEs) map and characteristics.


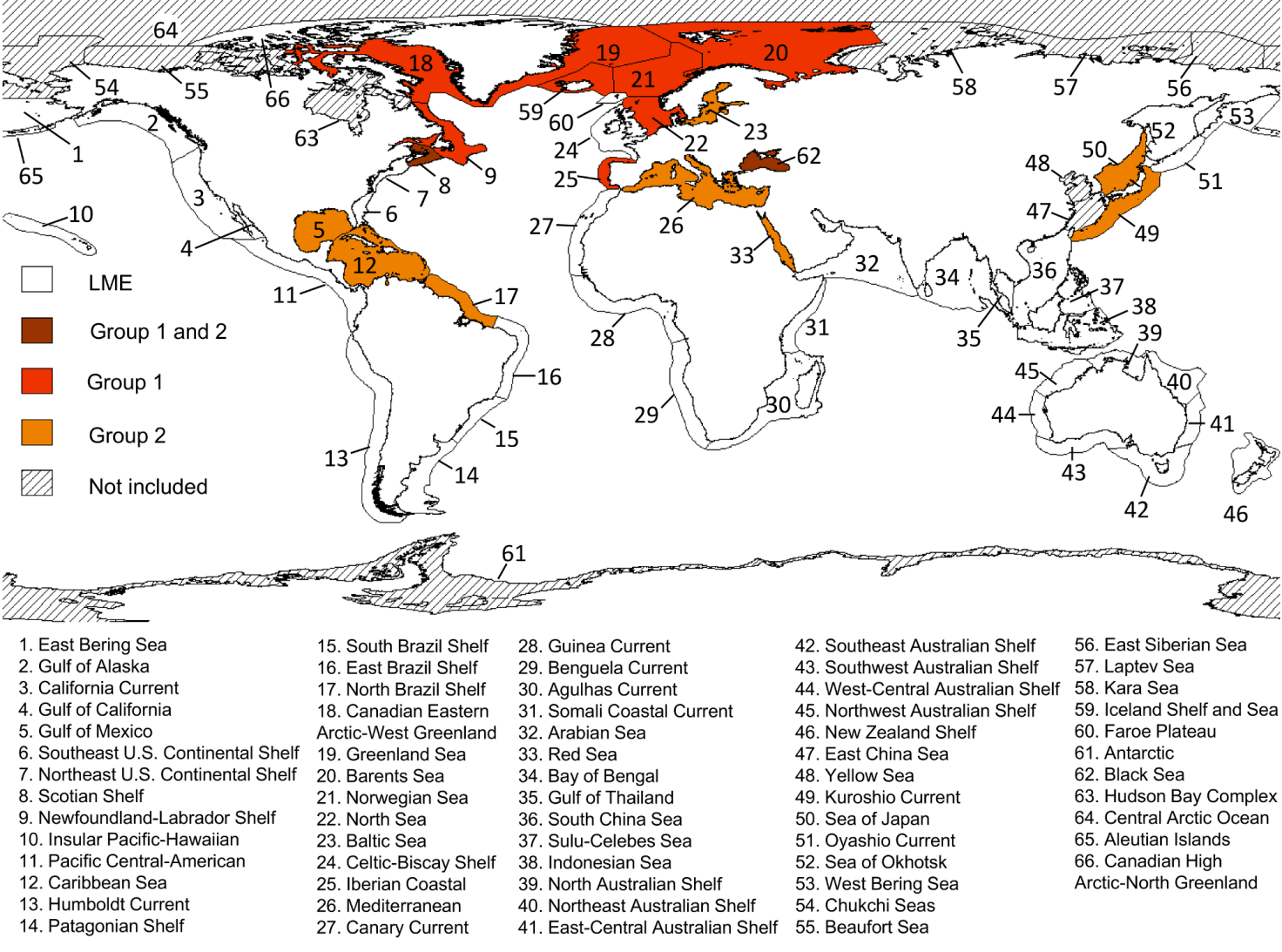


The 66 LMEs are represented with the corresponding official names (from [www.lme.noaa.gov](http://www.lme.noaa.gov)). The 10 ecosystems that include the highest fraction of overexploited and collapsed stocks are represented in red (Group 1). The 10 ecosystems that demonstrate the highest increase in sea surface temperature between 1980-1985 and 2005-2010 are represented in orange (Group 2). The 10 LMEs excluded from the database are listed as ‘not included’.

1. East Bering Sea
2. Gulf of Alaska
3. California Current
4. Gulf of California
5. Gulf of Mexico
6. Southeast U.S. Continental Shelf
7. Northeast U.S. Continental Shelf
8. Scotian Shelf
9. Newfoundland-Labrador Shelf
10. Insular Pacific-Hawaiian
11. Pacific Central-American
12. Caribbean Sea
13. Humboldt Current
14. Patagonian Shelf
15. South Brazil Shelf
16. East Brazil Shelf
17. North Brazil Shelf
18. Canadian Eastern Arctic-West Greenland
19. Greenland Sea
20. Barents Sea
21. Norwegian Sea
22. North Sea
23. Baltic Sea
24. Celtic-Biscay Shelf
25. Iberian Coastal
26. Mediterranean
27. Canary Current
28. Guinea Current
29. Benguela Current
30. Agulhas Current
31. Somali Coastal Current
32. Arabian Sea
33. Red Sea
34. Bay of Bengal
35. Gulf of Thailand
36. South China Sea
37. Sulu-Celebes Sea
38. Indonesian Sea
39. North Australian Shelf
40. Northeast Australian Shelf
41. East-Central Australian Shelf
42. Southeast Australian Shelf
43. Southwest Australian Shelf
44. West-Central Australian Shelf
45. Northwest Australian Shelf
46. New Zealand Shelf
47. East China Sea
48. Yellow Sea
49. Kuroshio Current
50. Sea of Japan
51. Oyashio Current
52. Sea of Okhotsk
53. West Bering Sea
54. Chukchi Seas
55. Beaufort Sea
56. East Siberian Sea
57. Laptev Sea
58. Kara Sea
59. Iceland Shelf and Sea
60. Faroe Plateau
61. Antarctic
62. Black Sea
63. Hudson Bay Complex
64. Central Arctic Ocean
65. Aleutian Islands
66. Canadian High Artic-North Greenland
