# Supplementary material for: Global change in the trophic functioning of marine food webs
Source: PLoS One. 2017 Aug 11;12(8):e0182826. doi: 10.1371/journal.pone.0182826 (PMC5553640; doi:10.1371/journal.pone.0182826)
Supplement: S2 Appendix — (DOCX) [file pone.0182826.s004.docx]

**S2 Appendix. Sensitivity Analysis based on an Ecopath model for the North Sea.**

**
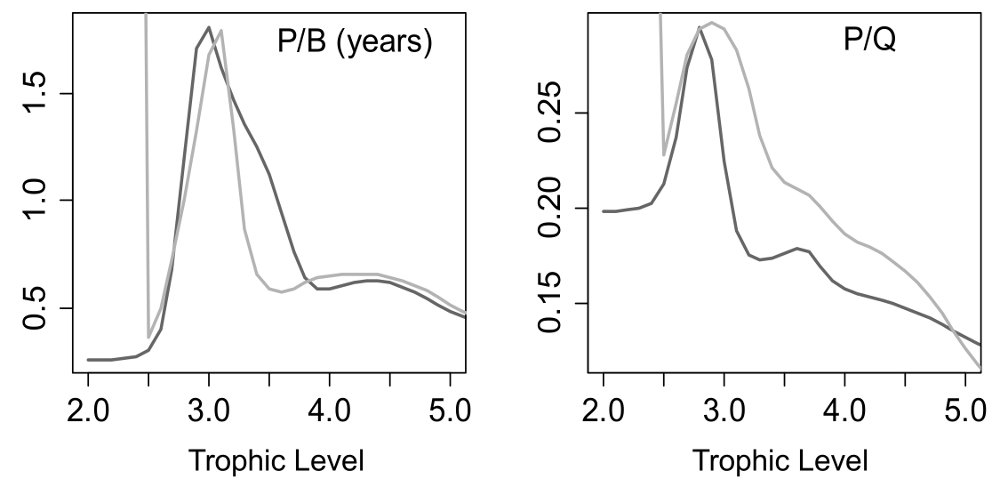
**

Confrontation of trophic spectra constructed with biomass or catch data for the North Sea.

Left: speed of flows P/B trophic spectrum constructed from catch data in dark grey and from biomass data in light grey. Right: partial trophic transfer efficiency P/Q spectrum from catch/biomass data – using Ecopath model from 1991 for the North Sea [1].

Values of indicators ECI and TCI using catch data or biomass for calculation in 1991 for the North Sea.

| **Sources of data** | **Gross value of TCI** | **Gross value of ECI** | **ECI per trophic level** |
| --- | --- | --- | --- |
| **Catch** | 4.0 years | 0.03 | 18.4 % |
| **Biomass** | 2.3 years | 0.07 | 26.0 % |

**Literature**

1. Mackinson S, Daskalov G. An ecosystem model of the North Sea to support an ecosystem approach to fisheries management: description and parameterisation. Cefas Lowestoft; 2007 p. 196pp. (Science Series Technical Report). Report No.: 142.
