# Supplementary material for: Global change in the trophic functioning of marine food webs
Source: PLoS One. 2017 Aug 11;12(8):e0182826. doi: 10.1371/journal.pone.0182826 (PMC5553640; doi:10.1371/journal.pone.0182826)
Supplement: S3 Appendix — The trends for each cluster and indicator were plotted taking into account finfish species only, cumulating indicators from TL = 2.5 to TL = 4.0. (DOCX) [file pone.0182826.s005.docx]

**S3 Appendix. Sensitivity of the results to non-fish species’ influence on the clusters trends for the functioning indicators TCI and ECI.**

The trends for each cluster and indicator were plotted taking into account finfish species only, cumulating indicators from TL=2.5 to TL=4.0:


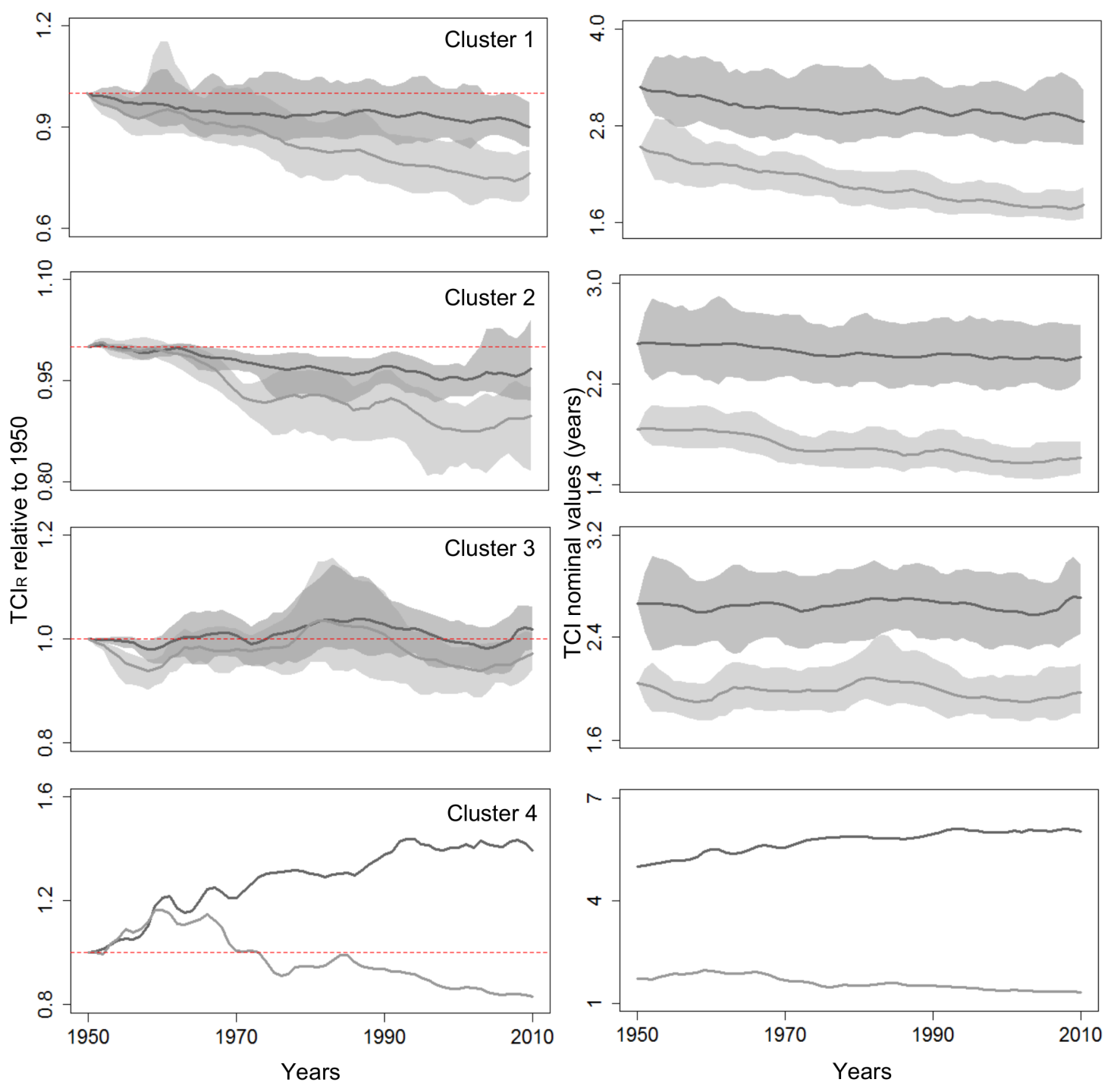


Graphs on the left indicate the mean TCI_R_ trend per cluster with all species (light grey) and only finfish species (dark grey), with bootstrap confidence intervals at 95%. Graphs on the right are TCI nominal values for all species (light grey) and only finfish species (dark grey).


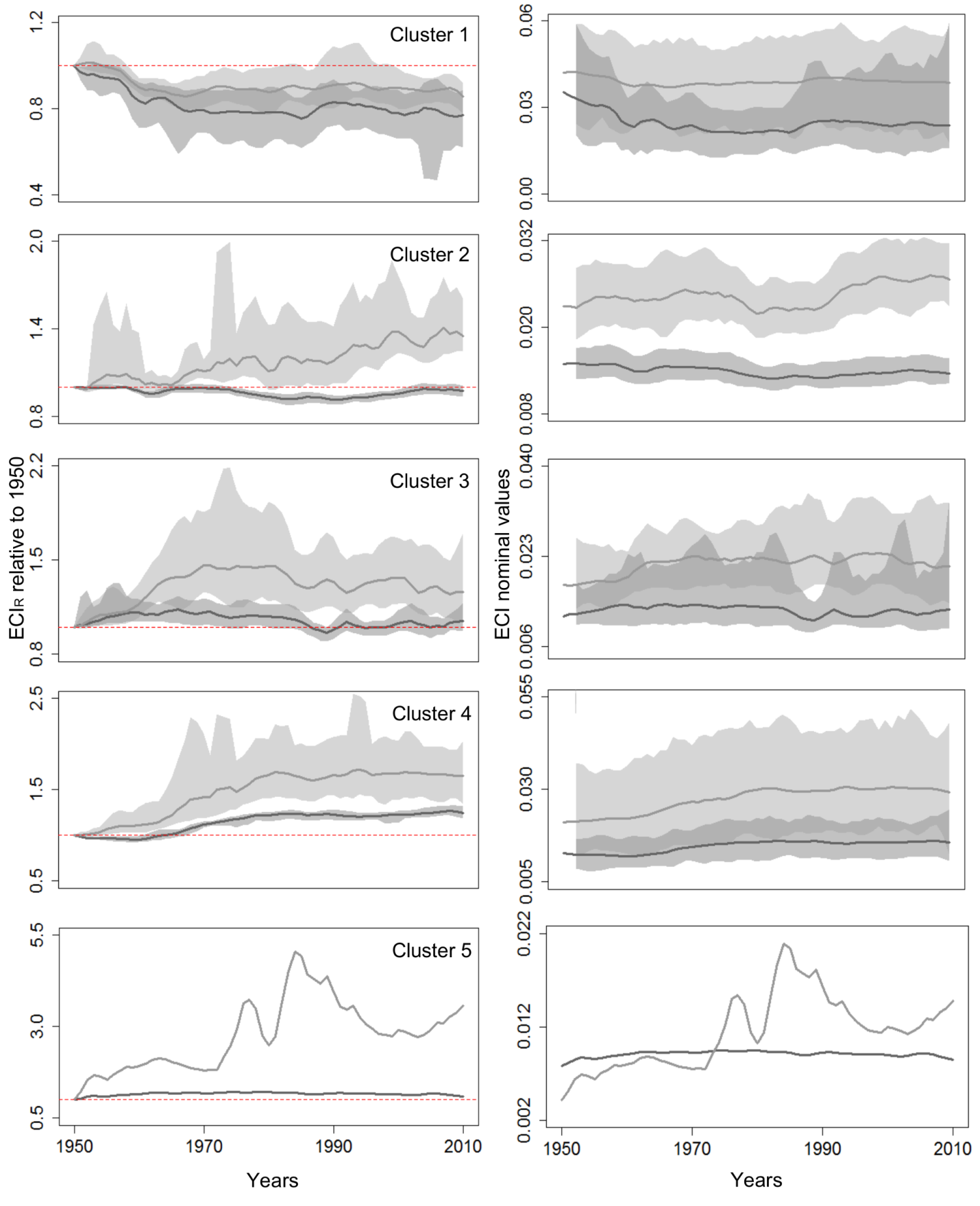


Graphs on the left indicate the mean ECI_R_ trend per cluster with all species (light grey) and only finfish species (dark grey), with bootstrap confidence intervals at 95%. Graphs on the right are ECI nominal values for all species (light grey) and only finfish species (dark grey).
