# Supplementary material for: Global change in the trophic functioning of marine food webs
Source: PLoS One. 2017 Aug 11;12(8):e0182826. doi: 10.1371/journal.pone.0182826 (PMC5553640; doi:10.1371/journal.pone.0182826)
Supplement: S4 Appendix — (DOCX) [file pone.0182826.s006.docx]

**S4 Appendix. Case studies and species assemblages.**

For a sub-set of 6 LMEs (Scotian Shelf, Humboldt Current, North Sea, Gulf of Thailand, Kuroshio Current and Aleutian Islands), change in species assemblages were studied using partial indicators TCI_R_ and ECI_R_ cumulated progressively until TL=2.5/3.0/3.5/4.0/4.5, thus searching at which TLs changes in TCI_R_ or ECI_R_ occurred, and analyzing species parameters among the different trophic class. For each trophic class, the taxonomic groups parameters and catch abundance were analyzed along the indicators variations in order to relate variations in TCI_R_, ECI_R_ and species responsible for such variations. For instance, if in one ecosystem the indicator TCI_R_ (or ECI_R_) decreased in the trophic class 3.0-3.5, we looked for the species at corresponding trophic level with a high P/B ratio (or low P/Q ratio) and whose abundance in the catch increased. Thus, this analysis was using the progressively cumulated indicators along the trophic class, the species catch abundance per trophic class since 1950 and the species corresponding parameters.

There was a great variability in the trophic functioning of the six ecosystems used as case studies. Indicators computed for increasing ranges of TLs provided a more comprehensive understanding of changes that have occurred since 1950.


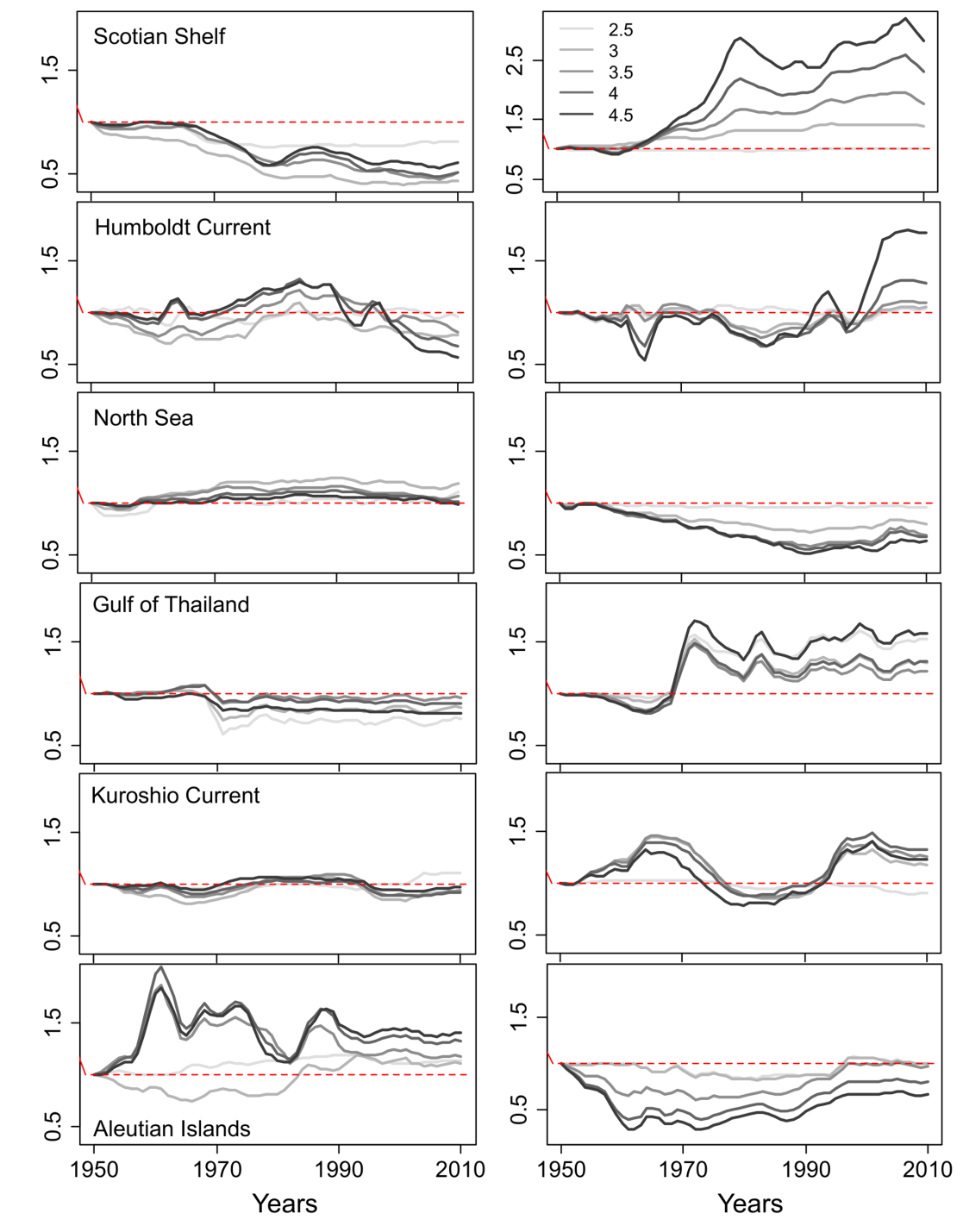


Fig. Time and efficiency cumulated indicators, computed between TL=2.0 and TL=b (for increasing b, from 2.5 to 4.5), in six LMEs selected as case studies. Left: relative TCI_R_ to 1950; Right: relative ECI_R_ to 1950

Scotian Shelf ecosystem (TCI_R_ cluster 1, ECI_R_ cluster 4) was characterized by a significant drop in the fisheries catch from more than 2.2 million of tons in the 60s to 300,000 tons in the recent period. The 70s were marked by the strong decrease in catch and by a large change in the species composition explaining the trends observed in both indicators, especially for trophic levels higher than 3.0. The biomass flow properties were no longer determined by the Gulf menhaden (TL=3.2)*, Sebastes* genus (TL=3.8), Winter flounder (TL=3.6)*,* Atlantic cod (TL=4.1)*, Merluccius* genus but rather by species with higher conversion efficiency and shorter residence time: Snow crab (TL=2.3), Atlantic rock crab (TL=2.6), Northern shrimp (TL=3.1), the American lobster (TL=3.7) and the Atlantic mackerel (TL=3.63). Such kind of change towards lower trophic levels and invertebrates clearly reflected a strong overfishing of predator fishes and an overall fishing down the food web process.

The Humboldt Current LME (TCI_R_ cluster 3, ECI_R_ cluster 2) demonstrated very different trends, in part due to the transition between small pelagic species. When the Peruvian Anchoveta (*Engraulis ringens*, TL=2.7) was abundant, the ECI_R_ increased and the TCI_R_ decreased since this species is two times more efficient than the South American Pilchard (*Sardinops sagax*, TL=2.8). The drop in ECI_R_ concordant with the peak in TCI_R_ during the 80s-90s was due to the abundance of pilchard. From the trophic class 3.0-3.5, the increasing presence of the Chilean Jack Mackerel (TL=3.3) induces a loss of trophic efficiency and longer time of transfers. Starting in the 2000s, an increase in ECI_R_ and a decrease in TCI_R_ were observed, due to the emergence of the Chilean silverside (TL=4.0), the Southern blue whiting (TL=3.8), the Jumbo flying squid (TL=4.1) and the Common dolphinfish (TL=4.4). Such an evolution thus reflected the environment-induced alternations between two small pelagics and the recent expansion of fisheries towards high trophic levels.

Concerning the North Sea (TCI_R_ cluster 3, ECI_R_ cluster 1), a significant decrease in the trophic efficiency indicator (by 30-40% since 1950) and a slight increase in TCI_R_ were highlighted. The higher proportion of the European sprat (TL=3.0) might have induced longer times of transfer at rather low trophic levels. The clear replacement of the Atlantic herring (TL=3.4) by Sand eels (*Ammodytes*, TL=3.1) and Sand lances (*Ammodytidae*, TL=3.1) induced faster and less efficient transfers in the ecosystem. At higher trophic level the emergence of the Atlantic horse mackerel (TL=3.7) in the catch since the 80s-90s induced even less efficient and faster transfers Overexploitation of the Atlantic cod and other *Gadidae* reinforced these trends, because the remaining species are less efficient and generate faster transfers: the Atlantic bonito (TL=4.5) and the Blue whiting (TL=4.1).

For the tropical Gulf of Thailand ecosystem (TCI_R_ cluster 2, ECI_R_ cluster 2), the observed change is partly due to the development of the cnidarians fisheries (*Cnidaria*, TL=2.5), which induced a sudden increase in ECI_R_ and decrease in TCI_R_ in the early 70s. The decrease of the *Mugilidae* species was accompanied by a replacement by shrimps species and Sardinellas (TL=2.8), more efficient in trophic transfers, while tonguefishes induced slower transfers (*Cynoglossidae*, TL=3.2), just as Breams (*Nemipteridae*, TL=3.5). However, the increasing proportion of Jacks (*Carangidae*, TL=4.0) and Lizardfishes (*Synodontidae*, TL=4.3) induced another increase of ECI_R_ and decrease in TCI_R_ compared to the 50s when the King Mackerel was more abundant (TL=4.3). Overall, the various effects offset each other for the TCI_R_ indicator, thus inducing almost no change in the time cumulated indicator between trophic levels 2 and 4, while in contrast trophic transfers appeared more efficient.

The Kuroshio Current (TCI_R_ cluster 2, ECI_R_ cluster 2) was driven by species in the trophic class 2.5-3.0 and the Pacific Sardine (*Sardinops sagax*, TL=2.8) leads the observed variations. The species was abundant mainly during the mid-70s to the 90s and explained the drop in ECI_R_ at that period, since the Pacific Sandlance (TL=3.0) and Akaimi paste shrimp (TL=2.7) in the same class are more efficient. However, the residence time of the Sandlance is comparable to the sardine, explaining slighter and opposite variations on the TCI_R_.

In the Aleutian Islands (TCI_R_ cluster 4, ECI_R_ cluster 1), there was a progressive loss of trophic efficiency over the first decade, reflecting changes which jointly occurred at all trophic levels, but mainly driven by the development of fisheries at the beginning of the time-series. The indicators started to increase in the late 80s mainly due to a higher proportions of the Alaska Pollock (TL=3.6) and the Sockeye salmon (TL=3.5). The strong increase and difference between trophic class 2.5-3.0 and 3.0-3.5 for the TCI_R_ was due to the group *Pleuronectidae* (TL=3.5) characterized by slow transfers, but the Pacific herring (TL=3.2) and the Yellowfin sole (TL=3.5) were enhancing the decrease after the 80s.

Table. Ratios of interest (P/B, P/Q) for the main species in 6 LMEs (Scotian Shelf, Humboldt Current, North Sea, Gulf of Thailand, Kuroshio Current and Aleutian Islands).

| **Ecosystem** | **Trophic class** | **Scientific name** | **Common name** | **TL** | **P/B ratio** | **P/Q ratio** |
| --- | --- | --- | --- | --- | --- | --- |
| **Scotian Shelf** | [2.0 ; 2.5[ | Placopecten magellanicus  Spisula solidissima  Mercenaria mercenaria  Chionoecetes opilio | Scallop shell  Atlantic surf clam  Hard clam  Snow crab | 2.0  2.0  2.0  2.3 | 1.79  1.79  1.79  1.88 | 0.19  0.19  0.19  0.11 |
|  | [2.5 ; 3.0[ | Pandalus  Cancer irroratus | Shrimps  Atlantic rock crab | 2.6  2.6 | 3.33  1.88 | 0.21  0.22 |
|  | [3.0 ; 3.5[ | Brevoortia patronus  Clupea harengus  Pandalus borealis | Gulf menhaden  Atlantic herring  Northern shrimp | 3.2  3.4  3.1 | 0.59  0.50  3.33 | 0.09  0.11  0.21 |
|  | [3.5 ; 4.0[ | Sebastes  Rajidae  Homarus americanus  Myoxocephalus | Redfishes  Stingrays  American lobster  Grubbies and sculpins | 3.8  3.8  3.7  3.7 | 0.21  0.26  1.03  0.32 | 0.03  0.04  0.14  0.02 |
|  | [4.0 ; 4.5 [ | Merluccius  Isurus oxyrinchus  Gadus morhua | Hake  Shortfin mako  Atlantic cod | 4.3  4.5  4.1 | 0.26  0.30  0.30 | 0.09  0.21  0.15 |
| **Humboldt Current** | [2.0 ; 2.5[ | Clupea bentincki  Aulacomya ater  Argopecten purpuratus  Mugil cephalus  Ethmidium maculatum | Araucanian herring  Chilean ribbed mussel  Peruvian calico scallop  Flathead grey mullet  Pacific menhaden | 2.0  2.0  2.0  2.1  2.1 | 0.61  3.10  1.79  0.49  3.06 | 0.03  0.28  0.19  0.03  0.03 |
|  | [2.5 ; 3.0[ | Engraulis ringens  Sardinops sagax | Anchoveta  Pacific sardine | 2.7  2.8 | 1.92  0.73 | 0.08  0.04 |
|  | [3.0 ; 3.5[ | Trachurus murphyi  Scomber japonicus  Marine fishes not id. | Chilean jack mackerel  Chub mackerel  Marine fishes | 3.3  3.4  3.3 | 0.25  0.55  0.42 | 0.06  0.09  0.07 |
|  | [3.5 ; 4.0[ | Anguilliformes  Macruronus magellanicus  Odontesthes regia  Micromesistius australis | Eels morays  Patagonian grenadier  Chilean silverside  Southern blue whiting | 3.9  3.9  4.0  3.8 | 0.24  0.35  0.82  0.51 | 0.03  0.08  0.18  0.10 |
|  | [4.0 ; 4.5 [ | Merluccius  Merluccius gayi gayi  Merluccius australis  Sarda chiliensis  Dosidicus gigas  Coryphaena hippurus | Hakes  South Pacific hake  Southern hake  Eastern Pacific bonito  Jumbo flying squid  Common dolphinfish | 4.3  4.3  4.5  4.5  4.1  4.4 | 0.35  0.44  0.41  0.51  3.04  1.68 | 0.11  0.14  0.14  0.08  0.23  0.65 |
| **North Sea** | [2.0 ; 2.5[ | Cardium edule  Miscellaneous aquatic inv.  Mytilus edulis | Common cockle  Aquatic inv.  Blue mussel | 2.1  2.4  2.0 | 1.79  1.84  3.1 | 0.19  0.19  0.28 |
|  | [2.5 ; 3.0[ | Sprattus sprattus  Nephrops norvegicus  Cancer pagurus | European sprat  Norway lobster  Edible crab | 3.0  2.9  2.6 | 0.90  1.03  1.88 | 0.13  0.14  0.22 |
|  | [3.0 ; 3.5[ | Clupea harengus  Ammodytes  Ammodytidae  Limanda limanda  Pleuronectes platessa | Atlantic herring  Sand eels  Sand lances  Common dab  European plaice | 3.4  3.1  3.1  3.3  3.3 | 0.56  0.81  0.79  0.55  0.34 | 0.11  0.11  0.05  0.18  0.11 |
|  | [3.5 ; 4.0[ | Scomber scombrus  Gadidae  Trachurus trachurus | Atlantic mackerel  Cods, haddocks  Atl. Horse mackerel | 3.6  3.8  3.7 | 0.54  0.43  0.32 | 0.11  0.07  0.07 |
|  | [4.0 ; 4.5 [ | Gadus morhua  Melanogrammus aeglefinus  Merlangius merlangus  Pollachius virens  Sarda sarda  Micromesistius poutassou | Atlantic cod  Haddock  Whiting  Saithe  Atlantic bonito  Blue whiting | 4.1  4.0  4.4  4.3  4.5  4.1 | 0.41  0.39  0.51  0.28  0.75  0.31 | 0.23  0.16  0.17  0.13  0.13  0.09 |
| **Gulf of Thailand** | [2.0 ; 2.5[ | Perna viridis  Veneridae  Cnidaria | Green mussel  Shells  Cnidarians | 2.0  2.0  2.5 | 3.10  1.79  1.12 | 0.28  0.19  0.39 |
|  | [2.5 ; 3.0[ | Mugilidae  Sardinella  Penaeus | Mullets  Sardinella  Shrimps | 2.5  2.8  2.7 | 0.90  1.70  3.33 | 0.05  0.05  0.21 |
|  | [3.0 ; 3.5[ | Rastrelliger kanagurta  Leiognathidae  Engraulidae  Cynoglossidae | Indian mackerel  [Slimys, slipmouths](http://www.fishbase.org/summary/FamilySummary.php?ID=318)  Anchovies  Tonguefishes | 3.2  3.2  3.3  3.3 | 1.82  1.87  2.34  0.70 | 0.09  0.06  0.05  0.03 |
|  | [3.5 ; 4.0[ | Loliginidae  Loligo  Decapterus russelli  Nemipteridae  Sciaenidae  Sepiidae | Common pencil squids  Common squids  Indian scad  Breams  Drums or croakers  Cuttlefishes | 3.9  3.9  3.7  3.5  3.8  3.6 | 3.05  3.05  1.38  1.02  1.14  2.17 | 0.23  0.23  0.08  0.09  0.05  0.31 |
|  | [4.0 ; 4.5 [ | Carangidae  Scomberomorus guttatus  Synodontidae | Jacks and pompanos  Indo-Pacific king mackerel  Lizardfishes | 4.1  4.3  4.3 | 1.52  0.49  1.18 | 0.05  0.05  0.12 |
| **Kuroshio Current** | [2.0 ; 2.5[ | Bivalvia  Pectinidae  Miscellaneous marine mol.  Mugil cephalus | Clams  Scallops  Marine molluscs  Flathead grey mullet | 2.2  2.0  2.3  2.1 | 1.79  1.79  1.84  0.55 | 0.19  0.19  0.19  0.02 |
|  | [2.5 ; 3.0[ | Sardinops sagax  Ammodytes personatus  Acetes japonicus | Pacific sardine  Pacific sandlance  Akiami paste shrimp | 2.8  3.0  2.7 | 0.97  0.94  3.33 | 0.04  0.07  0.21 |
|  | [3.0 ; 3.5[ | Pleuronectidae  Marine fishes not identified  Trachurus japonicus  Engraulis japonicus  Crustacea | Righteye flounders  Marine fishes  Japanese jack mackerel  Japanese anchovy | 3.5  3.3  3.4  3.1  3.2 | 0.32  0.35  0.64  1.67  2.84 | 0.02  0.05  0.08  0.06  0.21 |
|  | [3.5 ; 4.0[ | Colobis saira  Oncorhynchus  Scomber  Theragra chalcogramma | Pacific saury  Salmons trouts  Chub mackerels  Alaska pollock | 3.7  3.9  3.7  3.6 | 0.89  0.82  0.92  0.49 | 0.10  0.07  0.06  0.09 |
|  | [4.0 ; 4.5 [ | Carangidae  Gadus macrocephalus  Katsuwonus pelamis  Todarodes pacificus  Trichiurus lepturus | Jacks pompanos  Pacific cod  Skipjack tuna  Japanese flying squid  Largehead hairtail | 4.1  4.2  4.4  4.3  4.4 | 0.91  0.40  1.01  3.04  0.57 | 0.05  0.11  0.09  0.23  0.09 |
| **Aleutian Islands** | [2.0 ; 2.5[ | Chionoecetes | Crabs | 2.3 | 1.88 | 0.22 |
|  | [2.5 ; 3.0[ | Pandalidae  Paralithodes camtschaticus | Shrimps  Red king crab | 2.7  2.8 | 3.33  1.88 | 0.21  0.22 |
|  | [3.0 ; 3.5[ | Marine fishes not identified  Pleuronectidae  Limanda aspera  Lepidopsetta bilineata  Clupea pallasii pallasii | Marine fishes  Righteye flounders  Yellowfin sole  Rock sole  Pacific herring | 3.3  3.5  3.5  3.2  3.2 | 0.17  0.10  0.32  0.10  0.49 | 0.08  0.06  0.18  0.13  0.20 |
|  | [3.5 ; 4.0[ | Theragra chalcogramma  Pleuronectiformes  Oncorhynchus nerka | Alaska Pollock  Flatfishes  Sockeye salmon | 3.6  3.6  3.5 | 0.32  0.10  0.54 | 0.23  0.06  0.32 |
|  | [4.0 ; 4.5 [ | Oncorhynchus gorbuscha  Gadus macrocephalus | Pink salmon  Pacific cod | 4.5  4.2 | 0.54  0.20 | 0.32  0.24 |
