# Supplementary material for: Global change in the trophic functioning of marine food webs
Source: PLoS One. 2017 Aug 11;12(8):e0182826. doi: 10.1371/journal.pone.0182826 (PMC5553640; doi:10.1371/journal.pone.0182826)
Supplement: S5 Appendix — (DOCX) [file pone.0182826.s007.docx]

**S5 Appendix. Complementary statistical analysis on worldwide indicator trends.**

We tested the effect of time-series on the worldwide trends to detect if there is a significant change in the values of the indicators since 1950. Based on the Figure 3 c-d, we expect that the change in TCI since 1950 is an effective increase with a statistically significant change. Concerning ECI, the increase after the 1960s is less clear due to heterogeneity in the ecosystems and a higher variability in the global response.

Methods: Linear regression using ‘years’ as an explanatory qualitative variable and ‘ECI’ or ‘TCI’ as the variable to be explained by the statistical model. For the model, we used all the 56 time-series for each indicator and performed a model per indicator independently. In the model, we tested the following hypothesis:

H0: “The qualitative variable ‘years’ does not have an effect on the indicator trend”

Each year from 1951 to 2010 is compared to the first year 1950 and test the significance of the difference in indicator value. The p. values given by the model accept or reject that hypothesis. A small p. value will reject the hypothesis and confirm that the ‘years’ has a statistically significant effect on the trend. Then, the analysis of variance summarizes the significance of the qualitative variable. Data is standardized by the mean and the standard deviation.

Time Cumulated Indicator global trend:

1. *Data*


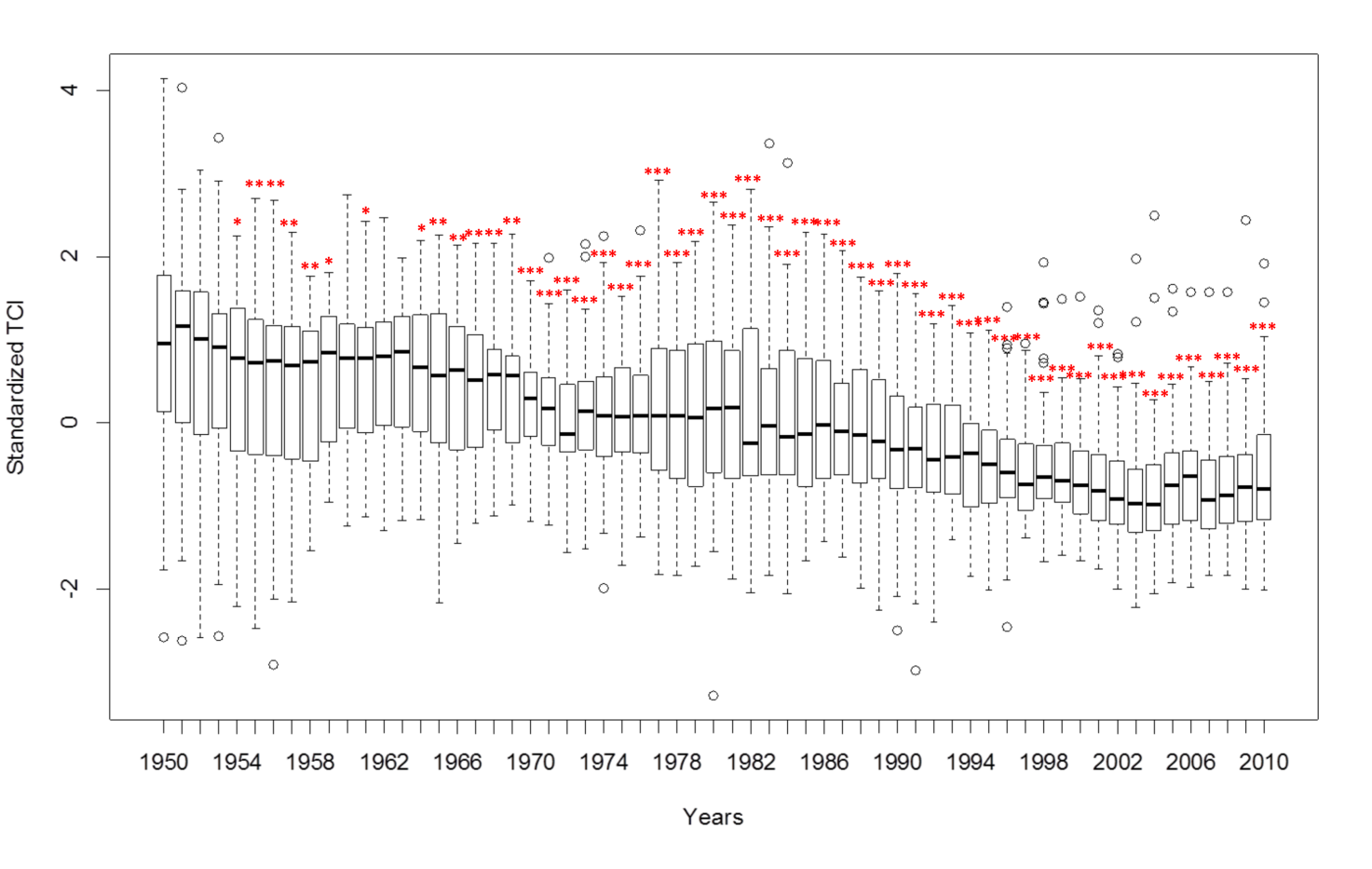


1. *Test of factor ‘years’ through a variance analysis*

|  | **Degrees of freedom** | **Sum Sq** | **Mean Sq** | **F value** | **Pr (>F)** |
| --- | --- | --- | --- | --- | --- |
| Factor ‘years’ | 60 | 827.59 | 13.7932 | 18.273 | < 2.20.10^-16^ *** |
| Residuals | 3355 | 2532.41 | 0.7548 |  |  |

Significance codes: 0 ‘***’ 0.001 ‘**’ 0.01 ‘*’ 0.05 ‘.’ 0.1 ‘’ 1

1. *Interpretation of coefficients*

Model formula: lm(TCI ~ years)

| **Factor** | **Estimate** | **Std. Error** | **t value** | **Pr (>\|t\|)** |
| --- | --- | --- | --- | --- |
| Intercept | 0.88 | 0.12 | 7.61 | 3.45.10^-14^ *** |
| 1951 | -0.02 | 0.16 | -0.11 | 0.913 |
| 1952 | -0.11 | 0.16 | -0.66 | 0.509 |
| 1953 | -0.22 | 0.16 | -1.37 | 0.170 |
| 1954 | -0.41 | 0.16 | -2.52 | 0.012 * |
| 1955 | -0.47 | 0.16 | -2.89 | 0.004 ** |
| 1956 | -0.52 | 0.16 | -3.19 | 0.001 ** |
| 1957 | -0.48 | 0.16 | -2.91 | 0.004 ** |
| 1958 | -0.46 | 0.16 | -2.79 | 0.005 ** |
| 1959 | -0.34 | 0.16 | -2.06 | 0.040 * |
| 1960 | -0.30 | 0.16 | -1.82 | 0.068 . |
| 1961 | -0.34 | 0.16 | -2.08 | 0.038 * |
| 1962 | -0.30 | 0.16 | -1.83 | 0.068 . |
| 1963 | -0.29 | 0.16 | -1.79 | 0.074 . |
| 1964 | -0.36 | 0.16 | -2.22 | 0.026 * |
| 1965 | -0.48 | 0.16 | -2.90 | 0.004 ** |
| 1966 | -0.48 | 0.16 | -2.90 | 0.004 ** |
| 1967 | -0.49 | 0.16 | -3.00 | 0.003 ** |
| 1968 | -0.47 | 0.16 | -2.88 | 0.004 ** |
| 1969 | -0.50 | 0.16 | -3.11 | 0.002 ** |
| 1970 | -0.67 | 0.16 | -4.10 | 4.25.10^-5^ *** |
| 1971 | -0.73 | 0.16 | -4.42 | 1.04.10^-5^ *** |
| 1972 | -0.84 | 0.16 | -5.11 | 3.49.10^-7^ *** |
| 1973 | -0.76 | 0.16 | -4.62 | 3.97.10^-6^ *** |
| 1974 | -0.80 | 0.16 | -4.86 | 1.26.10^-6^ *** |
| 1975 | -0.81 | 0.16 | -4.92 | 9.02.10^-7^ *** |
| 1976 | -0.79 | 0.16 | -4.78 | 1.80.10^-6^ *** |
| 1977 | -0.74 | 0.16 | -4.49 | 7.46.10^-6^ *** |
| 1978 | -0.75 | 0.16 | -4.60 | 4.48.10^-6^ *** |
| 1979 | -0.74 | 0.16 | -4.53 | 6.20.10^-6^ *** |
| 1980 | -0.73 | 0.16 | -4.47 | 8.24.10^-6^ *** |
| 1981 | -0.73 | 0.16 | -4.46 | 8.68.10^-6^ *** |
| 1982 | -0.71 | 0.16 | -4.35 | 1.38.10^-5^ *** |
| 1983 | -0.72 | 0.16 | -4.39 | 1.18.10^-5^ *** |
| 1984 | -0.73 | 0.16 | -4.45 | 8.86.10^-6^ *** |
| 1985 | -0.84 | 0.16 | -5.13 | 3.08.10^-7^ *** |
| 1986 | -0.79 | 0.16 | -4.79 | 1.75.10^-6^ *** |
| 1987 | -0.89 | 0.16 | -5.43 | 6.08.10^-8^ *** |
| 1988 | -0.93 | 0.16 | -5.67 | 1.52.10^-8^ *** |
| 1989 | -1.03 | 0.16 | -6.28 | 3.74.10^-10^ *** |
| 1990 | -1.08 | 0.16 | -6.57 | 5.70.10^-11^ *** |
| 1991 | -1.12 | 0.16 | -6.84 | 9.26.10^-12^ *** |
| 1992 | -1.23 | 0.16 | -7.50 | 8.17.10^-14^ *** |
| 1993 | -1.18 | 0.16 | -7.21 | 6.87.10^-13^ *** |
| 1994 | -1.31 | 0.16 | -9.87 | 2.00.10^-15^ *** |
| 1995 | -1.43 | 0.16 | -8.70 | < 2.00.10^-16^ *** |
| 1996 | -1.40 | 0.16 | -8.52 | < 2.00.10^-16^ *** |
| 1997 | -1.45 | 0.16 | -8.86 | < 2.00.10^-16^ *** |
| 1998 | -1.39 | 0.16 | -8.45 | < 2.00.10^-16^ *** |
| 1999 | -1.46 | 0.16 | -8.89 | < 2.00.10^-16^ *** |
| 2000 | -1.56 | 0.16 | -9.48 | < 2.00.10^-16^ *** |
| 2001 | -1.59 | 0.16 | -9.71 | < 2.00.10^-16^ *** |
| 2002 | -1.71 | 0.16 | -10.40 | < 2.00.10^-16^ *** |
| 2003 | -1.72 | 0.16 | -10.48 | < 2.00.10^-16^ *** |
| 2004 | -1.73 | 0.16 | -10.58 | < 2.00.10^-16^ *** |
| 2005 | -1.61 | 0.16 | -9.81 | < 2.00.10^-16^ *** |
| 2006 | -1.59 | 0.16 | -9.70 | < 2.00.10^-16^ *** |
| 2007 | -1.71 | 0.16 | -10.41 | < 2.00.10^-16^ *** |
| 2008 | -1.66 | 0.16 | -10.13 | < 2.00.10^-16^ *** |
| 2009 | -1.63 | 0.16 | -9.92 | < 2.00.10^-16^ *** |
| 2010 | -1.54 | 0.16 | -9.40 | < 2.00.10^-16^ *** |

Significance codes: 0 ‘***’ 0.001 ‘**’ 0.01 ‘*’ 0.05 ‘.’ 0.1 ‘’ 1

Adjusted R-squared: 0.2328

1. *Residuals analysis*

- **Statistically, time-series have an effect on the global TCI trend which confirm that the global change in the trend is explained by the time and is not random or noise**
- **Dispersion of residuals in the intervals is good and among years are homogenous: validation**
- **23% of the data variability is explained by the model and almost all the years have a significant negative effect (sign of the estimates) which confirms a global decreasing trend, significantly different than noise**

Efficiency Cumulated Indicator global trend:

1. *Data*


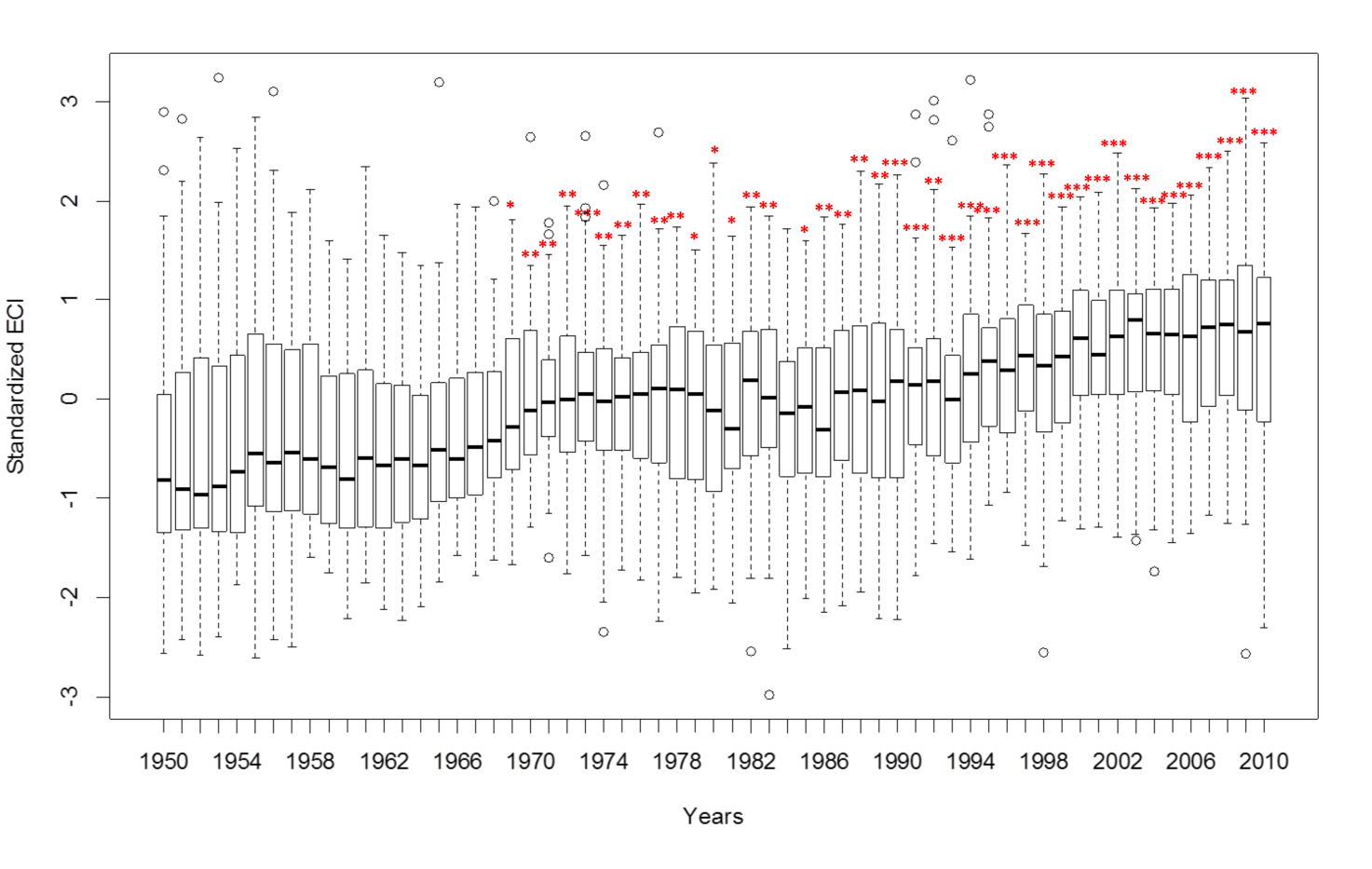


1. *Test of factor ‘years’ through a variance analysis*

|  | **Degrees of freedom** | **Sum Sq** | **Mean Sq** | **F value** | **Pr (>F)** |
| --- | --- | --- | --- | --- | --- |
| Factor ‘years’ | 60 | 388.83 | 6.4805 | 7.3177 | < 2.20.10^-16^ *** |
| Residuals | 3355 | 2971.17 | 0.8856 |  |  |

Significance codes: 0 ‘***’ 0.001 ‘**’ 0.01 ‘*’ 0.05 ‘.’ 0.1 ‘’ 1

1. *Interpretation of coefficients*

Model formula: lm(ECI ~ years)

| **Factor** | **Estimate** | **Std. Error** | **t value** | **Pr (>\|t\|)** |
| --- | --- | --- | --- | --- |
| Intercept | -0.50 | 0.13 | -4.01 | 6.32.10^-5^ *** |
| 1951 | -0.01 | 0.18 | -0.07 | 0.944 |
| 1952 | 0.08 | 0.18 | 0.46 | 0.644 |
| 1953 | 0.09 | 0.18 | 0.51 | 0.614 |
| 1954 | 0.14 | 0.18 | 0.78 | 0.434 |
| 1955 | 0.23 | 0.18 | 1.29 | 0.197 |
| 1956 | 0.23 | 0.18 | 1.30 | 0.193 |
| 1957 | 0.21 | 0.18 | 1.17 | 0.241 |
| 1958 | 0.18 | 0.18 | 1.02 | 0.308 |
| 1959 | 0.08 | 0.18 | 0.44 | 0.660 |
| 1960 | 0.01 | 0.18 | 0.07 | 0.948 |
| 1961 | 0.06 | 0.18 | 0.35 | 0.729 |
| 1962 | -0.05 | 0.18 | -0.29 | 0.776 |
| 1963 | 0.03 | 0.18 | 0.14 | 0.887 |
| 1964 | 0.02 | 0.18 | 0.11 | 0.915 |
| 1965 | 0.19 | 0.18 | 1.08 | 0.278 |
| 1966 | 0.20 | 0.18 | 1.12 | 0.264 |
| 1967 | 0.23 | 0.18 | 1.27 | 0.203 |
| 1968 | 0.26 | 0.18 | 1.45 | 0.147 |
| 1969 | 0.38 | 0.18 | 2.15 | 0.032 * |
| 1970 | 0.55 | 0.18 | 3.09 | 0.002 ** |
| 1971 | 0.57 | 0.18 | 3.18 | 0.001 ** |
| 1972 | 0.57 | 0.18 | 3.21 | 0.001 ** |
| 1973 | 0.63 | 0.18 | 3.53 | 0.0004 *** |
| 1974 | 0.52 | 0.18 | 2.91 | 0.004 ** |
| 1975 | 0.50 | 0.18 | 2.83 | 0.004 ** |
| 1976 | 0.52 | 0.18 | 2.93 | 0.003 ** |
| 1977 | 0.52 | 0.18 | 2.92 | 0.004 ** |
| 1978 | 0.48 | 0.18 | 2.70 | 0.007 ** |
| 1979 | 0.43 | 0.18 | 2.39 | 0.017 * |
| 1980 | 0.35 | 0.18 | 1.96 | 0.050 * |
| 1981 | 0.37 | 0.18 | 2.06 | 0.040 * |
| 1982 | 0.55 | 0.18 | 3.08 | 0.002 ** |
| 1983 | 0.46 | 0.18 | 2.60 | 0.009 ** |
| 1984 | 0.30 | 0.18 | 1.70 | 0.089 . |
| 1985 | 0.42 | 0.18 | 2.37 | 0.018 * |
| 1986 | 0.34 | 0.18 | 1.94 | 0.052 . |
| 1987 | 0.48 | 0.18 | 2.72 | 0.007 ** |
| 1988 | 0.57 | 0.18 | 3.18 | 0.001 ** |
| 1989 | 0.54 | 0.18 | 3.01 | 0.003 ** |
| 1990 | 0.53 | 0.18 | 3.00 | 0.003 ** |
| 1991 | 0.61 | 0.18 | 3.44 | 0.0006 *** |
| 1992 | 0.67 | 0.18 | 3.76 | 0.0002 *** |
| 1993 | 0.53 | 0.18 | 2.98 | 0.003 ** |
| 1994 | 0.78 | 0.18 | 4.40 | 1.14.10^-5^ *** |
| 1995 | 0.84 | 0.18 | 4.73 | 2.34.10^-6^ *** |
| 1996 | 0.77 | 0.18 | 4.35 | 1.43.10^-5^ *** |
| 1997 | 0.82 | 0.18 | 4.62 | 4.08.10^-6^ *** |
| 1998 | 0.72 | 0.18 | 4.04 | 5.37.10^-5^ *** |
| 1999 | 0.87 | 0.18 | 4.89 | 1.05.10^-6^ *** |
| 2000 | 1.00 | 0.18 | 5.62 | 2.10.10^-8^ *** |
| 2001 | 0.88 | 0.18 | 4.94 | 8.34.10^-7^ *** |
| 2002 | 1.04 | 0.18 | 5.84 | 5.79.10^-9^ *** |
| 2003 | 1.08 | 0.18 | 6.09 | 1.26.10^-9^ *** |
| 2004 | 1.07 | 0.18 | 6.03 | 1.85.10^-9^ *** |
| 2005 | 0.99 | 0.18 | 5.57 | 2.75.10^-8^ *** |
| 2006 | 1.05 | 0.18 | 5.91 | 3.73.10^-9^ *** |
| 2007 | 1.09 | 0.18 | 6.11 | 1.11.10^-9^ *** |
| 2008 | 1.08 | 0.18 | 6.09 | 1.25.10^-9^ *** |
| 2009 | 1.10 | 0.18 | 6.17 | 7.56.10^-10^ *** |
| 2010 | 0.98 | 0.18 | 5.54 | - - - 1. *** |

Significance codes: 0 ‘***’ 0.001 ‘**’ 0.01 ‘*’ 0.05 ‘.’ 0.1 ‘’ 1

Adjusted R-squared: 0.09991

1. *Residuals analysis*

- **Statistically, time-series have an effect on the global ECI trend which confirm that the global change in the trend is explained by the time and is not random or noise**
- **Dispersion of residuals in the intervals is good and among years are homogenous: validation**
- **9.9% of the data variability is explained by the model and the years after 1968 have a significant positive effect (sign of the estimates) which confirms a global increasing trend**
