# Supplementary material for: Global change in the trophic functioning of marine food webs
Source: PLoS One. 2017 Aug 11;12(8):e0182826. doi: 10.1371/journal.pone.0182826 (PMC5553640; doi:10.1371/journal.pone.0182826)
Supplement: S6 Appendix — (DOCX) [file pone.0182826.s008.docx]

S6 Appendix. Complementary results of the clustering performed on TCI_R_.

Statistical test of supplementary qualitative variables in the clustering performed on TCI_R_.

| **Cluster** | **Main significant modalities of the qualitative supplementary variables** | **% of occurrence in total** | **% of**  **occurrence in cluster** | **Global** | **p.value** |
| --- | --- | --- | --- | --- | --- |
| **1 16LMEs** | Fraction of cephalopods stable  FiB decreasing  High SSP  PPR/PP decreasing  MTL decreasing  Recent SSP: 50-75%  Fraction of fishes decreasing  Recent L_index_ low  Low catch  Fraction of fishes <80%  Polar ecosystems  Low L_index_  Fraction of fishes >90%  Fraction of cephalopods increase  High recent catch  Very high recent L_index_  PPR/PP increase  MTL stable  FiB increase  SSP moderate  Recent SSP : 25-50% | 85.7  100.0  52.6  80.0  44.0  41.4  39.4  50.0  55.6  50.0  50.0  46.7  16.7  22.7  17.2  10.0  22.5  13.8  21.3  12.2  8.3 | 37.5  25.0  62.5  25.0  68.8  75.0  81.3  43.8  31.3  37.5  37.5  43.8  25.0  62.5  31.3  12.5  68.8  25.0  62.5  37.5  12.5 | 12.5  7.1  33.9  8.9  44.6  51.8  58.9  25.0  16.1  21.4  21.4  26.8  42.8  78.6  51.8  35.7  87.5  51.8  83.9  66.1  42.9 | 0.002**  0.005**  0.007**  0.021*  0.027*  0.033*  0.036*  0.057  0.076  0.087  0.087  0.090  0.098  0.087  0.061  0.023*  0.018*  0.013*  0.013*  0.007**  0.004** |
| **2 21LMEs** | MTL stable  Moderate TCI  Tropical ecosystems  High TCI  Fraction of cephalopods stable  Polar ecosystems  MTL decrease | 51.7  51.9  51.9  17.7  0.0  8.3  20.0 | 71.4  66.7  66.7  14.3  0.0  4.8  23.8 | 51.8  48.2  48.2  30.4  12.5  21.4  44.6 | 0.027*  0.038*  0.038*  0.047*  0.029*  0.019*  0.018* |
| **3**  **17LMEs** | Very high recent L_index_  High recent catch  Very high catch  Fraction of fish stable  High L_index_  PPR/PP increase  Fraction of shrimps: 0-1%  Recent PPR/PP<10%  Moderate recent catch  PPR/PP<10%  Low recent L_index_  Fraction of fish decrease  Low L_index_ | 55.0  44.8  53.3  50.0  50.0  34.7  45.0  15.8  15.0  15.0  7.1  18.2  6.7 | 64.7  76.5  47.1  52.9  47.1  100.0  52.9  17.7  17.7  17.7  5.9  35.3  5.9 | 35.7  51.8  26.8  32.1  28.6  87.5  35.7  33.9  35.7  35.7  25.0  58.9  26.8 | 0.004**  0.017*  0.034*  0.038*  0.057  0.066  0.091  0.098  0.069  0.069  0.029*  0.023*  0.019* |
| **4**  **2LMEs** | High MTL  SST stable  Polar ecosystems  High TCI | 18.2  100.0  16.7  11.8 | 100.0  50.0  100.0  100.0 | 19.6  1.8  21.4  30.4 | 0.036*  0.036*  0.043*  0.088 |

Qualitative variables modalities that explain the cluster common patterns *‘*’ stands for p.value<0.05, ‘**’ for p.value<0.01, ‘***’ for p.value<0.001*

Statistical tests of supplementary quantitative variables in the clustering performed on TCI_R_.

| **Cluster** | **Main signifiant quantitative supplementary variables** | **Mean in category** | **Overall**  **Mean** | **p.value** |
| --- | --- | --- | --- | --- |
| **1 16LMEs** | SSP  Fraction of shrimps  Recent SSP  Mean TCI  Recent L_index_  Mean recent catch  Recent PPR/PP  Mean fraction of fish relative to 1950 | 28.9  7.3  56.9  2.99  0.02  0.7  12.4  0.90 | 23.0  3.9  50.2  2.66  0.05  1.6  29.5  0.98 | 0.006**  0.007**  0.034*  0.073  0.054  0.042*  0.024*  0.0002*** |
| **2 21LMEs** | Mean fraction of fish relative to 1950  Mean MTL relative to 1950  Mean correlation to Shannon | 1.02  1.01  -0.46 | 0.98  0.99  -0.33 | 0.029*  0.062  0.071 |
| **3**  **17LMEs** | Recent L_index_  Mean recent catch  Recent PPR/PP | 0.063  2.3  42.7 | 0.044  1.6  29.5 | 0.042*  0.055  0.072 |
| **4**  **2LMEs** | Mean TCI  Recent PPR/PP | 4.10  71.9 | 2.66  29.5 | 0.017*  0.091 |

Quantitative variables modalities that explain the cluster common patterns *‘*’ stands for p.value<0.05, ‘**’ for p.value<0.01, ‘***’ for p.value<0.001*
