# Supplementary material for: Global change in the trophic functioning of marine food webs
Source: PLoS One. 2017 Aug 11;12(8):e0182826. doi: 10.1371/journal.pone.0182826 (PMC5553640; doi:10.1371/journal.pone.0182826)
Supplement: S7 Appendix — (DOCX) [file pone.0182826.s009.docx]

S7 Appendix. Complementary results of the clustering performed on ECI_R_.

Statistical tests of supplementary qualitative variables in the clustering performed on ECI_R_.

| **Cluster** | **Main significant modalities of the qualitative supplementary variables** | **% of occurrence in the cluster** | **% of global occurrence** | **Global** | **p.value** |
| --- | --- | --- | --- | --- | --- |
| **1**  **7LMEs** | TCI increase  TCI stable  PP stable  Low L_index_  Moderate ECI  Recent PPR/PP<10%  PPR/PP<10%  TCI decrease | 66.7  28.6  0.0  0.0  0.0  0.0  0.0  2.6 | 28.6  57.1  0.0  0.0  0.0  0.0  0.0  14.3 | 5.4  25.0  26.8  26.8  30.4  33.9  35.7  69.6 | 0.040*  0.066  0.097  0.097  0.066  0.044*  0.036*  0.002** |
| **2**  **29LMEs** | Recent PPR/PP>50%  Moderate ECI  +0.1°C< SST increase <+0.7°C  Temperate ecosystems  Recent very high L_index_  Fraction of cephalopods : 1-5%  0< Correlation to Shannon <0.5  Moderate recent catch  +0.7°C< SST increase <2°C  Recent PPR/PP: 10-25%  FiB decrease  Fraction of cephalopods: 0-1%  Low ECI | 90.0  76.5  65.6  76.9  70.0  65.4  65.4  35.0  35.0  28.6  0.0  34.6  27.8 | 31.0  44.8  72.4  34.5  48.3  58.6  48.3  24.1  24.1  13.8  0.0  31.0  17.2 | 17.9  30.4  57.1  23.2  35.7  46.4  37.5  35.7  35.7  25.0  7.1  46.4  32.1 | 0.009**  0.017*  0.020*  0.045*  0.049*  0.066  0.095  0.070  0.070  0.054  0.048*  0.020*  0.016* |
| **3**  **10LMEs** | Catch increase+  Tropical ecosystems  Low ECI  Catch increase++  Fraction of fishes <80%  Temperate ecosystems | 32.1  29.6  33.3  5.3  0.0  0.0 | 90.0  80.0  60.0  10.0  0.0  0.0 | 50.0  48.2  32.1  33.9  21.4  23.2 | 0.006**  0.033*  0.055  0.086  0.070  0.054 |
| **4**  **9LMEs** | Fraction of fishes decrease  FiB decrease  Fraction of fishes <80%  PPR/PP decrease  TCI decrease  Recent PPR/PP<10%  Correlation to Shannon >0.5  Recent L_index_  PPR/PP<10%  Fraction of cephalopods <1%  Fraction of shrimps 5-40%  Low L_index_  TCI stable  FiB increase  Fraction of fishes stable  PPR/PP increase  0< Correlation to Shannon <0.5 | 27.3  75.0  41.7  60.0  23.1  32.6  31.6  35.7  30.0  26.9  33.3  33.3  0.0  10.6  0.0  10.2  0.0 | 100.0  33.3  55.6  33.3  100.0  66.7  66.7  55.6  66.7  77.8  55.6  55.6  0.0  55.6  0.0  55.6  0.0 | 58.9  7.1  21.4  8.9  69.6  33.9  33.9  25.0  35.7  46.4  26.8  26.8  25.0  83.9  32.1  87.5  37.5 | 0.0051**  0.011*  0.018*  0.027*  0.028*  0.037*  0.040*  0.040*  0.051  0.051  0.056  0.056  0.059  0.032*  0.022*  0.010*  0.009** |
| **5**  **1LME** | - | - | - | - | - |

Qualitative variables modalities that explain the cluster common patterns*‘*’ stands for p.value<0.05, ‘**’ for p.value<0.01, ‘***’ for p.value<0.001*

Statistical tests of supplementary quantitative variables in the clustering performed on ECI_R_.

| **Cluster** | **Main signifiant quantitative supplementary variables** | **Mean in category** | **Overall mean** | **p.value** |
| --- | --- | --- | --- | --- |
| **1**  **7LMEs** | TCI relative to 1950 | 1.14 | 0.93 | 0.00005*** |
| **2**  **29LMEs** | Mean fraction of fish relative to 1950  Recent L_index_  Recent PPR/PP  L_index_  Mean recent catch  Mean MTL  Fraction of shrimp species | 1.01  0.06  39.3%  0.048  2.0 t/km²  3.4  2.3% | 0.98  0.04  29.5%  0.038  1.6 t/km²  3.5  3.9% | 0.020*  0.031*  0.036*  0.075  0.081  0.096  0.037* |
| **3**  **10LMEs** | SST difference between 2010 and 1950 | 0.76 | 0.56 | 0.066 |
| **4**  **9LMEs** | Recent PPR/PP  TCI relative to 1950  Mean fraction of fish relative to 1950 | 8.3%  0.79  0.87 | 29.5%  0.93  0.98 | 0.054  0.002**  0.0002*** |
| **5**  **1LME** | - | - | - | - |

Quantitative variables modalities that explain the cluster common patterns*‘*’ stands for p.value<0.05, ‘**’ for p.value<0.01, ‘***’ for p.value<0.001*
